# Changes in Cigarette Consumption With Reduced Nicotine Content Cigarettes Among Smokers With Psychiatric Conditions or Socioeconomic Disadvantage: 3 Randomized Clinical Trials

**DOI:** 10.1001/jamanetworkopen.2020.19311

**Published:** 2020-10-20

**Authors:** Stephen T. Higgins, Jennifer W. Tidey, Stacey C. Sigmon, Sarah H. Heil, Diann E. Gaalema, Dustin Lee, John R. Hughes, Andrea C. Villanti, Janice Y. Bunn, Danielle R. Davis, Cecilia L. Bergeria, Joanna M. Streck, Maria A. Parker, Mollie E. Miller, Michael DeSarno, Jeff S. Priest, Patricia Cioe, Douglas MacLeod, Anthony Barrows, Catherine Markesich, Roxanne F. Harfmann

**Affiliations:** 1 UVM Tobacco Center of Regulatory Science, University of Vermont, Burlington; 2 Center for Alcohol and Addiction Studies, Brown University, Providence, Rhode Island; 3 Yale University Tobacco Center of Regulatory Science, Yale University School of Medicine, New Haven, Connecticut; 4 Behavioral Pharmacology Research Unit, Johns Hopkins University School of Medicine, Baltimore, Maryland; 5 Department of Psychiatry, Massachusetts General Hospital, Harvard Medical School, Boston; 6 School of Public Health, Indiana University, Bloomington

## Abstract

**Importance:**

This study is part of a programmatic effort evaluating the effects of reducing nicotine content of cigarettes to minimally addictive levels.

**Objective:**

To examine whether very low-nicotine-content (VLNC) cigarettes decrease smoking rates and dependence severity among smokers with psychiatric disorders or socioeconomic disadvantage.

**Design, Setting, and Participants:**

These 3 randomized clinical trials were performed at the University of Vermont, Brown University, and Johns Hopkins University between October 2016 and September 2019. Participants received 12 weeks of exposure to study cigarettes with nicotine content ranging from levels representative of commercial cigarettes (15.8 mg nicotine/g tobacco) to less than a hypothesized addiction threshold (2.4 mg/g and 0.4 mg/g). Daily smokers from 3 at-risk populations participated: individuals with affective disorders, exemplifying smokers with mental illness; individuals with opioid use disorder, exemplifying smokers with substance use disorders; and women with high school educations or less, exemplifying smokers with socioeconomic disadvantage. Data were analyzed from September 2019 to July 2020.

**Interventions:**

Random assignment to 1 of 3 study cigarettes provided weekly at no cost for 12 weeks.

**Main Outcomes and Measures:**

The primary outcome was between-group differences in mean total cigarettes smoked daily (CPD) during week 12; secondary outcomes included CPD for study and nonstudy cigarettes and dependence severity across weeks analyzed using analysis of covariance, random coefficients growth modeling, or repeated measures analysis of variance.

**Results:**

A total of 775 participants were included (mean [SD] age, 35.59 [11.05] years; 551 [71.10%] women [owing to 1 population being exclusively women]); participants smoked a mean (SD) of 17.79 (9.18) CPD at study intake. A total of 286 participants were randomized to 0.4 mg/g, 235 participants were randomized to 2.4 mg/g, and 254 participants were randomized to 15.8 mg/g. Participants randomized to VLNC cigarettes had decreased mean [SEM] total CPD during week 12 across populations (Cohen *d* = 0.61; *P* < .001). At week 12, mean (SEM) CPD decreased to 17.96 (0.98) CPD in the 0.4 mg/g group and to 19.53 (1.07) CPD in the 2.4 mg/g group, both of which were significantly different from the 15.8 mg/g group (25.08 [1.08] CPD at week 12) but not each other (0.4 mg/g adjusted mean difference: −7.54 [95%CI, −9.51 to −5.57]; 2.4 mg/g adjusted mean difference: −5.34 [95% CI, 7.41 to −3.26]). Several secondary outcomes differed across populations randomized to VLNCs, including mean total CPD across weeks, with linear trends lower in participants receiving 0.4 mg/g (−0.28 [95%CI, −0.39 to −0.18]; *P* < .001) and 2.4 mg/g (−0.13 [95%CI, −0.25 to −0.01]; *P* < .001) doses compared with those receiving the 15.8 mg/g dose (0.30 [95% CI, 0.19 to 0.41]). Fagerström Test of Nicotine Dependence mean total scores were significantly lower in participants who received VLNCs (Cohen *d* = 0.12; *P* < .001), with those who received the 0.4 mg/g dose (mean [SD] score, 3.99 [0.06]; *P* < .001 vs 15.8 mg/g) or 2.4 mg/g dose (mean [SD] score, 4.07 [0.06]; *P* = .01 vs 15.8 mg/g) differing from those who received the 15.8 mg/g dose (mean [SD] score, 4.31 [0.06]) but not from each other.

**Conclusions and Relevance:**

These findings demonstrate that decreasing the nicotine content of cigarettes to very low levels reduced smoking rate and nicotine-dependence severity in these high-risk populations, effects that may facilitate successful cessation.

**Trial Registration:**

ClinicalTrials.gov Identifiers: NCT02232737, NCT02250664, NCT02250534

## Key Points

**Question:**

Does reducing the nicotine content of cigarettes decrease smoking rates and nicotine dependence severity among adults with psychiatric disorders or socioeconomic disadvantage?

**Findings:**

These 3 randomized clinical trials including 775 participants with affective disorders, opioid use disorder, or socioeconomic disadvantage found that reducing nicotine content significantly decreased total cigarettes smoked daily and nicotine dependence severity.

**Meaning:**

These results further demonstrate that reducing nicotine content of cigarettes to low levels has potential to benefit populations at high risk for tobacco use, addiction, and smoking-attributable morbidity and mortality.

## Teaser

These 3 randomized clinical trials examine the efficacy of very low-nicotine-content cigarettes in reducing smoking rates among smokers with affective disorders, opioid use disorders, or socioeconomic disadvantage.

## Introduction

Cigarette smoking places a disproportionate burden on individuals with psychiatric conditions and socioeconomic disadvantage.^1,2,3,4,5,6^ Studies in the general population of smokers demonstrate that reducing nicotine content in cigarettes to very low levels decreases smoking rate and dependence severity with minimal compensatory smoking (ie, smoking adjustments to sustain desired nicotine blood levels).^7,8,9,10,11,12,13,14^ Overrepresentation of smoking among populations with psychiatric conditions and socioeconomic disadvantage requires examination of how these high-risk populations respond to reduced nicotine–content cigarettes.^1,2,3,4,5,6,15,16,17,18,19,20,21,22,23^

Two controlled studies in smokers with serious mental illness (ie, schizophrenia, schizoaffective disorder) examined acute^16,17^ and extended (ie, 6-week)^18^ exposure to very low-nicotine-content (VLNC) cigarettes demonstrating reductions in cigarette use without compensatory smoking, although small sample sizes precluded examining dose or population differences. A third controlled study^19^ extended investigation of acute effects of VLNCs into populations with affective disorders, substance use disorders, and socioeconomic disadvantage. During acute exposure, VLNCs decreased the addiction potential (ie, reinforcing and positive subjective effects) of smoking in all populations without evidence of compensatory smoking. This study builds programmatically on that acute exposure study^19^ by investigating extended exposure to VLNCs in these same high-risk populations.

## Methods

### Study Design

This report includes data from 3 parallel, 12-week, double-blind randomized clinical trials. Trial protocols were identical across populations (except for study inclusion and exclusion criteria), reviewed and approved by local institutional review boards, and monitored by an independent data- and safety-monitoring board. Participants provided written informed consent. This study follows the Consolidated Standards of Reporting Trials (CONSORT) reporting guideline.

**Figure 1.**
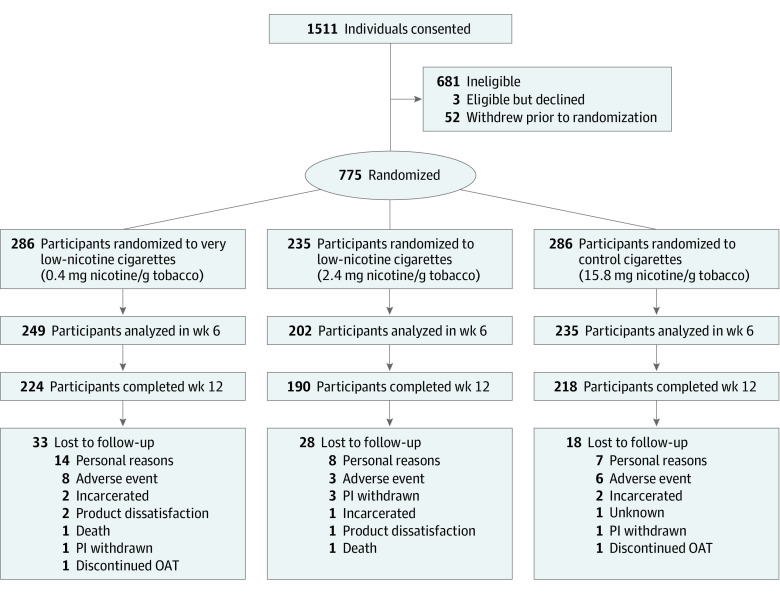
Participant Enrollment, Randomization, and Retention Flowchart OAT indicates opioid assisted treatment; PI, principal investigator.

Each trial was conducted at 2 of 3 sites (ie, University of Vermont, Brown University, and Johns Hopkins University) between October 2016 and September 2019 (Figure 1). Across trials, 775 adult, daily smokers not planning to quit in the next 30 days were recruited, including 258 adults with affective disorders; 260 adults with opioid use disorder (OUD); and 257 women with a high school education or less (as the socioeconomically disadvantaged group). Participants were randomly assigned to smoke 1 of 3 study cigarettes varying in nicotine content from a level comparable to commercial cigarettes (15.8 mg nicotine/g tobacco)^24^ to cigarettes with nicotine content at 15.2% (2.4 mg nicotine/g tobacco) or 2.5% (0.4 mg nicotine/g tobacco) of control-cigarette level.

### Study Cigarettes

The National Institute on Drug Abuse provided study cigarettes with 0.4, 2.4, or 15.8 mg of nicotine per 1 g of tobacco, averaged across menthol and nonmenthol cigarettes.^10,11,12,25^ Cigarettes were identical in appearance. Assignment to menthol or nonmenthol was based on participant preference.

### Participants

Participants were recruited through newspaper and online ads, community-bulletin boards, and word-of-mouth referrals. Shared inclusion criteria across trials were age 18 years or older, smoked 5 or more cigarettes per day (CPD) for past year, breath carbon monoxide (CO) sample greater than 8 ppm, no psychiatric conditions with potential to interfere with study results or completion, sufficiently literate to complete study tasks, no serious illness or negative health changes in the past 90 days, no use of tobacco or nicotine products other than cigarettes on more than 9 days in the past 30 days, and in women, not pregnant or nursing and reporting current use of contraception or history of surgical sterilization or being postmenopausal. Shared exclusion criteria included prior regular use of VLNC cigarettes, plans to quit smoking in the next 30 days, past-month cessation attempt in which abstinence exceeded 3 days, exclusive use of roll-your-own cigarettes, positive toxicology test results for drugs other than cannabis, breath alcohol level greater than 0.01, and recent suicide ideation or attempt.

For the trials focused on psychiatric disorders, inclusion criteria included meeting diagnostic criteria for an affective disorder^26^ or OUD with stable enrollment in opioid-substitution therapy. Population-specific exclusion criteria included age older than 70 years in trials on smokers with psychiatric disorders and older than 44 years in the trial on women who were disadvantaged; comorbid substance use disorder in trials examining affective disorders and women who were disadvantaged; and anticonvulsant use in trials with OUD and women who were disadvantaged. Participants were compensated up to $2601 for participation. Age, sex, and race/ethnicity (self-identified using investigator-provided categories) were collected per National Institutes of Health guidelines using a demographic screening questionnaire. Additional details on participant characteristics, inclusion and exclusion criteria, and compensation are provided in the Trial Protocol in Supplement 1 and eFigure 1, eTable 2, and eTable 3 in Supplement 2.

### Procedures

Eligible participants were assigned to 1 of 3 study-cigarette doses and completed a 2-visit, 1-week baseline examination. During first baseline visit, they received a free supply of usual-brand cigarettes for use during the subsequent week to establish baseline smoking rate.^10,12^ The supply was 150% of self-reported smoking rate to accommodate increases.^14^ Participants used an interactive voice response system (Telesage) daily throughout the study to report prior-day cigarette consumption, other tobacco or nicotine use, and nicotine-withdrawal symptoms. Interactive voice response adherence was compensated at $1.00 per call plus $10.00 bonuses for 7-day consecutive calls. A randomly determined subset of participants completed baseline and week 12 neuroimaging sessions.

Participants received the first supply of study cigarettes at the second baseline session. They reported to the clinic weekly for next 12 weeks to return unused study cigarettes and be resupplied. Participants received 2-fold the number of cigarettes used during baseline to accommodate smoking rate increases or missed visits.^10,11,12^ They were counseled on using only study cigarettes, managing adherence difficulties, queried about plans to quit smoking, and offered a referral for smoking cessation if they wanted to quit.

Weekly visits also included biochemical and self-report assessments of recent tobacco and drug use, vital signs, pregnancy status, health and medication use, mood and anxiety, and adverse events (AEs). At the second baseline visit and week 2, 6, and 12 visits, first-void morning urine specimens (a spot sample was collected if a first-void sample was not collected), and blood and breath samples were collected to assess nicotine and toxin exposure, lung function, and nicotine-metabolite ratio. At those visits, participants completed a respiratory health questionnaire,^10^ cognitive performance assessments, and smoked a single usual-brand (baseline) or study cigarette through a handheld device (Borgwaldt) measuring puff topography.

At the week 12 visit, participants could earn $100 by abstaining from smoking for the next 24 hours (measured as breath CO ≤4 ppm) to determine whether VLNCs altered ability to abstain.^27^ Approximately 30 days after completion of the week 12 visit, participants were contacted via telephone to assess smoking status.^10^

### Outcomes

The primary outcome was mean total CPD (study and nonstudy cigarettes) during week 12, based on interactive voice response assessments, an indicator of the reinforcing value of smoking.^10,11,12^ Secondary CPD outcomes included changes in total, study, and nonstudy CPD across weeks. Other secondary outcomes included nicotine dependence severity based on Fagerström Test for Nicotine Dependence^28^ total scores (minus CPD item) and Wisconsin Inventory of Smoking Dependence Motives (Brief WISDM) total and primary dependence motive and secondary dependence motive subscale scores,^29^ breath CO,^30^ urinary cotinine and NNAL (marker of tobacco-specific n-nitrosamine exposure) level,^31,32,33,34,35^ Cigarette Purchase Task,^36,37,38,39^ smoking topography,^40^ craving or withdrawal using Questionnaire on Smoking Urges Brief (QSU-Brief)^41^ and Minnesota Tobacco Withdrawal Scale^42^ scores, and during-study and poststudy abstinence outcomes.^10,12,27^

Exploratory outcomes, including modified Cigarette Evaluation Questionnaire ratings,^43^ harm-perceptions scores,^11^ Smoking Stages-of-Change scores,^44^ nicotine-metabolite ratio,^35^ fractional exhaled nitric oxide testing,^45^ neuroimaging,^46,47,48^ biomarkers of cardiovascular function,^49^ and cognitive test performance^50,51^ will be reported separately.

### Statistical Analysis

Analysis of covariance was used for total and study CPD during week 12, adjusting for baseline values. Additional covariates included age, sex, menthol cigarette use, and at-risk population. Secondary outcomes were analyzed using linear mixed models. Outcomes assessed weekly or biweekly used a growth curve model, assuming an unstructured covariance matrix. Analysis of non-study CPD relied on a negative reciprocal transformation of the time variable to allow modeling of a linear trend. All other time trends were fit with a linear growth model. Outcomes assessed less frequently were analyzed using repeated measures analysis of variance, assuming compound symmetry. Initial models included at-risk population by condition or population by condition by time interactions as appropriate, which were removed if not significant, with 2-sided α at *P* < .05. Significant effects were followed with pairwise comparisons using Bonferroni correction. Participants not completing the study were excluded in analyses of covariance models but included in linear mixed models with multiple imputation used to assess the effect of missing data. Number of AEs and days abstinent were compared using 0-inflated negative binomial regression, and quit attempts and ability to abstain were compared using logistic regression. See Supplement 1 for Trial Protocol and additional details on statistical methods. All analyses were conducted using SAS statistical software version 9.4 (SAS Institute). Data were analyzed from September 2019 to July 2020.

## Results

### Participants

A total of 775 participants were included (mean [SD] age, 35.59 [11.05] years; 551 [71.10%] women [owing to one population being exclusively women]); participants smoked a mean (SD) of 17.79 (9.18) CPD at study intake. A total of 286 participants were randomized to 0.4 mg nicotine/g tobacco, 235 participants were randomized to 2.4 mg nicotine/g tobacco, and 254 participants were randomized to 15.8 mg nicotine/g tobacco. The only significant difference among dose conditions in baseline characteristics was preference for menthol cigarettes, which was a covariate in all analyses (Table). Most randomized smokers (642 smokers [82.8%]) completed the study. Participants who dropped out, compared with those who did not, were younger (mean [SD] age, 32.17 [9.81] years vs 36.30 [11.17] years) and less likely to be married (10 participants [7.5%] vs 102 participants [15.9%]) (eTable 1 in Supplement 2) but did not differ significantly by dose or at-risk population.

**Table.** Demographic and Smoking Characteristics Across Populations

Characteristic	Participants, No. (%)			
	Overall (N = 775)	Dose, mg nicotine/g tobacco		
		0.4 (n = 286)	2.4 (n = 235)	15.8 (n = 254)
Population				
Women with disadvantage^a^	257 (33.16)	93 (32.52)	81 (34.47)	83 (32.68)
Opioid use disorder	260 (33.55)	92 (32.17)	81 (34.47)	87 (34.25)
Affective disorders	258 (33.29)	101 (35.31)	73 (31.06)	84 (33.07)
Age, mean (SD), y	35.59 (11.05)	35.65 (11.21)	35.18 (11.13)	35.90 (10.82)
Women	551 (71.10)	200 (69.93)	167 (71.06)	184 (72.44)
Race/ethnicity				
Non-Latino				
White	630 (82.14)	240 (84.81)	183 (78.21)	207 (82.80)
Black	68 (8.87)	19 (6.71)	25 (10.68)	24 (9.60)
Latino	23 (3.00)	8 (2.83)	8 (3.42)	7 (2.80)
Non-Latino other or >1 race	46 (6.00)	16 (5.65)	18 (7.69)	12 (4.80)
Education				
<High school	102 (13.16)	35 (12.24)	32 (13.62)	35 (13.78)
High school graduate or equivalent or some college	570 (73.55)	221 (77.27)	167 (71.06)	182 (71.65)
Associate’s degree	38 (4.90)	12 (4.20)	14 (5.96)	12 (4.72)
≥College graduate	65 (8.39)	18 (6.29)	22 (9.36)	25 (9.84)
Marital status				
Married	112 (14.45)	34 (11.89)	33 (14.04)	45 (17.72)
Never married	461 (59.48)	172 (60.14)	145 (61.70)	144 (56.69)
Divorced, separated, or widowed	202 (26.06)	80 (27.97)	57 (24.26)	65 (25.59)
Primary smoker of mentholated cigarettes	351 (45.29)	114 (39.86)^b^	120 (51.06)^b^	117 (46.06)^b^
Cigarettes smoked/d, mean (SD), No.	17.79 (9.18)	18.16 (9.59)	17.23 (8.66)	17.90 (9.18)
Urine cotinine level, mean (SD), ng/mL	4929.35 (3771.79)	4858.18 (3725.39)	4898.10 (3895.50)	5037.62 (3719.37)
NMR ≥0.31	526 (73.06)	193 (71.75)	152 (71.70)	181 (75.73)
Breath carbon monoxide level, mean (SD), ppm	18.02 (9.85)	17.99 (10.32)	17.65 (9.30)	18.38 (9.83)
Age started smoking regularly, mean (SD), y	16.14 (3.97)	16.13 (4.15)	16.23 (3.63)	16.07 (4.07)
Fagerström Test for Cigarette Dependence score, mean (SD)	5.56 (2.35)	5.54 (2.33)	5.55 (2.32)	5.59 (2.42)
Heaviness of Smoking Index score, mean (SD)	3.49 (1.54)	3.48 (1.59)	3.48 (1.51)	3.50 (1.53)
Used other tobacco products, last 30 d	117 (17.67)	42 (17.21)	37 (18.05)	38 (17.84)

Abbreviation: NMR, nicotine metabolite ratio.

^a^ In this study, having a high school education or less was considered the proxy for socioeconomic disadvantage.

^b^ Significant difference between dose conditions in preference for mentholated cigarettes (χ^2^ = 6.63; *P* = .04).

### Cigarettes Smoked per Day

**Figure 2.**
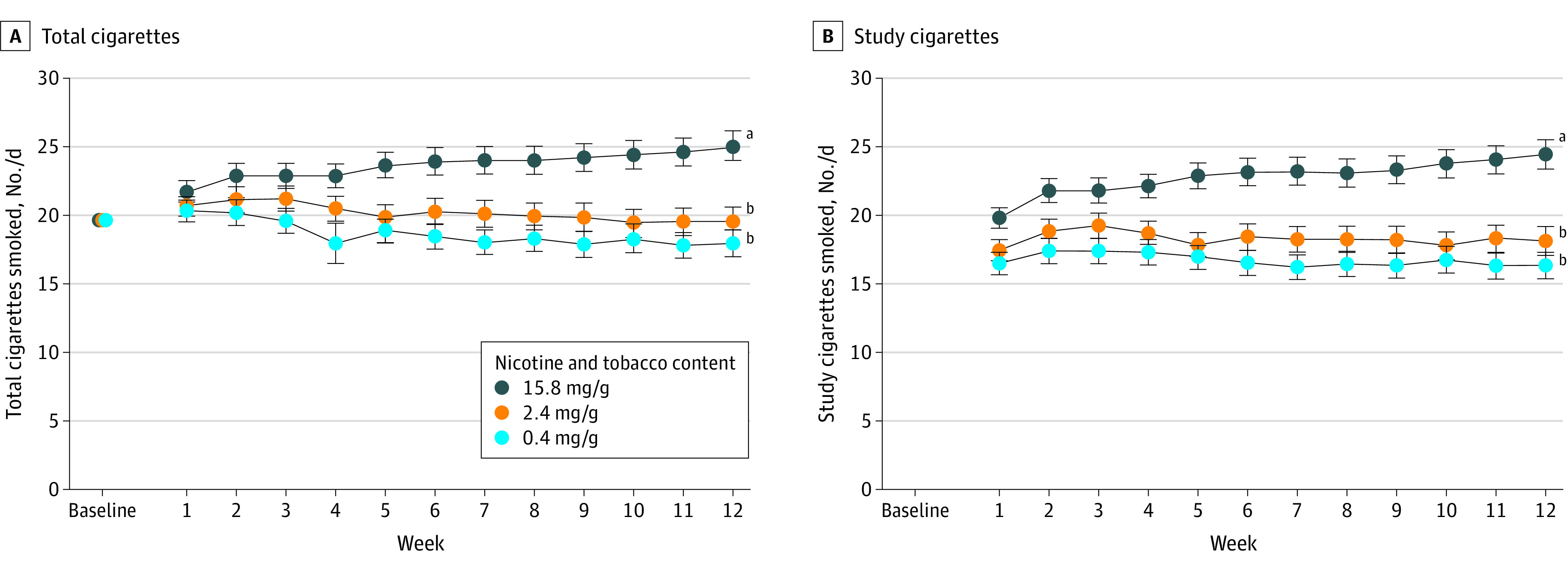
Number of Total and Study Cigarettes Smoked per Day According to Nicotine Content Data points are arithmetic means collapsed across participants and populations; error bars indicate SEM. Data not sharing a superscript letter differed significantly in post hoc testing and in slope of linear trends.

Total CPD during week 12 across populations decreased (Cohen *d* = 0.61; P < .001) among participants who received 0.4 mg/g cigarettes or 2.4 mg/g cigarettes (Figure 2A). More specifically, compared with participants who received 15.8 mg/g cigarettes, CPD rates decreased significantly among participants who received 0.4 mg/g (adjusted mean difference, −7.54 [95% CI, −9.51 to −5.57]) or 2.4 mg/g (adjusted mean difference, −5.33 [95% CI, −7.41 to −3.26]) cigarettes, with no significant difference between VLNC cigarettes. Imputed adjusted mean differences were shifted slightly downward (0.4 mg/g: −6.01 [95% CI, −7.98 to −4.05]; 2.4 mg/g: −4.30 [95% CI, −6.39 to −2.21]). Effects on study CPD during week 12 closely paralleled those on total CPD (Cohen *d* = 0.68; *P* < .001) (Figure 2B).

Both VLNC conditions also decreased total CPD across weeks compared with the 15.8 mg/g condition across populations (Cohen *d* = 0.17; 0.4 mg/g vs 15. 8 mg/g: *P* < .001; 2.4 mg/g vs 15. 8 mg/g: *P* < .001) (Figure 2A), with decreasing linear trends at 0.4 mg/g (−0.28 [95% CI, −0.39 to −0.18]) and 2.4 mg/g (−0.13 [95% CI, −0.25 to −0.01]) doses and increasing trend at the 15.8 mg/g dose (0.30 [95% CI, 0.19 to 0.41]). The same pattern of effects was seen for study cigarettes across weeks (Cohen *d* = 0.13; .4 mg/g vs 15. 8 mg/g: *P* < .001; 2.4 mg/g vs 15. 8 mg/g: *P* < .001) (Figure 2B). Effects of 0.4 mg/g and 2.4 mg/g doses did not differ significantly from each other on total or study CPD.

Effects on nonstudy CPD varied by dose, time, and population (Cohen *d* = 0.07; *P* = .04) (eFigure 1 in Supplement 2). Across populations, nonstudy CPD was greater at 0.4 mg/g and 2.4 mg/g doses than at the 15.8 mg/g dose. Among smokers assigned to 0.4 mg/g cigarettes, those with OUD smoked more and evidenced a steeper decreasing trend across weeks (weekly change, −4.42 [95% CI, −5.38 to −3.46] CPD × study week) than smokers with affective disorders (weekly change, −0.90 [95% CI, −1.81 to 0.01] CPD × study week) or women with disadvantage (weekly change, −2.02 [95% CI, −2.99 to −1.04] CPD × study week).

### Use of Other Tobacco Products

Overall, 80 participants (10.4%) reported using e-cigarettes, 85 participants (11.0%) reported using smokeless tobacco, and 57 participants (7.4%) reported using nicotine replacement at least once during the study. Proportion of days using other tobacco products interacted with dose and population across e-cigarettes (Cohen *d* = 0.08; *P* = .01), smokeless tobacco (Cohen *d* = 0.10; *P* < .001), and NRT (Cohen d=0.07; *P* = .04). There were no significant differences between populations in use of these products at the 0.4 mg/g or 2.4 mg/g doses. However, at the 15.8 mg/g dose, those with OUD used e-cigarettes more frequently (0.03% [95% CI, 0.02%-0.04%] of days/week) compared with women with disadvantage (0 [95% CI, 0%-0.01%]; *P* = .001) and those with affective disorders (0.01% [95% CI, 0%-0.02%] of days/week; *P* = .008); smokeless tobacco more frequently (0.02% [95% CI, 0.01%-0.02%] of days/week) compared with women with disadvantage (0% [95% CI, 0%-0.01%] of days/week; *P* = .02) and those with affective disorders (0% [95% CI, 0%-0.01%] of days/week; *P* = .001); and NRT more frequently (0.02% [95% CI, 0.01%-0.03%] of days/week) compared with women with disadvantage (0% [95% CI, 0%-0.01%] of days/week; *P* = .01) and those with affective disorders (0% [95% CI, 0%-0.01%] of days/week; *P* = .01).

### Dependence Severity

**Figure 3.**
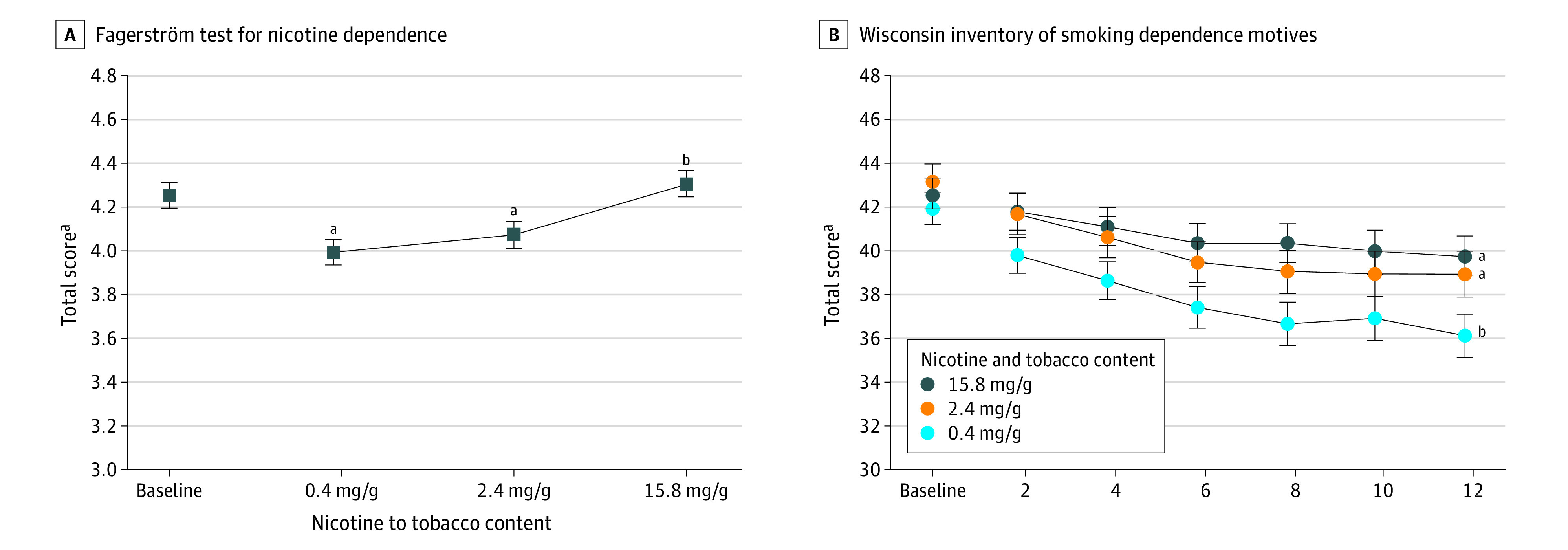
Changes in Nicotine Dependence Severity A, Data points are arithmetic means collapsed across participants, time, and populations; error bars indicate SEM. Data points not sharing a superscript letter differed significantly by dose in post hoc testing. B, Data points are arithmetic means collapsed across participants and populations at each assessment; error bars indicate SEM. Doses not sharing a superscript letter differed in slope of linear trends.

VLNCs decreased Fagerström Test for Nicotine Dependence total scores (minus Item 4 on CPD) by dose (Cohen *d* = 0.12; *P* < .001) (Figure 3A), with lower scores among participants randomized to 0.4 mg/g and 2.4 mg/g doses than those randomized to the 15.8 mg/g dose. Total WISDM scores varied by dose and time (Cohen *d* = 0.07; *P* = .04) (Figure 3B), with steeper decreasing trends at the 0.04 mg/g dose (WISDM score, −0.32 [95% CI, −0.42 to −0.22]) than at the 15.8 mg/g dose (WISDM score, −0.14 [95% CI, −0.25 to −0.04]) (Figure 3B). Those effects are attributable to reductions in primary dependence motive–subscale scores, in which the 0.4 mg/g (primary dependence motive score, −0.08 [95% CI, −0.11 to −0.05]) and 2.4 mg/g (primary dependence motive score, −0.06 [95% CI, −0.09 to −0.03]) doses produced steeper reductions than the 15.8 mg/g dose (primary dependence motive score, −0.001 [95% CI, −0.03 to 0.03]); no significant effects were seen on secondary dependence motive subscale scores (eFigure 2 in Supplement 2).

### Biochemical Markers

**Figure 4.**
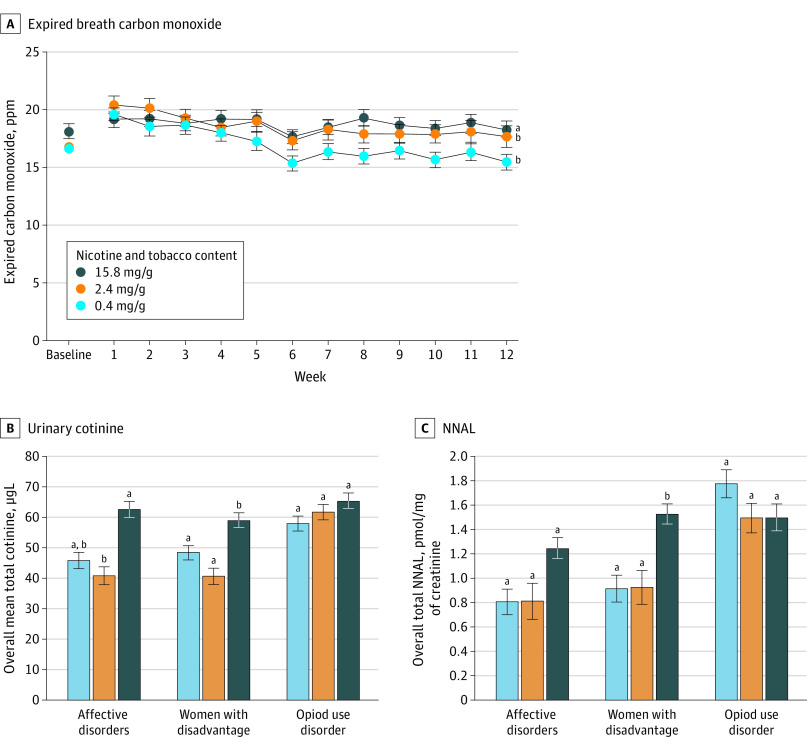
Biomarkers of Exposure as a Function of Dose, Time, and Population A, Data points are arithmetic means across participants and populations and error bars indicate SEM. Doses not sharing a superscript letter differ in linear trends across the 12-week study period. B, Data points are geometric means across participants and time at each dose; error bars represent SEM. Data points not sharing a superscript letter differed significantly in post-hoc testing. C, Data points are geometric means across participants and time at each dose; error bars indicate SEM. Data points not sharing a superscript letter differed significantly in post hoc testing.

Effects on breath CO varied by dose and time across populations (Cohen *d* = 0.09; *P* < .001) (Figure 4A), with steeper decreasing linear trends across weeks at 0.4 mg/g (weekly change, −0.31 [95% CI, −0.40 to −0.23] ppm) and 2.4 mg/g (weekly change, −0.25 [95% CI, −0.34 to −0.16] ppm) doses compared with the 15.8 mg/g dose (weekly change, −0.05 [95% CI, −0.13 to −0.03] ppm).

Effects of dose interacted with population for urine cotinine (Cohen *d* = 0.22; *P* = .004) and NNAL (Cohen *d* = 0.22; *P* = .005) (Figure 4B and C). The 0.4 mg/g and 2.4 mg/g doses decreased cotinine and NNAL levels more than the 15.8 mg/g dose among women with disadvantage; a similar pattern was seen among smokers with affective disorders; although for cotinine, only the difference between 2.4 mg/g and 15.8 mg/g was significant; dose failed to change either measure among smokers with OUD after Bonferroni correction.

### Simulated Modeling of Consumer Demand: Cigarette Purchase Task

Demand amplitude (ie, amount smoked if cigarettes were free) for study cigarettes varied by dose and time (Cohen *d* = 0.22; *P* < .001) (eFigure 3 in Supplement 2). The 0.4 mg/g dose had lower demand amplitude than the 15.8 mg/g dose across assessments and populations, and the 2.4 mg/g dose did so at the 6- and 12-week assessments. The VLNC doses did not differ from each other. Effects on demand persistence (ie, continuing to smoke despite increasing costs) varied only by dose (Cohen *d* = 0.16; *P* = .003), eFigure 3 in Supplement 2), with the 0.4 mg/g dose, but not the 2.4 mg/g dose, having lower demand persistence than the 15.8 mg/g dose.

Effects on demand amplitude for usual-brand cigarettes varied by dose and time across populations (Cohen *d* = 0.27; *P* < .001) (eFigure 3 in Supplement 2). No dose differences were discernible at week 2. The 0.4 mg/g and 2.4 mg/g doses decreased demand more than the 15.8 mg/g dose at weeks 6 and 12, with no difference between VLNC doses (eFigure 3 in Supplement 2). There were no dose effects on demand persistence (eFigure 3 in Supplement 2).

### Withdrawal and Craving

There were no effects of dose on Minnesota Tobacco Withdrawal Scale total scores (eTable 4 in Supplement 2). Effects on QSU Factor 1 (ie, desire/intention to smoke) scores differed by dose, time, and population (Cohen *d* = 0.10; *P* = .02), with at least 1 VLNC dose differing from the 15.8 mg/g dose among smokers with affective disorders and women with disadvantage, but not smokers with OUD (eFigure 4 in Supplement 2). Effects on QSU Factor 2 (ie, anticipated relief from negative affect) varied by dose and time (Cohen *d* = 0.12; *P* < .001) (eFigure 5 in Supplement 2), with decreasing linear trends across weeks at 0.4 mg/g (−0.02 [95% CI, −0.03 to −0.01]) and 2.4 mg/g (−0.01 [95% CI, −0.02 to −0.01]) doses and an increasing trend at the 15.8 mg/g dose (0.02 [95% CI, 0.01 to 0.03]).

### Smoking Topography

No significant effects of dose were noted on smoking topography measures suggestive of compensatory smoking (ie, adjustments in smoking topography to sustain a desired nicotine blood level). Dose did not significantly alter total puff volume or the magnitude of changes in breath CO from before to after smoking (eTable 5 in Supplement 2).

### Abstinence Outcomes

Mean number of days without smoking during the study varied by dose, with those in the 0.4 mg/g condition (3.5 [95% CI, 2.1 to 4.8] days) and those in the 2.4 mg/g condition (1.3 [95% CI, 0.6 to 2.1] days) abstaining on more days than those in the 15.8 mg/g condition (0.5 [95% CI, 0.3, to 0.7] days) (0.4 mg/g vs 15.8 mg/g: Cohen *d* = 0.96; *P* < .001; 2.4 mg/g vs 15.8 mg/g: Cohen *d* = 0.31; *P* < .001). Similarly, larger proportions of participants assigned to the 0.4 mg/g (18 individuals [6.6%]) and 2.4 mg/g conditions (10 individuals [4.4%]) than to the 15.8 mg/g condition (3 individuals [1.2%]) reported a quit attempt during the study (0.4 mg/g vs 15.8 mg/g: OR, 6.00 [95%CI, 1.74 to 20.73]; 2.4 mg/g vs 15.8 mg/g: OR, 3.95 [95% CI, 1.07 to 14.60]). Dose had no effect on proportion of participants abstinent for 24 hours following week 12 assessments (0.4 mg/g: 104 individuals [46.2%]; 2.4 mg/g: 82 individuals [43.2%]; 15.8 mg/g: 102 individuals [46.8%]; *P* = .74) or who reported trying to quit during 30-day follow-up (0.4 mg/g: 31 individuals [20.3%]; 2.4 mg/g: 35 individuals [28.5%]; 15.8 mg/g: 39 individuals [23.9%]; *P* = .27).

### Adverse Events, Health and Safety Status

Most participants (670 participants [86.5%]) reported at least 1 AE; incidence did not differ by dose (eTable 6 and eTable 7 in Supplement 2). The most commonly reported AEs were infections (262 reports), respiratory problems (234 reports), and psychiatric problems (228 reports). A total of 30 Food and Drug Administration–defined serious AEs occurred, and incidence did not differ by dose (eTable 8 and eTable 9 in Supplement 2). Of these, 3 serious AEs were deemed related to study participation: 1 each for depression and suicidal ideation, chronic pain, and psychosis. All serious AEs were in the 15.8 mg/g group, and all individuals recovered without residual effects. Another 85 individuals had an AE rated as severe intensity, with 10 AEs deemed study-related. There were no statistically significant differences by dose in scores on the respiratory health questionnaire^10^ and Overall Anxiety Severity and Impairment Scale.^52^ Mean Beck Depression Inventory^53^ scores varied by dose (Cohen *d* = 0.06; *P* = .02) (eFigure 5 in Supplement 2), with higher scores at the 0.4 mg/g dose (10.7 [95% CI, 10.2 to 11.3]) than at the 15.8 mg/g dose (9.8 [95% CI, 9.3 to 10.4]), although within the minimal-depression range (ie, 0-13).^54^ Scores reflecting severe depression (ie, >29) did not differ by dose. Neither past-30-day alcohol use, binge drinking, illicit drug use, nor drug toxicology screens differed by dose.

## Discussion

The results of these 3 randomized clinical trials replicate 2017 findings^19^ that acute exposure to VLNCs decreases the reinforcing value of smoking in these at-risk populations and extends them by demonstrating that chronic exposure decreases smoking rate, nicotine dependence severity, and toxin exposure compared with cigarettes with a nicotine content level comparable to commercial cigarettes. Few differences were observed between the 0.4 mg/g and 2.4 mg/g cigarettes, and both differed from 15.8 mg/g cigarettes, consistent with results in the general population of smokers.^10^ Logically, reducing nicotine levels as low as possible seems safest regarding addiction risk, but this study offers little evidence differentiating the 0.4 mg/g and 2.4 mg/g doses. Importantly, neither VLNC dose produced untoward withdrawal or craving, compensatory smoking, or other serious AEs. Mild mood disturbance was noted with the 0.4 mg/g dose, which could be due to overlap between some Beck Depression Inventory symptoms with nicotine withdrawal.

The effects of VLNC cigarettes in this study are generally consistent with those seen in comparable randomized clinical trials in the general population of adult daily smokers, with assignment to 0.4 mg/g resulting in approximately 20% to 30% reductions in CPD, accompanied by reductions in dependence severity and toxin exposure.^10,11^ That these effects occurred in at-risk smokers not currently trying to quit smoking is encouraging.^1,2,3,4,5,6,15,16,17,18,19^ When providing free cigarettes to established smokers, smoking rate increases, as seen at the 15.8 mg/g dose, are expected.^10^ Comparable increases did not occur with the VLNCs. The most important comparisons in this parallel-groups design are the differences among dose conditions in total CPD at week 12.

Several trials with daily smokers have reported effects of VLNCs on abstinence outcomes. Donny et al^10^ reported that quit attempts after the study were greater among smokers assigned to 0.4 mg/g compared with those assigned to 15.8 mg/g (35% vs 17%). Hatsukami et al^11^ did not report poststudy quit attempts but noted increases in mean number of cigarette-free days in participants assigned to 0.4 mg/g compared with those assigned to 15.8 mg/g (10.1 days vs 3.1 days). Tidey et al^18^ reported no significant effects on abstinence in smokers with serious mental illness. We observed effects similar to Hatsukami et al,^11^ with more cigarette-free days and quit attempts with VLNCs. Again, these effects are notable in that this study and most prior VLNC studies excluded individuals currently interested in quitting and thus are likely weak tests of their ability to prompt abstinence. In the only study in smokers interested in quitting,^7^ to our knowledge, VLNC pretreatment increased cessation rates.

Regarding population differences, none were noted on the primary outcome (ie, week 12 total CPD) nor several important secondary outcomes (ie, mean total or study CPD across study weeks, dependence severity scores, and breath CO). The reductions in breath CO biochemically confirm the self-reported reductions in total cigarette smoking across populations, the most lethal form of tobacco use. Population differences were noted on total urine cotinine and NNAL levels. Those differences involved relatively blunted dose differences among smokers with OUD, likely attributable to them using more noncombustibles even at the 15.8 mg/g dose. The goal of reducing nicotine content in cigarettes to very low levels is to eventually eliminate all use of combusted cigarettes.^55^ Ideally, current smokers would eventually quit, but some may be unable or unwilling to totally quit nicotine use.^55,56^ Individuals with OUD have low rates of quitting, even compared with other at-risk populations, and thus may be overrepresented among those unable or unwilling to quit nicotine.^57,58,59^ Migrating these individuals to exclusive use of noncombusted sources of nicotine may be a more achievable harm-reduction goal than complete nicotine abstinence.^57,58,59^

### Limitations

This study has some limitations, many of which are consistent with those in prior trials on VLNCs.^10,11,12^ Most notably, the study participants were paid volunteers not selected to be representative of the US population, which could limit generalizability. Additionally, use of nonstudy cigarettes and other sources of nicotine were observed across populations, especially in the initial weeks, which likely attenuated the precision of estimates of dose differences between study cigarettes.

## Conclusions

The results of these 3 randomized clinical trials on extended exposure in combination with earlier results on acute exposure^19^ provide compelling evidence that capping maximal nicotine content of cigarettes at very low levels^55,56,60^ has the potential to reduce their addiction potential in populations at high risk of smoking, addiction, and smoking-related adverse health consequences. Reducing smoking in these high-risk populations is an important U.S. population health priority.^2,15,19,55,56^

## Notes

Supplement 1. **Trial Protocol**

Supplement 2. **eFigure 1.** Nonstudy Cigarettes Per Day **eFigure 2.** Wisconsin Index of Smoking Dependence and Motivation **eFigure 3.** Effects of Dose and Time on Amplitude and Persistence of Demand From the Cigarette Purchase Task **eFigure 4.** Questionnaire of Smoking Urges: Factor 1 **eFigure 5.** Questionnaire of Smoking Urges: Factor 2 **eFigure 6.** Beck Depression Inventory Score by Dose **eTable 1.** Demographic and Smoking Characteristics by Completor Status **eTable 2.** Participant Inclusion Criteria by Study Population **eTable 3.** Participant Exclusion Criteria by Study Population **eTable 4.** Minnesota Tobacco Withdrawal Scale Total Score by Dose **eTable 5.** Smoking Topography and Before and After Smoking Carbon Monoxide Changes **eTable 6.** Total Number of Adverse Events **eTable 7.** Number of Participants with Any Adverse Events **eTable 8.** Count of Serious and Severe Adverse Events **eTable 9.** Description of Serious and Severe Adverse Events

Supplement 3. **Data Sharing Statement**
