# Supplementary material for: Changes in Cigarette Consumption With Reduced Nicotine Content Cigarettes Among Smokers With Psychiatric Conditions or Socioeconomic Disadvantage: 3 Randomized Clinical Trials
Source: JAMA Netw Open. 2020 Oct 20;3(10):e2019311. doi: 10.1001/jamanetworkopen.2020.19311 (PMC7576411; doi:10.1001/jamanetworkopen.2020.19311)
Supplement: Supplement 1. — Trial Protocol [file jamanetwopen-e2019311-s001.pdf]

eSupplement 1

eSupplement 1 was prepared by the authors to provide interested readers additional protocol and statistical-analysis details on the three randomized, controlled trials reported in *Higgins ST, Tidey JW, Sigmon SC, et al.* Changes in cigarette consumption with reduced nicotine content cigarettes among smokers with psychiatric conditions or socioeconomic disadvantage: 3 randomized clinical trials.

Protocols for each of the three trials are detailed separately below followed by the Statistical Analysis Plan for those trials.

The protocol information provided below is what local Institutional Review Boards at each research site approved.

Summary of Protocol Modifications:

Randomization sequence had to be revised on May 10, 2017 and again on February 9, 2018 to accommodate a supplier (National Institute on Drug Abuse) shortage of two doses of research cigarettes (2.4 mg/g non-menthol and 15.8 mg/g menthol cigarettes). We over-assigned to the 0.4 mg/g non-menthol dose during this shortage and then to the two doses that were in short supply once they became available again. To accommodate any potential influence on outcomes, menthol cigarette use was included as a covariate in all analyses.

Changes in Cigarette Consumption with Reduced Nicotine Content Cigarettes Among Smokers with Psychiatric Conditions or Socioeconomic Disadvantage: Three Randomized Clinical Trials

Stephen T. Higgins, PhD[1], Jennifer W. Tidey, PhD[2], Stacey C. Sigmon, PhD[1], Sarah H. Heil, PhD[1], Diann E. Gaalema, PhD[1], Dustin Lee, PhD[3], John R. Hughes, MD[1], Andrea C. Villanti, PhD[1], Janice Y. Bunn, PhD[1], Danielle R. Davis, PhD[1,3], Cecilia L. Bergeria, PhD[1,4], Joanna M. Streck, BA[1,5], Maria Parker, PhD[1,6], Mollie E. Miller, PhD[2], Michael DeSarno, MS[1], Jeff S. Priest, PhD[1], Patricia Cioe, PhD[2], Douglas MacLeod, MS[1], Anthony Barrows, BA[1], Catherine Markesich, BA[1], Roxanne Harfmann, BA[1]

[1]UVM Tobacco Center of Regulatory Science, University of Vermont, Burlington, VT, [2]Center for Alcohol and Addiction Studies, Brown University, Providence, RI, [3]Yale University Tobacco Center of Regulatory Science, Yale University School of Medicine, New Haven, CT, [4]Behavioral Pharmacology Research Unit, Johns Hopkins University School of Medicine, Baltimore, MD, [5]Department of Psychiatry, Massachusetts General Hospital, Harvard Medical School, Boston, MA, [6]School of Public Health, Indiana University, Bloomington, IN

eSupplement 1

Table of Contents

Content	Page Number
eProtocol: Smokers with Affective Disorders:……………………………………………….……4	
eProtocol: Smokers with Opioid Use Disorder:……………………………………………..…… 45	
eProtocol: Smokers with Socioeconomic Disadvantage (women of reproductive age):….85	
Statistical Analysis Plan……………………………………………………………...………123	

STUDY PROTOCOL: SMOKERS WITH AFFECTIVE DISORDERS

Table of Contents

Objective: 8
Background Information 8
Potential Benefits and Unintended Consequences of Nicotine Reduction Policy for Smokers with Mood and Anxiety Disorders: 8
Cigarettes to Be Assessed in This Study 10
Screening Procedures 10
______Recruitment 10
______Informed Consent Process 11
______Screening Measures/Assesments and Physiological Samples to be Collected 11
______Suicidality/Mental Health Monitoring 13
______Includion/Exclusion Criters 13
______Eligibility Determination 15
Baseline Procedures 16
______Measures/Assesments and Physiological Samples to be Collected 16
______Cognitive Tasks 13
______Smoking Topography 19
______Interactive Voice Response 19
______Description of Biological Specimens 19
______Biomarker Shipping and Storage 20
______fMRI Testing 20
Experimental Procedures 20
______Experimental Period 21
______Visit Scheduling Requirements 21
______Experimental Visits Weeks 1, 3, 5, 7, 9 and 11 Procedures 13
_________Measures/Assessments 13
______Experimental Visits Weeks 2, 4, 6, 8, 10 and 12 Procedures 13
_________Measures/Assessments 13
__________Week 12 fMRI Testing 13
______Interactive Voice Response System 13
______Variable Incentive Program 13
______Product and Procesures Compliance Review Sessions 13
______Quit Attempts During the Study Protocol 13
_________If a Participant is Currently Abstaining from Smoking with the Intention to Quit 13
_________If a Participant is Planning to Quit Smoking, But Has Not Initiated Quit Attempt 13

______Abstinence Assessment Session 13
_________Participants Who Meet Criteria for Abstinence 13
____________Measures/Assessments 13
____________Additional Tasks 13
_________Participants Who Do Not Meet Criteria for Abstinence 13
____________Measures/Assessments 13
______Participant Compensation 13
______End of Study 13
______30 Day Follow up Phone Call 13
Randomization 13
______Product Accountability 30
Statistical Methods and Sample Size 31
Potential Risks of Participation 31
______Risks of Participation 31
______Avoiding Risk to the Fetus 33
______Expected Benefits of Participation 33
______Study Debriefing 33
Protection Against Risk 13
______Data Collection Protections 33
______Data Storge 34
Adverse Events 34
Withdrawal or Monitoring of Participants 35
Data Safety Monitoring Board 36
Investigational Tobacco Product 37
Certificate of Confidentiality 37
Outcome Variables 38
References 39

Abbreviations

- VLNC: Very low nicotine content
- RNC: Reduced nicotine content
- NNC: Normal nicotine content
- CPD: Cigarettes per day
- CO: Carbon monoxide
- BAL: Breath alcohol levels
- BP: Blood pressure
- HR: Heart rate
- BPM: Beats per minute
- NMR: Nicotine metabolite ratio
- NNN: *N′*-nitrosonornicotine
- NNAL: 4-(methylnitrosamino)-1-(3-pyridyl)-1-butanol
- BDI: Beck's Depression Inventory
- FTND: Fagerström Test for Nicotine Dependence
- WISDM: Wisconsin Index of Smoking Dependence Motives
- TLFB: Timeline Follow Back
- MNWS: Minnesota Nicotine Withdrawal Scale
- QSU: Questionnaire of Smoking Urges
- CES: Cigarette Evaluation Scale
- CPT: Continuous Performance Task
- IVR: Interactive Voice Response
- EDC: Electronic Data Capture
- CPT: Cigarette Purchase Task
- FSPTCA: Family Smoking Prevention and Tobacco Control Act
- MDD: Major Depressive Disorder
- BMI: Body Mass Index
- PHQ: Patient Health Questionnaire
- GAD: Generalized Anxiety Disorder
- OASIS: Overall Anxiety Severity and Impairment Scale
- MINI: Mini International Neuropsychiatric Interview
- PSS: Perceived Stress Scale

- PANAS: Positive and Negative Affect Schedule
- BRIEF-A: Behavioral Rating Inventory of Executive Function
- EQ-5D: Euro-Qol
- TPQ: Time Perspectives Questionnaire
- D-KEFS: Delis-Kaplan Executive Function System
- DDT: Delay Discounting Task
- WASI-II: Wechsler Abbreviated Scale of Intelligence-II
- SST: Stop Signal Task
- FeNO: Fractional Exhaled Nitric Oxide
- 3HC: 3-hydroxycotinine
- COT: Cotinine

Protocol

Objective:

The primary overall objective of this study is to evaluate the effects of extended exposure to cigarettes differing in nicotine content in smokers with mood and anxiety disorders using a 3-condition, parallel groups design. After a baseline period in which daily smoking rate and other baseline assessments are completed, participants will be randomly assigned to one of three cigarette conditions (nicotine content averaged across menthol and non-menthol cigarettes: 0.04, 2.4, and 15.8 mg nicotine/gram of tobacco) for the 12-week experimental period.

Background Information:

The 2009 Family Smoking Prevention and Tobacco Control Act (FSPTCA) gave the Food and Drug Administration (FDA) regulatory authority over tobacco products, including nicotine levels in cigarettes. That is an exciting development as it creates the opportunity to examine the Benowitz and Henningfield (1994) hypothesis that smoking prevalence, nicotine dependence, and smoking-related morbidity and mortality can be lowered substantially by reducing the nicotine content of cigarettes to non-addictive levels. Computer modeling predicts that reducing nicotine levels in cigarettes would produce substantial improvements in population health (Tengs et al., 2005). An essential initial step towards the implementation of such a policy is to thoroughly investigate its safety and potential unintended adverse consequences in a variety of smoker subpopulations. Indeed, the FDA's Center for Tobacco Products seeks to establish research centers to assist with the mission of investigating such regulatory matters related to the FSPTCA (see RFA-DA-13-003). The FDA explicitly notes that researching tobacco regulatory questions in vulnerable populations is a crosscutting agency priority, listing adult smokers with psychiatric disorders among the vulnerable populations of interest.

Potential Benefits of a Nicotine Reduction Policy for Smokers with Mood and Anxiety Disorders:

The prevalence of smoking among people with mood and anxiety disorders is twice that of the general population (Lasser et al., 2000). In addition, smokers with mood and anxiety disorders have increased markers of tobacco dependence severity (smoke more cigarettes per day, report more severe nicotine dependence) than smokers without mental illness (Breslau, Novak, & Kessler, 2004; Dierker, & Donny, 2008; Goodwin, Zvolensky, Keyes, & Hasin, 2012; Grant, Hasin, Chou, Stinson, & Dawson, 2004; Lawrence, Considine, Mitrou, & Zubrick, 2010). Although many smokers with mood and anxiety disorders are able to achieve abstinence with integrated pharmacological and behavioral smoking cessation treatments (e.g., Hall et al., 2006; McFall et al., 2006), these smokers infrequently access adequate smoking treatments, and smoking cessation rates are very low among the vast majority of smokers with mood and anxiety disorders who do not enter formal smoking-cessation treatments (Prochaska et al., 2004; Hall & Prochaska, 2009; Ziedonis et al., 2008).

Potential Unintended Consequences of a Nicotine Reduction Policy for Smokers with Mood or Anxiety Disorders:

Given that approximately 30% of smokers have mood or anxiety disorders (Goodwin et al., 2012; Grant et al., 2004), a nicotine reduction policy has the potential to dramatically improve

population health. However, it is important to determine whether a nicotine reduction strategy could have unintended negative consequences for these smokers. For example, smokers with mood and anxiety disorders may experience more severe mood symptoms or craving when smoking very low nicotine content cigarettes, and may respond by increasing their smoking rate or puff topography characteristics in attempts to compensate for the effects of nicotine reduction. One potential predictor of their response to a reduction policy is their response to smoking-cessation interventions. Abstinence during smoking treatment has inconsistent effects on mood in smokers with depression and anxiety disorders (Breslau, Kilbey, & Andreski, 1992; Hall et al., 2006; McFall et al., 2006; Blalock et al., 2008; Smith, Homish, Giovino, & Kozlowski, 2014; Taylor et al., 2014; Tsoh et al., 2000; Weinberger, Desai, & McKee, 2010). Moreover, as treatment studies do not experimentally control abstinence, causality in these relationships cannot be determined. Few human laboratory studies have examined whether nicotine increases depression and anxiety symptoms among smokers with these disorders. The most relevant study in smokers with major depressive disorder (MDD) examined the effects of abstinence in 38 smokers with current MDD who were randomized to receiving either 21 mg nicotine or placebo patches for 2 weeks (Thorsteinsson et al., 2001). Interpretation of the results is complicated by the fact that 14 participants (50% of those in the placebo condition, 22% of those in the nicotine condition) did not complete the study due to resumption of smoking. Among completers, nicotine withdrawal symptoms were found to be somewhat (but not significantly) higher in the placebo group, and depression symptoms and negative mood ratings decreased (i.e., mood improved) over time across patch conditions (Thorsteinsson et al., 2001). Withdrawal symptoms were higher among a subset of non-completers for whom these data were available than among study completers. Thus, although nicotine abstinence did not exacerbate depression severity among study completers, those most affected by abstinence dropped out of the study. A recent laboratory study evaluated the effects of overnight abstinence and smoking re-initiation on nicotine withdrawal symptoms (Dedert et al., 2012). Compared to smokers without PTSD, those with PTSD displayed higher craving and withdrawal symptoms following overnight abstinence. After re-initiating smoking, those with PTSD reported less relief from craving and negative affect compared to smokers without PTSD. In both groups, smoking a nicotine cigarette after abstinence was associated with a greater reduction in craving and withdrawal symptoms than smoking a denicotinized cigarette. Thus, results of both studies indicate that brief nicotine abstinence is associated with increased mood disruption during smoking abstinence in smokers with MDD or PTSD. To our knowledge, no laboratory studies have evaluated the effects of extended nicotine abstinence on mood in smokers with current mood or anxiety disorders.

Another mechanism that may underlie the high prevalence of smoking among people with mood disorders is nicotine's enhancement of cognitive functioning. MDD is associated with neurocognitive impairments, including deficits in executive functioning, attention and working memory (Castaneda et al., 2008). Smoking abstinence degrades, and nicotine improves, cognitive performance in smokers in general (Heishman et al., 2010) by increasing the release of acetylcholine, dopamine, glutamate, and other neurotransmitters mediating cognition (Di Matteo et al., 2007; Poorthuis et al., 2009; Levin et al., 2006). Given that enhancement of cognitive performance may help to maintain smoking in people with MDD (Malpass & Higgs,

2007), nicotine reduction could exacerbate their cognitive dysfunction, and smokers with MDD could increase their smoking rates or smoke inhalation patterns in attempts to overcome these effects.

Cigarettes to be assessed in this study:
The cigarettes to be used in this study were made under an NIH contract with production being overseen by the Research Triangle Institute (referred to as “Spectrum cigarettes”). NIH currently has approximately 10 million of these cigarettes (of varying types) for research purposes. The cigarettes selected for the study span the range of yields likely to produce the hypothesized effects, as described above. Spectrum cigarettes are not currently commercially available, although they are similar in many ways to marketed cigarettes (e.g., similar manufacturing, filter, paper, etc.).

Screening Procedures
Recruitment

A sample size of 207 completers is proposed to test the primary outcome. Anticipating 25% attrition, and six pilot participants (3 at Brown, 3 at UVM), 282 participants will be enrolled across both sites (94 at Brown, 188 at UVM). Potential participants will respond to community advertisements (local newspapers, community bulletin boards, lab Facebook page, Facebook ads, lab website, center website, behavioral health centers, Craigslist, city buses, etc.) that contain a study description, link to an online survey and the name and phone number of the Research Assistant. Participants can choose to complete the pre-screening questionnaire online or by phone. The Patient Health Questionnaire-4 (Kroenke et al., 2009) will be used to screen for probable mood or anxiety disorder. This 4-item instrument, which comprises the first 2 items of the Patient Health Questionnaire 9-item (PHQ-9) scale (Kroenke et al., 2001) and the first 2 items of the Generalized Anxiety Disorder 7-item (GAD-7) scale (Spitzer et al., 2006), measures the two core DSM-IV criteria for major depressive disorder and generalized anxiety disorder, respectively. The PHQ–4 begins with the stem question: “Over the last 2 weeks, how often have you been bothered by the following problems?” and each item is scored from 0 (“not at all”), 1 (“several days”), 2 (“more than half the days”), or 3 (“nearly every day”). Therefore, the total score on this composite measure ranges from 0 to 12. A total score of 3 for the 2 anxiety items or the 2 depression items, plus questions querying past treatment for depression or anxiety, will be used to identify probable cases. If deemed eligible, those who complete the online questionnaire will be called by the Research Assistant to further discuss the study. The RA will read a script briefly explaining the study. Participants will be informed that this is not a smoking cessation program, and that smoking cessation services are available in the community independent of their decision to participate in this study. If interested, they will be scheduled for an in-person screening interview. Those who call into the laboratory will be read a script briefly explaining the study. After verbal informed consent is received, the participants will be asked questions over the phone to determine initial eligibility. Callers will be informed that this is not a smoking cessation program, and that smoking cessation services are available in the community independent of their decision to participate in this study. Eligible and interested participants will be scheduled for an in-person screening interview.

Potential participants will be instructed to bring a pack of their usual brand cigarettes, all prescription medications they are currently taking and identification (example, driver's license) to the screening visit. If participants anticipate not having acceptable ID, site staff should consult with the project coordinator or study PI.

A participant must complete his/her in-person screening session within 30 days of completing the pre-screening questionnaire. If the participant is not able to attend the in-person screening visit in that timeframe, he/she will need to complete the pre-screening questionnaire again.

Informed Consent Process:
Before beginning the informed consent process, potential participants will need to produce identification as described above. The interviewer will confirm the age and identity of the participant. If the participant is not between the ages of 18 and 70, he/she will be dismissed without payment. During the in-person screening session, study information will be presented and written informed consent will be required prior to participating in the screening session. In order to ensure adequate informed consent, participants will be asked to read the first several lines aloud (to determine literacy) and will then be given ample time to read the consent document. If the interviewer suspects the participant is not literate, he or she will have them continue reading further to confirm. Inability to read and comprehend written study materials will result in ineligibility and the interviewer will inform the participant that they are not eligible. Only after the participant and the researcher are fully satisfied that the participant understands the purpose of the study, the confidentiality of the data, the procedures, the risks/benefits and his/her rights as a research participant will the consent form be signed and the participant undergo screening procedures.

Screening Measures
Those who consent will be screened for eligibility using the following measures:

The following physiological measures will be collected, recorded on paper, and entered into REDCap by the interviewer at the end of the visit:

1) Breath alcohol levels (BAL) will be measured using an Alcosensor monitor. Participants with levels over 0.01 g/l may reschedule the interview but will need to be re-consented to ensure they have received adequate informed consent. They will be excluded if they are positive the second time.
2) Weight and height will be measured to determine the participant's Body Mass Index. Weight will be measured in kilograms and height will be measured in centimeters.
3) Expired breath carbon monoxide (CO) levels will be assessed using a Smokerlyzer ED50 CO meter (Bedfont Instruments), a reliable and valid measure of recent smoking.
    a. NicAlert Strips will be used to asses urinary cotinine levels if a participant's carbon monoxide reading is less than or equal to 8 ppm.
4) A urine toxicological screen will be performed to assess the presence of illicit drugs including marijuana, cocaine, opiates, oxycodone, benzodiazepines, barbiturates, amphetamines, methadone, buprenorphine, methamphetamines, MDMA and PCP. Participants who fail the drug screen for drugs other than marijuana may reschedule the interview but will need to be re-consented to ensure they have received adequate informed consent. They will be excluded if they are positive for drugs other than marijuana the second time.
5) Urine Pregnancy Test (HCG detection) will be performed for all participants.

6) Blood pressure and heart rate will be measured using a CritiCare monitor to help the licensed medical professional determine final participant eligibility.

The following screening questionnaires will be participant-administered via paper and then will be entered into REDCap by the interviewer at the end of the visit:

1) Identifying Information Form will include the participant's REDCap Subject Identifier, name, address (including the county of residence), email address, phone number, age, date of birth, and social security number (if applicable).
    a. This form will be entered into the 'Identifying Information Access Database'.
        i. Each site will have a separate 'Identifying Information Access Database'.
        ii. Identifying information will not be shared with other sites. Each site is responsible for maintaining confidentiality of this information.
        iii. Identifying information will be kept in a locked file cabinet (source document) and in a password protected Access Database (electronic version) separate from all other study data.
2) Beck Depression Inventory (BDI; Beck, Ward, & Mendelson, 1961), to assess depressive symptoms.
3) Overall Anxiety Severity and Impairment Scale (OASIS; Norman et al., 2006) to assess frequency and severity of anxiety symptoms.

The following screening assessments will be administered as an interview and then will be entered into REDCap by the interviewer at the end of the visit:

1) The Mini International Neuropsychiatric Interview (MINI) suicide subscale (Sheehan et al., 1997) to evaluate suicide risk
2) The Mini International Neuropsychiatric Interview (MINI) PLUS 6.0 Modules
3) MINI Follow-up Questionnaire (if applicable)
4) Tobacco Use History and Exposure Questionnaire, which measures variables such as smoking amount, cigarette brand, age of initiation of smoking, number of quit attempts, duration of quit attempts and duration of smoking
5) Smoking Cessation Therapy Use Questionnaire
6) Time Since Last Cigarette Questionnaire
7) Medical History Questionnaire to assess current diagnoses, symptoms and past health problems
    a. The medications section will be transferred onto the 'Concomitant Medications' form and entered into REDCap

The following screening assessments will be completed by the participant directly in REDCap, except where noted:

1) Demographic History Questionnaire, which will assess age, gender, ethnicity, race, education, income, marital status, and employment history
2) Alcohol Use Questionnaire (12 month and 1 month version)
3) Drug Use Questionnaire (12 month and 1 month version)
4) Fagerström Test for Nicotine Dependence (FTND; Heatherton et al., 1991)
5) Wisconsin Inventory of Smoking Dependence Motives-Brief Scale (WISDM; Piper et al., 2008), will be administered to assess nicotine dependence severity

6) Smoking Stages of Change Algorithm as well as a contemplation ladder to assess intention to quit smoking (DiClemente et al., 1991).
7) The Mini International Neuropsychiatric Interview (MINI 6.0) (Sheehan et al., 1990) a structured diagnostic interview to evaluate psychiatric disorders
    a. Will be completed by participant through the In-Home Screening system supported by Medical Outcomes Systems

In the event that the REDCap website is not functioning, the assessments will be printed out and administered on paper. The source documents will be kept in the participant's binder. The interviewer will enter the data into REDCap when it resumes functioning properly. This information should be recorded in the 'End of Visit Evaluation Form' and filed in the participant's binder.

Suicidality/Mental Health Monitoring

Participants who endorse suicidal intention in the past month or a suicide attempt in the past 6 months as indicated on the BDI (score > 1 on question 9) or MINI suicide subscale (endorse question 4 and/or 5 on the MINI suicide subscale or question 6 on the MINI suicide subscale with suicide attempt in the past 6 months) or answer "yes" to question A3g on the MINI Neuropsychiatric interview and symptoms have occurred in the past two weeks, will not be eligible to participate in the study. The research staff member will contact a licensed on-site clinician for evaluation. In the event that no clinician is available, staff will put the participant in contact with the National Suicide Prevention Lifeline at 1-800-273-8255. They will also contact the Study Coordinator and Site PI to inform them of the situation as soon as possible. Additionally, they will contact the Project Coordinator to inform her of the situation. The participant will be paid $25 (+$25 bonus if applicable) and provided with local mental health resources. Post enrollment, any report of suicidal ideation or attempt by a participant will be grounds for immediate withdrawal from the study.

Inclusion/Exclusion Criteria

Inclusion Criteria:

1) Men and women ages 18-70,
2) Past-year: MDD, dysthymic disorder, generalized anxiety disorder, post-traumatic stress disorder, obsessive-compulsive disorder, phobia or panic disorder with or without agoraphobia, based on MINI structured interview, OR Lifetime diagnosis of one of the above based on MINI with a self-report of currently receiving treatment (prescribed psychoactive medication, behavioral therapy, etc.),
3) Report smoking ≥ 5 cigarettes per day for the past year,
4) Provide an intake breath CO sample >8 ppm, (if ≤ 8 ppm, then NicAlert Strip > 2)
5) Be without current substance abuse/dependence other than nicotine,
6) Be sufficiently literate to complete the research-related tasks,
7) Be in good physical health without serious illness or change in health in the past three months as determined by the licensed medical professional at each site,
8) Not pregnant or nursing and report using oral, implant, patch, ring, IUD, injection or barrier contraceptives or report being surgically sterile, or post-menopausal,
9) Report no significant use of other tobacco or nicotine products within the past month (more than 9 days in the past 30).

Exclusion Criteria:

1) Any prior regular use (used as primary cigarette outside of the laboratory) of Spectrum cigarettes (i.e., research cigarettes with reduced nicotine content),
2) Exclusive use of roll-your-own cigarettes,
3) Planning to quit smoking in the next 30 days,
4) A quit attempt in the past 30 days resulting in greater than 3 days of abstinence,
5) Positive toxicology screen for any of the following drugs: cocaine, opiates, methadone, oxycodone, buprenorphine, benzodiazepines, barbiturates, amphetamines, methamphetamines, MDMA and PCP
    a. Marijuana will be tested for but will not be an exclusionary criterion. Participants will be discouraged from smoking marijuana during the study.
    b. Participants with valid prescriptions for opiates, benzodiazepines, barbiturates, or amphetamines will not necessarily be excluded.
    c. Participants failing the toxicology screen will be allowed to re-screen once. These participants will need to be re-consented before being rescreened to ensure they have received adequate informed consent.
6) Breath alcohol level > 0.01
    a. Participants failing the breath alcohol screen will be allowed to re-screen once. These participants will need to be re-consented before being rescreened to ensure they have received adequate informed consent.
7) Self-report of binge drinking alcohol (more than 9 days in the past 30 days, 4/5 drinks in a 2 hour period in females/males),
8) Systolic blood pressure < 90 or ≥ 160 mmHg
    a. Participants failing for blood pressure will be allowed to re-screen once.
9) Diastolic blood pressure < 50 or ≥ 100 mmHg
    a. Participants failing for blood pressure will be allowed to re-screen once.
10) Breath CO > 80 ppm,
11) Heart rate is greater than or equal to 115 bpm or less than 45 bpm
    a. Participants failing for heart rate will be allowed to re-screen once.
12) Currently seeking treatment for smoking cessation,
13) Have used nicotine replacement, bupropion or other pharmacotherapies as cessation aids in the past month (bupropion will be allowed for treatment of depression),
14) Unstable psychiatric conditions (psychiatric medication changes in the past 4 weeks),
15) Current symptoms of psychosis, dementia or mania,
16) Suicidal ideation in the past month (score > 1 on the BDI question 9 or endorse question 4 and/or 5 on the MINI suicide subscale),
17) Answer “yes” to question A3g on the MINI Neuropsychiatric Interview Major Depressive Episode Module and symptoms occurred within the past two weeks,
18) Suicide attempt in the past 6 months (endorse question 6 on the MINI suicide subscale with suicide attempt in the past 6 months),
19) Participation in another research study in the past 30 days.
20) Co- habitation with any former research participant who was provided with Spectrum research cigarettes to smoke outside the lab.

Children under age 18 are excluded because they cannot legally buy cigarettes. Those with unstable medical, psychiatric, or medication conditions (as determined by the licensed medical professional) are excluded as these symptoms could affect a participant’s ability to complete the study. Examples include but are not limited to the following: angina, stroke, heart attack which occurred since phone screening, blood clots in the arms or legs for which the individual is undergoing active medical treatment, cancer requiring active chemotherapy or radiation therapy,

severe shortness of breath caused by conditions such as uncontrolled asthma, COPD, or arrhythmia, active untreated infection such as pneumonia, active untreated endocrine disorder such as hyperthyroidism. We will exclude those currently seeking smoking treatment and those who plan to quit in the next 30 days, as participation in this study may not lead to reductions in smoking. We will exclude pregnant or nursing women and women of reproductive potential who are unwilling to use acceptable forms of birth control throughout the study. We will also exclude anyone with current or recent alcohol or drug abuse problems as these factors could independently affect smoking behavior during the study. Individuals with baseline CO readings greater than 80 ppm, those with heart rate or blood pressure readings that are out of range (systolic: 90-159 mmHg; diastolic: 50-99 mmHg; HR: 45-114 bpm) and anyone who has attempted suicide in the past six months will be excluded from the study for safety concerns. Individuals who smoke ‘roll your own’ cigarettes exclusively will be excluded from the study because we will be unable to standardize their baseline smoking behavior. Individuals who have recently participated in a research study will be excluded as participation may have changed their smoking behavior, which may preclude a stable smoking baseline. Because participants are required to complete portions of the protocol independently, they will need to be able to independently read and comprehend the study materials.

Eligibility Determination:

The research assistant will review the entire screening assessment battery for initial eligibility determination, confirming the subject meets the above described inclusion/exclusion criteria. The final eligibility of the participant will be determined by a licensed medical professional (MD, DO, NP, PA, Master’s prepared RN or CRN) at each site after reviewing the Medical History Questionnaire, BDI, Mini Neuropsychiatric Interview, and the MINI suicide subscale. The licensed medical professional may meet with a participant if available and think it necessary for eligibility determination. He/she will sign off on eligibility prior to the first baseline visit. If the licensed medical professional determines the participant is not medically eligible to participate in the study, has current symptomatology that would interfere with interpretation of the data or is unlikely to complete the study he/she will inform the research assistants who will contact the participant prior to the first baseline visit. The licensed medical professional will not need to review the medical history forms of participants who are not eligible for other, non-medical reasons.

If a participant fails the urine toxicology screen due to a prescription medication he/she is taking, then he/she will not be automatically excluded. The interviewer will make note of this when he/she submits the forms to the licensed medical professional for final eligibility determination.

Once all the screening procedures have been completed, researchers will pay participants $25 (+$25 bonus if applicable) for their time as long as they pass the drug and breath alcohol tests and meet the minimum requirements for carbon monoxide or NicAlert levels. Those participants who do not pass these tests or meet these requirements will be dismissed from the study without payment. Marijuana will be tested for but will not be an exclusionary criterion. If a participant does not pass the drug test but has a current, valid prescription that would explain the failed test he/she will not be automatically excluded and will still receive the visit payment. Participants who meet all other eligibility criteria, sans the medical criteria, will be scheduled for the first baseline visit.

At the end of the screening session, the researcher will complete the End of Visit Evaluation Form, which will be filed in the subject's binder. This will allow the researcher to make note of any problems encountered during the visit, to track which computers were used for which tasks, and to assess the truthfulness of the participant in regards to self-report of tobacco use.

Baseline Procedures

This study will use a one-week, two-session baseline period to collect baseline individual difference measures and monitor daily usual-brand smoking behavior. At Baseline 1, participants will be provided their usual brand cigarettes to smoke, equivalent to 150% of their daily smoking rate. A time line follow back (TLFB) will be used to assess the daily cigarette use for the past 7 days. Participants will be provided their usual brand cigarettes for the first seven days of the baseline period. If the baseline period extends past seven days, participants will need to purchase their own usual brand cigarettes. Use of a two session baseline period will ensure stability of daily smoking reports, reduce reactivity to the daily cigarette monitoring, and reduce participant burden. During the two baseline sessions, participants will complete subjective questionnaires, assessments of cognitive functioning, and smoking topography. Each visit will last approximately two to four hours. At the end of each baseline session, the researcher will complete the End of Visit Evaluation Form, which will be filed in the participants' binder. This will allow the researcher to make note of any problems encountered during the visit, to track which computers were used for which tasks, and to assess the truthfulness of the participant in regards to self-report of tobacco use.

<u>Visit scheduling requirements for baseline period:</u>

Participants will be required to schedule the Baseline 1 visit within 30 days of their screening visit. If a participant still wants to be in the study after 30 days, he/she will need to be re-screened. The participant will need to be re-consented but will maintain the original REDCap Subject Identifier. The ideal target window separating Baseline 1 and Baseline 2 is between 7 and 12 days. The minimum is 7 days and the maximum is 21 days. If the participant does not complete the visit within 21 days, then he/she will not be rescheduled and will be discontinued from the study.

<u>Measures/Assessments</u>

Physiological measures collected at Baseline 1, recorded on paper, and entered into REDCap by the interviewer at the end of the visit:

1) BAL
2) Weight
3) CO
4) Blood Pressure
5) Heart Rate
6) Urine Toxicology

The following questionnaires will be participant-administered via paper at Baseline 1 and then will be entered into REDCap by the interviewer at the end of the visit:

1) BDI
2) OASIS

The following assessments will be administered as an interview at Baseline 1 and then entered into REDCap by the interviewer at the end of the visit:

1) Concomitant Medications Form
2) Health Changes Questionnaire, which will assess any weekly health changes,
3) Time Since Last Cigarette Questionnaire

The following assessments will be administered at Baseline 1 and completed by the participant directly in REDCap:

1) Perceived Health Risks Rating (Hatsukami et al., 2010), a measure of the perceived addictive potential and other health risks associated with cigarettes
2) Respiratory Health Questionnaire, a measure of cough, shortness of breath and other respiratory symptoms
3) Minnesota Nicotine Withdrawal Scale (MNWS; Hughes & Hatsukami, 1986), a measure of nicotine withdrawal
4) Questionnaire of Smoking Urges-brief scale - Usual Cigarette (QSU; Cox, Tiffany, & Christen, 2001; Tiffany & Drobes, 1991), which measures the urge to smoke
5) Cigarette Evaluation Scale – Usual Cigarette (CES; Westman, Levin, & Rose, 1992), which measures responses to cigarettes (e.g., reward, satisfaction).
6) Intolerance for Discomfort Questionnaire - (IDQ; Sirota et al., 2013), assesses intolerance for the discomfort of smoking abstinence. The measure includes three subscales: physical discomfort, emotional discomfort and smoking withdrawal discomfort.
7) Cigarette Purchase Task – Usual Brand Version (CPT; MacKillop et al., 2008), a self-report analogue of a progressive-ratio schedule that measures the relative reinforcing efficacy of cigarettes by querying how many of that day's cigarette they would consume in a day at varying prices. This task will indicate whether prolonged VLNC cigarette use reduces cigarette demand and increases sensitivity to increases in cigarette costs.
8) Perceived Stress Scale - 4 item (PSS-4; Cohen, Kamarck, & Mermelstein, 1983), which measures the degree to which life situations are appraised as stressful.
9) Positive and Negative Affect Schedule (PANAS; Watson, Clark, & Tellegan, 1988), which measures symptoms of positive and negative affect.

Physiological measures collected at Baseline 2, recorded on paper and entered into REDCap by the interviewer at the end of the visit:

1) BAL
2) Weight
3) CO
4) Blood Pressure
5) Heart Rate
6) Urine Toxicology
7) Urine Pregnancy

The following assessments will be administered as an interview at Baseline 2 and then entered into REDCap by the interviewer at the end of the visit:

1) Concomitant Medications Form
2) Health Changes Questionnaire
3) Time Since Last Cigarette Questionnaire

The following assessments will be administered at Baseline 2 and completed by the participant on paper and entered into REDCap by the interviewer at the end of the visit:

1) BDI
2) OASIS

The following assessments will be administered at Baseline 2 and completed by the participant directly in REDCap:

1) FTND
2) WISDM

In the event that the REDCap website is not functioning, the assessments will be printed out and administered on paper. The source documents will be kept in the participant's binder. The interviewer will enter the data into REDCap when it resumes functioning properly. This information should be recorded in the 'End of Visit Evaluation Form' and filed in the participant's binder.

Cognitive Tasks (Baseline 2 Only):

Cognitive functioning will be assessed using a battery of computer-based assessments. We will assess domains that are theoretically linked to smoking and likely to be sensitive to nicotine abstinence (Heishman, 1999; Kleykamp et al., 2005; Rycroft et al., 2006). Prior to test administration, participants will be trained to ensure their understanding of each test. Tests will be administered on a desktop computer.

1) **N-Back (0,2) Task** (Ernst et al., 2001): A measure of working memory in which participants view serially presented letters on a computer. They must indicate whether each letter presented is the same or different from the letter presented a specified number of positions back in the string of letters (e.g. 2-back).
2) **2-Letter Search** (Ernst et al., 2001): A measure of focused attention in which participants view strings of letters on a computer screen looking for whether each string contains or does not contain two target letters.
3) **Continuous Performance Test** (CPT; Myers et. Al., 2008): A measure of sustained attention, participants must monitor a string of stimuli (e.g. letters) serially presented on a computer screen monitoring for presentation of a target stimulus. The task is balanced so that they either must respond, or inhibit a response each time the target is presented.
4) **Stop Signal Task** (SST; Logan et al., 1984): A computer administered test of behavioral inhibition. Participants make frequent motor responses (e.g., left/right responses indicating if a visually presented arrow points left or right) and occasional, unpredictable response inhibitions (e.g., when a second arrow, pointing upwards, is presented). The stop signal delay (the interval between the onset of the go signal and stop signal) is

adjusted after each stop trial according to the participants' performance to achieve 50 percent inhibition success rate.

5) **Nicotine Stroop Task** (Stroop, 1935): Frequently used measure of inhibitory control functioning. It measures the ability to focus attention on relevant stimuli while ignoring distracters and to suppress a prepotent response (i.e., word reading) in favor of an atypical one (i.e., color naming). Participants will be shown a number of images. The images will either be nicotine related, evocative, or neutral in nature with different color borders (red, blue, green yellow). The participants will be asked to use response triggers to identify the color of the border for each picture as they appear on the screen.

Smoking Topography (Baseline 2 Only):

Puff Topography, a precise measure of smoking behavior (Brauer et al., 1996; Herning et al., 1981; Robinson & Forbes, 1975), will be used to examine whether prolonged use of the experimental cigarettes affects topography measures that may indicate smoking compensation (Strasser et al., 2007). Puff topography will be assessed using a CReSS pocket device that provides a valid measurement of puff number, puff volume, inter-puff interval and other indices (Blank et al., 2009). Carbon monoxide readings will be collected before and 15 minutes after puff topography. Participants will smoke one cigarette of their usual brand.

Interactive Voice Response System:

At the end of the first baseline visit, participants will be trained to use the Interactive Voice Response (IVR) System, which will contact participants each day throughout the study and ask about their smoking behavior as well as withdrawal symptoms the week before and after Baseline 2. We will also review the IVR adherence incentive program, which consists of $1 per call plus a $10 bonus for seven consecutive calls. Participants will be provided a study cell phone if they have unreliable telephone access, do not have enough monthly cell phone minutes or prefer not to use their own phone.

The IVR system is operated by TeleSage. To be enrolled in the IVR system, research staff will enter the participants initials, telephone number, subject identifier, and visit dates into the IVR TCORS website. Identifying information (initials and telephone numbers) will not be extracted with the data by the bioinformatics group. Please refer to TeleSage's privacy statement and HIPAA compliance form for additional information.

Baseline 2 biological specimens:

1) <u>Urine sample for smoking biomarker assessment</u>**:**
Participants will be asked to bring a urine sample (first void of the day) to the second baseline session for biomarker assessment. Samples will be stored at temperatures no more than -80°C. The tobacco-specific carcinogen biomarkers are total NNAL and PAH. Anatabine and anabasine will be tested in the VLNC condition to validate abstinence or measure the extent of nicotine replacement therapy being used. Total cotinine levels will also be assessed to measure daily nicotine exposure. Participant's will be reminded with a phone call the day before the visit, those who forget will be asked to provide an onsite urine sample.
2) <u>Pulmonary Marker:</u>
Fractional Exhaled Nitric Oxide (FeNO) will be assessed as a measure of lung function using the NIOX VERO, a hand-held device for exhaled NO analysis. FeNO involves no storing or shipping of specimens, rather, the participant will exhale slowly through the device to obtain the result, which will be recorded in the participant's source.

3) <u>Cardiovascular Markers:</u>
Blood samples will be used for measurement of a battery of cardiovascular biomarkers primarily focusing on three areas: glucose tolerance (fasting insulin, glucose, hemoglobin A1C), clotting markers (thrombin, fibrinogen, PAI-1), inflammatory markers (C-reactive protein, interleukin-6, D-Dimer). Secondary measures include: Fasting lipid profile (total cholesterol, triglycerides, HDL-C, LDL-C). Participants will be required to fast for a minimum of 8 hours. Ideally, participants will not eat or drink after midnight and blood draws will be done in the morning. After the blood draw, participants will be provided with a meal voucher so that they may eat before performing the remaining visit tasks. The following volumes and tubes will be collected: Two 5 mL SST tubes, one 10 mL EDTA tube and two 2.7 mL citrate tubes.

4) <u>Additional Blood Samples</u>:
Blood samples will also be used for assessing individual differences in nicotine metabolism by phenotyping (i.e., Nicotine Metabolic Ratio, NMR, which is phenotypically estimated as the ratio of 3-hydroxycotinine [3 HC] to cotinine [COT] in plasma). One 10 mL EDTA tube will be collected.

We will store blood for the purposes of analyzing additional cardiovascular biomarkers or genotyping of individual differences in nicotine metabolism (CYP2A6) analyses of nicotine metabolism (variation in CYP2A6) or nicotinic acetylcholine receptor gene subtypes. All samples will be stored at the University of Vermont Tracy Lab.

Biomarker shipping and storage:
Biomarkers will be shipped quarterly to the University of Vermont Laboratory for Clinical Biochemistry Research (Tracy Lab). The Tracy Lab will serve as a central repository for all biomarker specimens and will be responsible for distributing specimens to the appropriate labs on a quarterly basis. Urine samples will be analyzed and stored at the University of Minnesota Hecht Lab. Cardiovascular Biomarkers will be analyzed and stored at the Tracey Lab. Additional blood samples for the purposes of phenotyping will be analyzed and stored at the University of Toronto Tyndale Lab.

Baseline fMRI testing (University of Vermont only):
Participants at the UVM site will complete the neuroimaging battery two or three days after the first baseline assessment, depending on availability. This battery will be completed only among a randomly selected subset of participants in the lowest and the highest dose conditions (45 participants/dose condition for total of 90 participants), which will provide the greatest likelihood of detecting differences between nicotine doses. Forty-five participants from each of the two conditions will be selected with the goal of having 20 completers from each of the doses. Participants who consent to neuroimaging and meet the eligibility criteria will be encouraged to abstain from smoking for approximately 24 hours before their scan. Abstinence will be verified by expired breath carbon monoxide levels that have decreased by at least 50% from the measure taken during the Baseline 1 visit. The battery includes fMRI assessments that parallel the behavioral/cognitive assessments described above (i.e., a sustained attention task, inhibitory control test of executive function) and that are sensitive to abstinence-related disruptions in performance.

Prior to Baseline scan, participants will partake in a practice session of the fMRI cognitive battery tasks in a mock scanner at the Clinical Research Center (CRC) in order to practice each task in an environment that closely mimics that of the actual fMRI machine itself.

The neuroimaging battery also includes a high-resolution anatomical scan to assess total and regional grey matter volumes and cortical thickness, a resting- state scan to assess intra- and inter-regional brain connectivity, and arterial spin labeling to provide a quantitative measure of blood flow. Baseline characterization and comparison with a second scan approximately 12 weeks later will provide the potential for insights into the neurobiology of dependence and withdrawal (including individual differences in dependence severity) and differential changes that may arise from being exposed for an extended period to VLNC versus usual nicotine content levels in commercially available cigarettes.

Experimental Procedures

Experimental Period:

Participants will be seen weekly throughout the 12-week experimental period. Weeks 2, 6, 12 and the abstinence visit will take approximately 2-4 hours each. All other sessions will last approximately 2 hours. Upon arrival at the laboratory, participants will provide urine and breath BAL and CO samples. If the participant has a positive urine toxicology screen the Research Assistant will initiate the Field Sobriety SOP to determine if the participant can continue with the session or if it should be rescheduled. At the end of each experimental session, the researcher will complete the End of Visit Evaluation Form, which will be filed in the participant's binder. This will allow the researcher to make note of any problems encountered during the visit, to track which computers were used for which tasks, and to assess the truthfulness of the participant in regards to self-report of tobacco use and compliance to study procedures.

Visit scheduling requirements for experimental period:

The ideal scheduling window between each visit is 7 days based on the date of the Baseline 2 Visit. For additional scheduling requirements, refer to the '*Scheduling Visits SOP*'. If a participant misses a visit and is not able to reschedule during the window (± 3 days), that visit will not be 'made-up' in the future. All measures that were not completed will be considered missing data and will not be collected during future visits. If a visit mistakenly occurs outside of the designated window, this is a protocol deviation. A 'Non-Medical Event Form' will need to be completed. Additionally, each visit should occur at approximately the same time of day ± 2 hours.

If a participant is not able to attend his/her Week 12 visit, then it should be rescheduled even if it is outside of the scheduling window. This will be documented as a protocol deviation.

Experimental Visits Weeks 1, 3, 5, 7, 9, and 11 Procedures

Measures/Assessments

Physiological Measures Collected, recorded on paper, and entered into REDCap by the interviewer at the end of the visit:

1) BAL
2) Weight
3) CO
4) Blood Pressure
5) Heart Rate
6) Urine Toxicology

The following questionnaires will be participant-administered via paper at and then will be entered into REDCap by the interviewer at the end of the visit:

1) BDI
2) OASIS

The following assessments will be administered as an interview and will be entered into REDCap by the interviewer at the end of the visit:

1) Concomitant Medications
2) Medical Event Form, if applicable
3) Health Changes Questionnaire
4) Time Since Last Cigarette Questionnaire

The following assessments will be completed by the participant directly in REDCap:

1) MNWS
2) QSU brief - Usual Brand Cigarette
3) QSU brief - Study Cigarette
4) Cigarette Evaluation Scale - Study Cigarette

In the event that the REDCap website is not functioning, the assessments will be printed out and administered on paper. The source documents will be kept in the participant's binder. The interviewer will enter the data into REDCap when it resumes functioning properly. This information should be recorded in the 'End of Visit Evaluation Form' and filed in the participant's binder.

Experimental Visits Weeks 2, 4, 6, 8, 10 and 12 Procedures:

Measures/Assessments

Physiological measures collected, recorded on paper, and entered into REDCap by interviewer at the end of the visit:

1) BAL
2) Weight
3) CO
4) Blood Pressure
5) Heart Rate
6) Urine Toxicology
7) Urine Pregnancy test (if applicable

The following questionnaires will be participant-administered via paper at and then will be entered into REDCap by the interviewer at the end of the visit:

1) BDI
2) OASIS

The following assessments will be administered as an interview and will be entered into REDCap by the interviewer at the end of the visit:

1) Concomitant Medications
2) Medical Event Form, if applicable
3) Health Changes Questionnaire
4) Time Since Last Cigarette Questionnaire

The following assessments will be completed by the participant directly in REDCap:

1) Respiratory Health Questionnaire (weeks 2, 6 and 12 only)
2) FTND
3) Perceived Health Risks Questionnaire (weeks 2, 6 and 12 only)
4) Smoking Stages of Change Algorithm and Contemplation Ladder (Week 12 only)
5) Cigarette Purchase Task - Usual Brand Cigarette Version (weeks 2, 6 and 12 only)
6) Cigarette Purchase Task - Study Cigarette Version (weeks 2, 6 and 12 only)
7) WISDM-Brief
8) Drug Use Questionnaire - 1 month version (weeks 6 and 12 only)
9) PANAS (weeks 2, 4, 6, 8, 10 and 12)
10) Perceived Stress Scale (weeks 2, 6, and 12 only)
11) Alcohol Use Questionnaire - 1 month version (weeks 6 and 12 only)

In the event that the REDCap website is not functioning, the assessments will be printed out and administered on paper. The source documents will be kept in the participant's binder. The interviewer will enter the data into REDCap when it resumes functioning properly. This information should be recorded in the 'End of Visit Evaluation Form' and filed in the participant's binder.

Participants will also complete the following tasks:

1) Cognitive tasks (weeks 2, 6 and 12 only)
2) Smoking Topography - study cigarette (weeks 2, 6 and 12 only)

Week 12 fMRI testing (University of Vermont only):

Participants at the UVM site will also complete the neuroimaging battery again to assess changes after extended exposure to different doses. Participants who have initiated a quit attempt will not be asked to smoke prior to the scan. Participants willing to smoke the research cigarettes will take two puffs 30 minutes prior to the scan. If the participant is unwilling to smoke the research cigarette, they will be allowed to smoke their usual brand.

<u>Biological Samples to be collected:</u>

1) First void urine sample (Weeks 6 and 12 only)
2) Blood Samples (Weeks 6 and 12 only)
3) Collect FeNO (Weeks 6 and 12 only)

Interactive Voice Response System:

Participants will continue to use the IVR system on a daily basis throughout the experimental period to record the number of study cigarettes smoked per day and use of non-study cigarettes. During the first week after Baseline 2, the IVR system will collect information about withdrawal symptoms.

Variable Incentive Program:

An incentive program has been developed with the goal of improving attendance at scheduled assessment sessions, compliance with using only study-provided tobacco products, and encouraging honest self-reports regarding all nicotine/tobacco use.

Briefly, participants will receive a total of five tickets for each weekly visit they attend after randomization (Visits 03-14, weeks 1-12). In total, participants could earn 60 valid tickets across the 12 visits. Participants will be instructed that these tickets correspond to attendance (one ticket), honest reporting (one ticket), and adherence to using only the assigned study product (three tickets). They will be further instructed that these tickets "could" be eligible for entry into a monthly drawing for prizes, but that only tickets that are "validated" will be eligible for prizes.

Since it is prohibitively expensive to test urine samples each week for each participant and because it is currently not feasible to detect with reasonable precision non-compliance based on biomarkers in the two higher nicotine group, we plan to only validate the attendance tickets. Hence, each participant who attends their regularly scheduled weekly session will have a total of five validated tickets entered into the monthly drawing.

To convey the message that we may be validating honest reporting and use of only study-provided products, we will collect a weekly urine specimen from participants. Further, in a bogus pipeline of sorts, participants will be instructed and that these urine specimens MAY be used to biochemically verify compliance to the study product by testing different nicotine and tobacco products found in the urine. Likewise, participants will also be instructed that their honesty ticket MAY be validated if their self-reported tobacco use matches what's in their urine. So there is some minor deception involved, but technically we could conduct urine toxicology testing for both purposes. Hence, if the urine toxicology testing is presented as something that MAY be done for validation purposes, we feel that any deception is relatively minor. For scientific/economic reasons we are just electing to restrict validation to attendance. Nevertheless, we will debrief all participants upon the completion of the trial. We will inform them that the incentive program was based exclusively on attendance due to the relatively high cost of urine toxicology testing and other practical problems with shipping the urines for prompt testing.

Drawings will be conducted on the 1st of each month. Validation will be performed by staff who have no participant interaction and are not blind to condition. Any ticket drawn will be eligible for an incentive as the only true contingency is for attendance. There will be no mention of the basis for earning incentives (i.e., whether the ticket was for attendance, honesty, adherence,). Participants will simply be informed that he or she earned an incentive from the drawing.

Each drawing will be independent (without replacement); consequently, some participants will not win a prize and others may win more than one during the study if more than one of their tickets is drawn. After confirming winners, the remaining tickets from each month will be discarded (i.e., tickets will only be entered into one drawing). The monthly prize amounts are

detailed below.

We estimate based on the 2½ years we estimate it will take to complete this study, that participants will win an average of approximately $65 in prizes or an additional $5.50 per week per participant.

Grand Prize (1): $500 cash
Second Prize (1): $200 cash
Third Prize (5): $10 cash

Product and Procedures Compliance Review Sessions:
At each visit, Baseline 2 through Week 11, participants will be counseled about their use of the study cigarettes. Participants will be asked about any concerns or obstacles associated with use of the study cigarettes. The importance of honest self-reporting will be stressed. Participants will be told that they will not be penalized for use of other nicotine or tobacco products and that it is crucial for them to report any use of these products. If difficulties are encountered, participants will be asked why they think they are experiencing difficulties (e.g., taste, withdrawal symptoms) and to problem-solve how to deal with these difficulties in order to meet the protocol requirements. Additionally, participants will be counseled about their IVR completion, visit attendance, task engagement and product accountability. Refer to the '*Product and Procedures Compliance Review Sessions SOP*' for more information.

Quit Attempts During the Study Protocol:
At each weekly session, we will ask the participant if he/she is currently abstaining from smoking with the intention of quitting. If the answer is no, then we will also ask if he/she is planning to quit smoking prior to his/her next scheduled visit.

If a Participant is Currently Abstaining from Smoking with the Intention to Quit:

- Encourage participant to continue abstaining from smoking
- Schedule the participant for normal weekly visits, but no puff topography
- Provide the participant with the '*Clearing the Air*' manual and local smoking cessation resources
- Give the participant the option to take home study product rather than require him/her to take the product
- If the participant choses to take home the study product have him/her sign a form acknowledging that cigarette availability could be detrimental to the quit attempt. Recommend that he/she put the product "away" at home as to avoid unwanted cues to smoke.
- If the participant chooses not to take home the study product, have him/her contact the lab if he/she lapses and would like to pick up or be mailed the study product prior to his/her next visit.

If a Participant is Planning to Quit Smoking, but has not initiated the quit attempt:

- Ask if he/she has identified a target quit date and, if so, what that target date is.
- Provide the participant with the '*Clearing the Air*' manual and local smoking cessation resources.
- Provide the participant with the study product as usual. Recommend that on the target date he/she put the product "away" at home as to avoid unwanted cues to smoke.

Abstinence Assessment Session:

After the week 12 visit, participants will be required to come back for one additional visit the following day. During this visit, participants will have been encouraged to abstain from smoking until their next scheduled visit (approximately 24 hours later). The abstinence assessment session should be scheduled no less than 18 hours and no more than 30 hours after the Week 12 visit. Abstinence will be verified by expired breath carbon monoxide levels that have decreased to $\leq$ 4 ppm. This session will allow us to determine whether the experimental cigarettes have reduced the effects of abstinence on these measures relative to the control conditions. If the participant does NOT meet abstinence criteria, he/she will only receive $20 for the visit.

Measures/Assessments

Physiological measures collected, recorded on paper, and entered into REDCap by the interviewer at the end of the visit:

1) BAL
2) CO
3) Blood Pressure
4) Heart Rate
5) Urine Toxicology

The following questionnaires will be participant-administered via paper at and then will be entered into REDCap by the interviewer at the end of the visit:

1) BDI
2) OASIS

The following assessments will be administered as an interview and will be entered into REDCap by the interviewer at the end of the visit:

1) Concomitant Medications
2) Medical Event Form, if applicable
3) Health Changes Questionnaire
4) Time Since Last Cigarette Questionnaire

The following assessments will be completed by the participant directly in REDCap:

1) MNWS
2) QSU-brief - Usual Cigarette
3) QSU-brief - Study Cigarette
4) Cigarette Purchase Task - Usual Brand Cigarette Version
5) Cigarette Purchase Task - Study Cigarette Version
6) Cigarette Evaluation Scale – Usual Brand Cigarette Version

In the event that the REDCap website is not functioning, the assessments will be printed out and administered on paper. The source documents will be kept in the participant's binder. The interviewer will enter the data into REDCap when it resumes functioning properly. This

information should be recorded in the ‘End of Visit Evaluation Form’ and filed in the subject’s binder.

Participants will also complete the following task:

1) Cognitive tasks
2) University of Vermont only: Participants at the UVM site will also complete the neuroimaging battery again to assess changes after extended exposure to different doses.

Participants who do <u>NOT</u> meet abstinence criteria will be required to complete the following assessments:

1) BAL
2) CO
3) Blood Pressure
4) Heart Rate
5) Urine Toxicology
6) Concomitant Medications
7) Health Changes Questionnaire
8) Medical Event Form, if applicable
9) TLFB

Participant Compensation:
Participants will receive $25 for completing the screening visit, plus an additional $25 bonus for completing the visit on time as scheduled. Payment will be made regardless of enrollment as long as the participant passes the drug test, breath alcohol test, and meets the minimum requirements for carbon monoxide or NicAlert levels. Participants who do not pass these tests will be dismissed from the screening visit without payment, except in the event they can produce a prescription for the medication that caused them to fail the drug test. Participants will receive $100 for each of the shorter sessions (Baseline 1, Weeks 1, 2, 3, 4, 5, 7, 8, 9, 10, 11), $150 for each of the longer sessions (Baseline 2, Weeks 6 &12), up to $160 for the abstinence visit ($150 for the visit + up to $10 for the preference test), $20 for biochemical verification of abstinence, up to $221 for completing daily IVR reports of study cigarette and other nicotine and tobacco use. Participants will also have a chance to earn an additional $50 bonus for every three visits that are completed on time as scheduled. There will also be a $100 bonus for completing the study for a total bonus of $325. If the participant does not attend the screening visit or one of the weekly visits as scheduled, they will forfeit the bonus. They will have a chance to earn another bonus payment with the next set of three visits. Participants who do not complete the entire study will receive compensation for the sessions that they do complete. UVM participants who undergo fMRI testing will receive an additional $150/scan. Total compensation for completing Study 2, including study visit payments, daily IVR calls and bonuses is $2301 (or $2601 if participating in the fMRI testing). Participants will also have a chance to earn additional money through the Variable Incentive program. As mentioned above, participants will have a chance to earn additional incentives each month for compliance, honesty and attendance, however, we anticipate that on average, participants will win approximately $150 in prizes.

End of Study:
After a participant has completed all study procedures and has been paid for participation the research assistant will read the following script and give the participant the *Clearing the Air Manual.*

"If you've reduced your smoking during this study, we encourage you to continue these reductions or even consider quitting. We would like to provide you with some resources should you decide to try to abstain from smoking (give "Clearing the Air" and hotline information). Please also feel free to consult with your physician and use any medications he/she deems appropriate. We will call you in approximately 30 days to ask about your smoking since leaving the study. There is no right answer and we know how difficult quitting can be. Please just answer honestly. The call will take less than 5 minutes. Thanks again for your participation."

The following assessments will be administered using REDCap:

1) End of Study Questionnaire

30 Day Follow up Phone Call:
Participants will receive a follow-up phone call between 25 and 35 days after the abstinence assessment session to assess their smoking patterns. The phone questionnaire will last less than five minutes. The questionnaire will ask if the participant is still smoking, how much and whether he/she has attempted to quit smoking since the end of the study. Participants will receive 5 variable incentive program lottery tickets for completing the call as compensation. Those who report abstinence will be invited to come in for biochemical verification and be compensated $40 for doing so. A urine sample will be collected to test urine cotinine levels. Additionally, any Medical Event Forms that remain open from the last session will be discussed. If the participant became pregnant during the study, this would have been recorded as a medical event. During this phone call, the research assistant will confirm her due date. This event will remain open until delivery. At that time the licensed medical professional will contact the participant to ask a few questions about the baby's health and will update the Medical Event Form.

Once a participant has completed all study procedures and all open events have been closed, the PI will review the participant's binder and sign a form indicating study completion for that participant.

Randomization
At the end of the Baseline 1 session, participants will be randomized into one of three cigarette conditions. Participants in each condition will be assigned cigarettes that match their menthol preference. Participants will be randomized, using block randomization, in equal number to the dose conditions, with randomization stratified by study site and menthol status. Each site will randomize participants until the total goal of 282 participants across both sites is reached, and no effort will be made to recruit a specific number of menthol and non-menthol smokers at each site.

Condition	TPMF Code	Type*	Specifications Nicotine Yield	Specifications Tar Yield	Specification Range for Nicotine Yield	Specifications Nicotine Content
1	NRC600	CN	0.8 ± 0.15	9 ± 1.5	0.65 - 0.95	15.30 ± 0.18
1	NRC601	CN-Men	0.8 ± 0.15	9 ± 1.5	0.65 - 0.95	16.03 ± 0.47
2	NRC300	RN	0.12 ± 0.03	9 ± 1.5	0.09 - 0.15	2.27 ± 0.08
2	NRC301	RN-Men	0.12 ± 0.03	9 ± 1.5	0.09 - 0.15	0.104 ± 0.002
3	NRC102	RN	0.03 ± 0.01	9 ± 1.5	0.02 - 0.04	0.37 ± 0.01
3	NRC103	RN-Men	0.03 ± 0.01	9 ± 1.5	0.02 - 0.04	0.39 ± 0.00

*Legend:	
RN	Reduced Nicotine
RN-Men	Reduced Nicotine-Menthol
CN	Conventional Nicotine
CN-Men	Conventional Nicotine-Menthol

The lead statistician will create a randomization schedule for each of the two sites, amounting to 150% of expected enrollment at each site. The excess randomization codes will be used in the event that a site will have to enroll extra participants due to unexpectedly slow enrollment at another site. The nicotine doses will be identified by letter code and the number 2 (V2, W2, X2, Y2) and only Administrative Core personnel with no participant contact will have the link between the statistician's letter code and dose assignments. The randomization schedules and the link between the alphabetic code and treatment assignment will be maintained securely by the Administrative Core. A second, sealed, copy will be secured in a separate building to protect against loss related to fire or other unforeseen events.

The University of Vermont will be responsible for removing all identifying information from cigarettes received from the Research Triangle Institute (RTI), labeling each carton with a blind code, assigning product using this blind code based on the randomization schedule being provided by the UVM Biostatistics Core, and shipping cigarettes to each site as needed based on recruitment. Each site will be responsible for tracking product received and distributed to participants, collecting unused product from participants, and returning unused cigarettes to

UVM. The participants, investigators and study staff will not have knowledge of which product is given to a participant or whether different participants received the same or different product.

During the experimental period, participants will be provided with a 14-day supply of research cigarettes equivalent to 150% of their daily smoking rate. This rate will be calculated at Baseline 2 and will be an average daily smoking rate based on the IVR data for the first seven days of the baseline period. This will ensure adequate availability of cigarettes in the numerous locations participants may typically keep a supply (home, work, vehicle, etc.) as well as avoid expending the entire supply if they miss a scheduled visit. Participants will be instructed to use the research cigarettes for 12 weeks, at which point they are to discontinue product use.

If there is prior knowledge a participant will be missing a visit (i.e. planned vacation, laboratory closure, etc.), then the participant will be provided with an adequate supply of cigarettes to make up for the missed visit(s). The participant will be given a 21-day supply if one visit is going to be missed and a 28-day supply if two visits are going to be missed.

Participants will be asked to refrain from use of other non-study cigarettes during the study period. If participants have to use another nicotine product, they will be told to use a non-combustible product (gum, patch, etc.). Additionally, they will be told there is not a penalty for use of non-study cigarettes, and that it is crucial for them to report any use of non-study cigarettes or other nicotine or tobacco products. Throughout the baseline and experimental periods, an Interactive Voice Response (IVR) system will be used on a daily basis to record the number of study cigarettes and non-study cigarettes used the previous day. During the baseline and first experimental week, participants will also answer daily IVR questions about their mood. Participants will be seen weekly for assessments. Brief standardized review sessions focusing on compliance with the study cigarettes and other study procedures will be provided at each visit. At the end of the 12-week trial, participants will undergo an assessment of withdrawal, craving, and cognitive function following a brief period of abstinence.

Product Accountability:

Participants will be required to keep track of all the cigarettes provided to them. Therefore, they will be instructed to return all unused cigarettes and empty cigarette packs to the laboratory each week. Research staff will complete the 'Product Accountability Log' with the participants. Any discrepancies in the product dispensed versus product returned will be discussed and recorded in the log. Empty cigarette packs will not be saved. Unused cigarette packs will be re-distributed to the participants during Weeks 1-11. During Week 12, any remaining unused cigarettes returned by the participants will be collected by the research staff.

Participants who report running out of cigarettes prior to a scheduled weekly visit will be allowed to come in for an unscheduled visit to obtain more research cigarettes. If a participant has more than two unanticipated visits we will determine if a rate change is necessary. To determine this, we will look at the past two CO levels as compared to the Baseline 2 CO. If the CO trend is consistent with the self-report of smoking all of the allotted cigarettes then a rate increase will be granted. The participant will then receive cigarettes at a rate of 175% of their daily smoking rate. The maximum increase is 200% of their daily smoking rate. If participants lose more than two packs of cigarettes and require an unscheduled visit to the laboratory to supplement their

supply, they will be told the next time they lose more than two packs they will have to wait until their next scheduled appointment to receive more cigarettes.

Statistical Methods and Sample Size

Statistical methods. See Statistical Analysis Plan at the end of this Supplemental document.

Sample size. Sample size for other analyses was determined using power analysis for hypothesis tests related to the Primary Aim of Study 2, specifically to detect a significant difference between the reduced-nicotine conditions and the high-nicotine yield condition in the primary endpoints, cigarettes per day (CPD) and urine cotinine, at the end of the trial. Donny et al. (2015) found a reduction of 4.52 CPD and 6.07 CPD among subjects smoking 2.4 mg/g and 0.04 mg/g cigarettes, respectively, compared to those smoking normal nicotine cigarettes. In addition, they reported a decrease of 0.59 and 0.39 in urine cotinine among those smoking these same RNC cigarettes, compared to those smoking NNC cigarettes. A sample size of 69 completers per condition will provide 90% power to detect similar differences in CPD and greater than 95% power to detect differences in urine cotinine, with a two-sided type I error rates of 0.02. The type I error rate reflects the Bonferroni correction needed to allow testing of all pair-wise comparisons. Regarding fMRI power, the analysis was based on the estimated effect size of 2.04 (Cohen's d) from the cortical activation differences previously observed between smokers and ex-smokers on the same inhibitory control task proposed here (Nestor et al., 2011). With 20 completers in each condition, there is 80% power at $p = 0.05$ to detect effects about half as large (Cohen $d=0.91$) between any two conditions.

Potential Risks of Participation

1) <u>Survey Questionnaires</u>: The interview will include questions about medical history, drug and alcohol use, and questionnaires about mood. Answering these personal questions could make the participant feel uncomfortable.
2) <u>Breach of Confidentiality</u>: The risk of the interview is loss of privacy if other people find out the results.
3) <u>Coercion</u>: Coercion is a possible risk due to monetary compensation for participating in these studies. The likelihood of this risk is low because the compensation is commensurate with the amount of time and effort required for these studies.
4) <u>Drug Testing</u>: A breach of confidentiality could occur and other people could learn of the participant's drug use.
5) <u>Obtaining blood pressure</u>: The blood pressure cuff may cause minimal discomfort. In obtaining blood pressure, researchers may find out the participant has abnormal blood pressure.
6) <u>Smoking Cigarettes</u>: All cigarettes are detrimental to a person's health and can lead to significant medical problems including:
    a. Cardiovascular Diseases: Coronary heart disease, heart attack, stroke, peripheral vascular disease, reduced blood circulation, abdominal aortic aneurysm
    b. Respiratory Diseases: Emphysema, bronchitis, and chronic airway obstruction
    c. Cancers: Cancer of the lung, bladder, cervix, esophagus, kidney, larynx, mouth, pancreas, throat, and stomach; leukemia
    d. Metabolic Diseases: Type 2 Diabetes

e. Other Health Risks Associated with Smoking: Including but not limited to infertility, lower bone density in postmenopausal women, and hip fracture in women
f. Death

7) Smoking study cigarettes: In addition to the above medical problems, participants may experience some minor adverse health effects such as headaches or experience withdrawal symptoms, which are listed below. Due to the altered nicotine levels, there could be a change in their cigarette use including the manner in which they inhale the smoke. Smoking the study cigarettes does not provide any less risk than their usual brand cigarette and could pose increased health risks. Participants may also experience increases in levels of carbon monoxide, a gas from smoke.

8) Smoking Withdrawal: Participants may experience smoking withdrawal symptoms during this study. The symptoms can be uncomfortable but are typically of minimal risk. Smoking withdrawal symptoms include:
a. Anger, irritability, frustration
b. Anxiousness, nervousness
c. Depressed mood or sadness
d. Desire or craving to smoke
e. Difficulty concentrating
f. Increased appetite, hunger or weight gain
g. Insomnia, problems sleeping or awakening at night
h. Restlessness
i. Impatience
j. Constipation
k. Dizziness
l. Coughing
m. Dreaming or nightmares
n. Nausea
o. Sore Throat

9) Returning to Regular Smoking: It is possible that if participants return to smoking their usual brand of cigarette at the end of the study they may experience mild and transient nausea, dizziness, and lightheadedness.

10) Risk to Fetus**:** Smoking during pregnancy can lead to miscarriage, preterm delivery, stillbirth, low birth weight, problems with the placenta, birth defects such as cleft palate, sudden infant death syndrome (SIDS), and early childhood behavioral problems.

11) Changes in blood pressure and/or heart rate: Smoking and nicotine can affect the cardiovascular system, which may result in changes in blood pressure and/or heart rate.

12) Exacerbation of psychiatric symptoms: Smoking and nicotine can affect a person's mood and emotions and are associated with psychiatric disorders including major depressive disorder, general anxiety disorder, bipolar disorder and eating disorders. Any changes in nicotine or cigarettes consumption could adversely affect psychiatric conditions.

13) MRI: The MRI scanner produces a loud banging noise and may be uncomfortable for people who become anxious in confined spaces. The presence of metal in or on a participant's body during an MRI scan can present a serious health risk. The MRI staff will ask participants in detail about any possible metal they may have in or on them. Regarding unexpected MRI findings, the participant will be informed of what was found. In addition, information about the incidental finding can be provided to the participant's primary doctor or the study team can refer them to an appropriate specialist. The costs

for any care that would be needed to diagnose or treat an incidental finding would not be covered by the research study and would be the responsibility of the participant.

<u>Avoiding Risks to Fetus:</u>

If participants choose to be sexually active, they should use an appropriate “double barrier” method of birth control (such as female use of a diaphragm, or contraceptive sponge, in addition to male use of a condom) or the female should be using prescribed “birth control” pills, patch, ring, injections, or implants or intrauterine device (IUD). Participants will be tested for pregnancy every two weeks beginning at screening through the last study visit. If a participant becomes pregnant during the study, she will be withdrawn from the study. Approximately 30 days after being withdrawn or having a positive pregnancy test at the last study visit, the research staff will call the participant to confirm her due date. The licensed medical professional will follow-up with the participant after delivery to ask questions about the baby’s health.

Expected benefits of participation:

There are no immediate benefits from participating in the study. The information obtained from this study may ultimately help the Food and Drug Administration decide how best to regulate tobacco products with the goal of improving public health.

Study Debriefing:

After data collection is complete, participants will receive a letter telling them which condition they were randomized into and the results of the study thus far.

Protection Against Risk

Research data without identifiers will be maintained in a locked file cabinet and on password-protected computers in the research staff workplace, with only code numbers identifying subjects. Study consent forms and the linkage between the participants’ names and codes will be stored in a locked file cabinet. Interviews with participants will be conducted in private rooms. Urine samples for drug and pregnancy tests and tobacco exposure biomarkers will be obtained in a private bathroom within the laboratory suite. Blood draws will be performed in a private patient room. Subjective measures will be administered electronically. The biostatistics and data-management team will provide consistent data-management practices for all data in the Center. Validity and reliability of data will be maximized by using REDCap, which is housed on the Fletcher Allen Health Care, HIPAA compliant, computing system. REDCap is a secure, web-based system that accommodates local and remote data collection by each project team, and allows for data entry work-flow monitoring and data quality control monitoring by biometry staff. For data integrity, data entry windows will follow the structure of paper forms as much as possible to allow for ease of entry, and will use predefined choices to minimize errors when possible. Data quality monitoring will be facilitated with periodic down loads and analysis using a variety of common statistical program format such as SAS, Stata, R, and SPSS. Quality control procedures will be conducted for all data collected, including analysis of missing data and logic checks for out of range and other anomalous values. This secure electronic data gathering and transmission plan, overseen by the experienced biostatistical team, will minimize opportunities for breaches of confidentiality. Biological samples for nicotine and carcinogen biomarker analysis will be marked with participant ID, stored in the locked laboratory suite, and sent to a laboratory for analysis on a quarterly basis.

All information collected as part of this study will be accessible only to research staff. No information will be shared with participants' clinicians unless the participant requests this in writing. All investigators and staff have undergone (and any new staff will undergo) human subjects' ethics training as required by UVM and are fully conversant with relevant ethical principals around confidentiality. Assessments, consenting and study procedures will be closely supervised by the PI.

The sponsors (NIDA/FDA) as well as the Institutional Review Board and regulatory authorities could be granted direct access to original medical and research records for verification of clinical trial procedures and/or data. If this is required, it will be done under conditions that will protect privacy to the fullest extent possible consistent with laws relating to public disclosure of information and the law-enforcement responsibilities of the agency.

Data Storage:
Data will be stored locally at each site, at the University of Minnesota Masonic Cancer Center's Bioinformatics Core and at the University of Vermont. Long-term storage of all study data, for at least 7 years after study completion, will be at the University of Vermont.

Adverse Events
The research assistant will ask about adverse events at each session, using a form that assesses the nature, severity, duration, action taken, and outcome of study-related adverse events. AEs will be captured from the time of first study cigarette. Participants will be given contact cards to inform us of events that occur between study contacts. Any AE that remains open will be reviewed and closed at an interview conducted 30 days after the study completion date (completers) or when the study should have ended had the participant completed the study (dropouts and those withdrawn by investigator).

All procedures will be monitored to ensure that they conform to the approved protocol. In addition, monitoring will be done of all unforeseen circumstances that might arise and affect safety; of all reports of serious adverse events as defined in 38 CFR 46 (death, new or prolonged hospitalization, persistent or significant disability/incapacity); of other significant adverse events (adverse events that lead to drop out by the participant or termination by the investigator); of unexpected adverse events resulting from the study, and of expected adverse events.

Any SAE will be brought to the attention of the site PIs as soon as possible and not longer than 24 hours. Any AE or SAE that is both unexpected and related to study participation will be reported to the IRB within 7 days of the event. The local IRB will make a determination as to whether additional reporting requirements are needed. IRB actions will be reported to the funding agency by the PIs no less than annually and more frequently as recommended by the local IRB. Any SAEs will be summarized in the yearly Progress Reports to the funding agency, including a review of frequency and severity. All SAEs will be followed through ongoing consultation with the physician caring for the patient until they resolve, result in death, or stabilize and are not expected to improve. The study staff will be in close contact with participants and health care providers throughout the study to monitor for potential unanticipated

problems. Any unanticipated problems will be discussed at the weekly research staff meetings and reported as required to the local IRB.

Withdrawal or Monitoring of Participants

For the participant's protection, participants will be withdrawn immediately from the study if any of the following occur:

1) Cardiovascular disease (CVD) event: Typically includes MI (heart attack), PTCA (angioplasty/stenting), bypass surgery, stroke, peripheral vascular disease (arterial blockages in arms or legs leading to procedure or surgery). Less common CVD problems would be new cardiac arrhythmias (e.g., new atrial fibrillation) or new valvular disease (e.g., mitral or aortic regurgitation).
2) DVT/PE (deep vein thrombosis/pulmonary embolism, i.e., blood clots in the venous system).
3) Suicide Attempt: A participant will be withdrawn if he/she attempts suicide at any time during participation in the study.
4) Psychiatric Hospitalization: A participant will be withdrawn if he/she is hospitalized for psychiatric reasons at any time during participation in the study.
5) Pregnancy: If participant indicates she is pregnant or has a positive pregnancy test at any time during the study, she will be withdrawn from the study, and this event will remain open until delivery. At that time the licensed medical professional will contact the participant to ask a few questions about the baby's health and will update the open 'Medical Event Form'. A positive pregnancy test at Session 14 in Study 1 or Week 12 in Study 2 will trigger a 'Medical Event Form' to be completed but will not result in withdrawal since she is no longer receiving study product.
6) Expired breath carbon monoxide increase: A participant will be withdrawn from the study if the average of two consecutive CO readings during the same visit is 100 ppm or greater.
7) Marked increase in smoking: A participant will be withdrawn from the study if he/she meets **BOTH** of the following criteria for two consecutive weeks
   a. Cigarette per day increase: The average CPD increases by more than 100% from the average CPD during baseline.
   b. Expired breath carbon monoxide increase: If the average of two consecutive CO measurements in the same visit is
      i. CO is greater than 50 ppm if CO at Baseline 1 is <20 ppm.
      ii. CO is greater than 60 ppm if CO at Baseline 1 is 20 – 34 ppm.
      iii. CO is greater than 70 ppm if CO at Baseline 1 is 35 – 49 ppm.
      iv. CO is greater than 80 ppm if CO at Baseline 1 is 50 – 64 ppm.
      v. CO is greater than 90 ppm if CO at Baseline 1 is 65 – 80 ppm.
8) Note: If the second consecutive visit is the last study visit, then the participant would not be withdrawn from the study.

The following will be monitored and can lead to the participant being withdrawn by the PI or Licensed Medical Professional:

1) Cigarettes per day increase: Continued participation will be evaluated by the site PI if the average number of cigarettes per day (CPD) increases by more than 100% from the average CPD during baseline as determined by CPD at Baseline 2.

2) Blood pressure (BP) or heart rate (HR) changes: If any of the following occur post-enrollment: 1) BP is at or above 160/100 or below 90/50, or 2) HR is at or above 115 bpm or below 45 bpm a manual blood pressure and heart rate measurement will be taken after 10 minutes have passed. If the manual reading is still out of range, a 'Blood Pressure and Heart Rate Symptom Checklist' and 'Medical Event Form' will be completed, and the participant will be monitored by the medical professional.
3) Expired breath Carbon Monoxide increase: If the average of two consecutive CO measurements meets the criteria below then the 'Medical Event Form' will be completed and the participant will be monitored by the licensed medical professional.
    a. CO is greater than 50 ppm if CO at Baseline 1 is <20 ppm.
    b. CO is greater than 60 ppm if CO at Baseline 1 is 20 – 34 ppm.
    c. CO is greater than 70 ppm if CO at Baseline 1 is 35 – 49 ppm.
    d. CO is greater than 80 ppm if CO at Baseline 1 is 50 – 64 ppm.
    e. CO is greater than 90 ppm if CO at Baseline 1 is 65 – 80 ppm.
4) Any hospitalization or debilitation in which participation in the study could be detrimental to the recovery process. This will be self-reported by the participant and will be reviewed by the site PI and licensed medical professional to determine whether continued participation in the study is appropriate.
5) If a participant is behaving in an inappropriate or threatening manner, admits to lying about eligibility criteria, is participating in other smoking research studies that could affect the primary outcome measures, etc., then the PI can withdraw him/her from the study at the PI's discretion.
6) If a participant fails to attend regularly scheduled research assessment visits or comply with the research procedures or schedule, then the PI can withdraw him/her from the study at the PI's discretion.
7) Increase in psychiatric symptoms: Exacerbation in symptoms noted during the study (i.e., change in BDI category from mild to moderate or moderate to severe) will trigger review by the study's licensed medical professional. The PI will withdraw the participant upon the licensed medical professional's recommendation.

Data Safety Monitoring Board

A Data and Safety Monitoring Board (DSMB) has been established to monitor safety outcomes and will be comprised of five members. The DSMB will be chaired by Dr. Eden Evins, Associate Professor of Psychiatry at Harvard Medical School and Director of the Center for Addiction Medicine at Massachusetts General Hospital. Other members include: Kevin Delucchi, PhD., Professor in Residence of Biostatistics in Psychiatry at the University of California San Francisco and Director of the Quantitative Core of the San Francisco Treatment Research Center; Hendree E. Jones, Ph.D., Professor of Obstetrics and Gynecology and Director of UNC Horizons at University of North Carolina Chapel Hill; Wallace Pickworth, Ph.D., Research Leader, Baltimore Operation, Centers for Public Health Research and Evaluation, Battelle; Kimber Richter, Ph.D., M.P.H., Associate Professor of Preventive Medicine and Public Health at the University of Kansas and Director of the University of Kansas Hospital's tobacco treatment program.

Conflict of interest

None of the members will be otherwise affiliated with the center and each member will complete a conflict of interest disclosure form prior to each meeting. Ad hoc specialists may be invited to participate as non-voting members at any time if additional expertise is desired.

Monitoring activities and frequency of meetings

The DSMB will set their own agenda and decisions about monitoring; e.g. how frequently to monitor, what threshold requires changes to protocol or stopping the study, and whether to view raw or analyzed data. The DSMB will be given FDA and EMEA guidelines for DSMBs and recent reviews on DSMBs. A brief report will be generated from each meeting for the study record and forwarded to each of the study site's Institutional Review Boards (IRB) and NIDA's Program Officer with the progress report. The DSMB will be available to convene outside of the regular meetings, if necessary. If concerns should arise regarding a particular subject, or any troublesome trends in the experiences of participants, they will make appropriate recommendations for changes in protocol, as needed. The project investigators will continue to examine safety data, blind to study condition, in case they wish to make study modifications. Before modifications are made, they will inform the DSMB and request their comments.

Communication plan to IRB, NIDA, and FDA (if applicable)

All IRBs, the FDA and the NIDA's Program Officer will be informed of any significant action taken as a result of the Data and Monitoring Board's findings. Study Participants will be informed of any changes in risk.

Protection of confidentiality

For DSMB meetings only de-identified data, including blinded study site and condition type, will be provided to the board. All data and discussion during the meeting will be confidential.

Investigational Tobacco Product

The Vermont Center on Tobacco and Regulatory Science has received an Investigational Tobacco Product (ITP) application from the FDA to cover the experimental cigarettes being used in this study. This application encompasses both trial sites.

Certificate of Confidentiality

To help protect the participant's privacy, Dr. Stephen Higgins, PhD, has received a Certificate of Confidentiality from the National Institutes of Health. With this certificate, the researchers cannot be forced to disclose information that may identify the participants, even by a court subpoena, in any federal, state, or local civil, criminal, administrative, legislative, or other proceedings. The researchers will use the Certificate to resist any demands for information that would identify the participants, except as explained below. The Certificate cannot be used to resist a demand for information from personnel of the United States Government that is used for auditing or evaluation of federally funded projects or for information that must be disclosed in order to meet the requirements of the Federal Food and Drug Administration (FDA).

The Certificate of Confidentiality does not prevent the participant or a member of their family from voluntarily releasing information about themself and their involvement in the research. If an insurer, employer or other person obtains the participant's written consent to receive research information, then the researcher may not use the Certificate to withhold that information.

The Certificate of Confidentiality does not prevent the researchers from disclosing voluntarily, without consent, information that would identify the individual as a participant of the research project in instances such as evidence of child abuse or a participant's threatened violence to self or others.

Outcome Variables

Primary Endpoints:

1) Total number of cigarettes smoked per day (CPD) during Week 12 is the primary outcome;

Secondary Endpoints:

1) Study CPD during Week 12, total and study CPD across weeks, simulated consumer demand
2) Measures of adherence: non-study cigarette use, drop-out rate
3) Measures of psychiatric symptoms: BDI, OASIS
4) Measures of discomfort/dysfunction: MNWS, QSU
5) Measures of other health-related behaviors: breath alcohol, urine drug screen, TLFB-drug use, Alcohol Use Questionnaire, Drug Use Questionnaire, weight
6) Measures of nicotine/tobacco dependence: FTND, WISDM
7) Measures of tobacco exposure: CO, total nicotine equivalents, NNAL, minor alkaloids
8) Measures of intention to quit: Stages of Change, Contemplation Ladder
9) Measures of compensatory smoking: puff topography, filter analysis
10) Measures of other tobacco use: TLFB-other tobacco
11) Measures of cigarette characteristics: CES
12) Measures of cognitive function: BRIEF-A, EQ-5D, TPQ, D-KEFS, WASI-II, DDT, SST
13) Measures of cardiovascular function: heart rate, blood pressure, urine 11-dehydroTXB2
14) Measures of perceived risk: Perceived Health Risk Questionnaire
15) Safety outcome variables: Adverse Events (AEs), Serious Adverse Events (SAEs)

References

Beck, A. T., Ward, C., & Mendelson, M. (1961). Beck depression inventory (BDI). *Archives of General Psychiatry, 4,* 561-571.

Benowitz, N. L., & Henningfield, J. E. (1994). Establishing a nicotine threshold for addiction. The implications for tobacco regulation. *New England Journal of Medicine, 331,* 123-125.

Blalock, J.A., Robinson, J.D., Wetter, D.W., Schreindorfer, L.S., & Cinciripini, P.M. (2008). Nicotine withdrawal in smokers with current depressive disorders undergoing intensive smoking cessation treatment*. Psychology of Addictive Behaviors, 22*(1), 122-128.

Blank, M. D., Disharoon, S., & Eissenberg, T. (2009). Comparison of methods for measurement of smoking behavior: Mouthpiece-based computerized devices versus direct observation. *Nicotine & Tobacco Research, 11,* 896-903.

Brauer, L.H., Hatsukami, D., Hanson, K., & Shiffman, S. (1996). Smoking topography in tobacco chippers and dependent smokers. *Addictive Behaviors, 21*(*2*), 233-238.

Breslau, N., Kilbey, M.M., & Andreski, P. (1992). Nicotine withdrawal symptoms and psychiatric disorders: findings from an epidemiologic study of young adults. *The Journal of American Psychiatry, 149(4)*, 464-469.

Breslau, N., Novak, S.P., & Kessler, R.C. (2004). Psychiatric disorders and stages of smoking*. Biological Psychiatry, 55(1)*, 69-76.

Castaneda, A.E., Suvisaari, J., Marttunen, M., Perälä, J., Saarni, S.I., Aalto-Setälä, T., … Tuulio-Henriksson, A. (2008). Cognitive functioning in a population-based sample of young adults with a history of non-psychotic unipolar depressive disorders without psychiatric comorbidity. *Journal of Affective Disorders, 110(1-2),* 36-45.

Cohen, J. (1988). *Statistical power analysis for the behavioral sciences* (2nd ed.). New Jersey: Lawrence Erlbaum Associates, Publishers.

Cohen, S., Kamarck, T., & Mermelstein, R. (1983). A global measure of perceived stress. *Journal of Health and Social Behavior, 24(4),* 385-396.

Cox, L. S., Tiffany, S. T., & Christien, A. G. (2001). Evaluation of the brief questionnaire of smoking urges (QSU-brief) in laboratory and clinical settings. *Nicotine & Tobacco Research, 3,* 7-17**.**

Dedert, E.A., Calhoun, P.S., Harper, L.A., Dutton, C.E., McClernon, F.J., & Beckham, J.C. (2012). Smoking withdrawal in smokers with and without posttraumatic stress disorder. *Nicotine & Tobacco Research, 14(3),* 372-376.

Di Matteo, V., Pierucci, M., Di Giovanni, G., Benigno, A., & Esposito, E. (2007). The neurobiological bases for the pharmacotherapy of nicotine addiction. *Current Pharmaceutical*

Design, 13(12), 1269-1284.

DiClemente, C.C., Prochaska, J.O., Fairhurst, S.K., Velicer, W.F., Velasquez, M.M., & Rossi, J.S. (1991). The process of smoking cessation: an analysis of precontemplation, contemplation, and preparation stages of change. *Journal of Consulting and Clinical Psychology, 59(2),* 295-304.

Dierker, L., & Donny, E. (2008). The role of psychiatric disorders in the relationship between cigarette smoking and DSM-IV nicotine dependence among young adults. *Nicotine & Tobacco Research, 10(3),* 439-446.

Donny, E.C., Denlinger, R.L, Tidey, J. W., Koopmeiners, J. S., Benowitz, N. L.,Vandrey, R. G., … Hatsukami, D. K. (2015). Randomized trial of reduced-nicotine standards for cigarettes. *New England Journal of Medicine* 373(*Suppl* 14): 1340-1349.

Ernst, M., Heishman, S. J., Spurgeon, L., & London, E. D. (2001). Smoking history and nicotine effects on cognitive performance. *Neuropsychopharmacology, 25,* 313-319.

Goodwin, R.D., Zvolensky, M.J., Keyes, K.M., & Hasin, D.S. (2012). Mental disorders and cigarette use among adults in the United States. *The American Journal on Addictions, 21(5),* 416-423.

Grant, B.F., Hasin, D.S., Chou, S.P., Stinson, F.S., & Dawson, D.A. (2004). Nicotine dependence and psychiatric disorders in the United States: results from the national epidemiologic survey on alcohol and related conditions. *Archives of General Psychiatry, 61(11),* 1107-1115.

Hall, S.M., & Prochaska, J.J. (2009). Treatment of smokers with co-occurring disorders: emphasis on integration in mental health and addiction treatment settings. *Annual Review of Clinical Psychology, 5,* 409-431.

Hall, S.M., Tsoh, J.Y., Prochaska, J.J., Eisendrath, S., Rossi, J.S., Redding, C.A., Rosen, A.B., Meisner, M., Humfleet, G.L., & Gorecki, J.A. (2006). Treatment for cigarette smoking among depressed mental health outpatients: a randomized clinical trial. *American Journal of Public Health, 96(10),* 1808-1814.

Hatsukami, D., Kotlyar, M., Hertsgaard, L. A., Zhang, Y., Carmella, S. G., Jensen, J. A., ... Hecht, S. S. (2010). Reduced nicotine content cigarettes: Effects on toxicant exposure, dependence and cessation. *Addiction, 105,* 343-55.

Heatherton, T. F., Kozlowski, L. T., Frecker, R. C., & Fagerström, K. O. (1991). The Fagerström Test for Nicotine Dependence: A revision of the Fagerström Tolerance Questionnaire. *British Journal of Addictions, 86,* 1119-1127.

Heishman, S. J. (1999, September). Behavioral and cognitive effects of smoking: Relationship to nicotine addiction. *Nicotine & Tobacco Research, 1*(*Suppl* 2), S143–S147.

Heishman, S.J., Kleykamp, B.A., & Singleton, E.G. (2010). Meta-analysis of the acute effects of nicotine and smoking on human performance. *Psychopharmacology, 210*, 453–469.

Herning, R.I, Jones, R.T., Bachman, J., & Mines, A.H. (1981). Puff volume increases when low-nicotine cigarettes are smoked. *British Medical Journal (Clinical Research Ed), 283,* 187-189.

Hughes, J. R., & Hatsukami, D. K. (1986). Signs and symptoms of tobacco withdrawal. *Archives of General Psychiatry, 43,* 289-294. Hughes, J. R., & Hatsukami, D. K. (1998). Errors in using tobacco withdrawal scales. *Tobacco Control, 7,* 92-93.

Kleykamp, B. A., Jennings, J. M., Blank, M. D., & Eissenberg, T. (2005). The Effects of Nicotine on Attention and Working Memory in Never-Smokers. *Psychology of Addictive Behaviors, 19(4),* 433–438.

Kroenke, K., Spitzer, R. L., & Williams, J. B. (2001). The PHQ-9: Validity of a brief depression severity measure. *Journal of General Internal Medicine, 16,* 606-613.

Kroenke, K., Spitzer, R.L., Williams, J.B.W., & Lowe, B. (2009). An Ultra-Brief Screening Scale for Anxiety and Depression: The PHQ–4. *Psychosomatics, 50(6),* 613-621.

Lasser, K., Boyd, J.W., Woolhandler, S., Himmelstein, D.U., McCormick, D., & Bor, D.H. (2000). Smoking and Mental Illness: A Population-Based Prevalence Study. *Journal of the American Medical Association, 284(20),* 2606-2610.

Lawrence, D., Considine, J., Miltrou, F., & Zubrick, S.R. (2010). Anxiety disorders and cigarette smoking: Results from the Australian Survey of Mental Health and Wellbeing. *Australian and New Zealand Journal of Psychiatry, 44(6),* 520-527.

Levin, E.D., McClernon, F.J., & Rezvani, A.H. (2006). Nicotinic effects on cognitive function: behavioral characterization, pharmacological specification, and anatomic localization. *Psychopharmacology, 184,* 523–539.

Logan, G. D., Cowan, W. B., & Davis, K. A. (1984). On the ability to inhibit simple and choice reaction time responses: A model and a method. *Journal of Experimental Psychology: Human Perception and Performance, 10(2),* 276–291.

MacKillop, J., Murphy, J. G., Ray, L. A., Eisenberg, D. T., Lisman, S. A., Lum, J. K., & Wilson, D. S. (2008). Further validation of a cigarette purchase task for assessing the relative reinforcing efficacy of nicotine in college smokers. *Experimental and Clinical Psychopharmacology, 16,* 57-65.

Malpass, D., & Higgs, S. (2007). Acute psychomotor, subjective and physiological responses to smoking in depressed outpatient smokers and matched controls. *Psychopharmacology, 190,* 363–372.

McFall, M., Atkins, D.C., Yoshimoto, D., Thompson, C.E., Kanter, E., Malte, C.A., & Saxon, A.J. (2006). Integrating Tobacco Cessation Treatment into Mental Health Care for Patients with Posttraumatic Stress Disorder. *American Journal on Addictions, 15*, 336-344.

Myers, C. S., Taylor, R. C., Moolchan, E. T., & Heishman, S. J. (2008). Dose-related enhancement of mood and cognition in smokers administered nicotine nasal spray. *Neuropsychopharmacology, 33(3),* 588-598.

Nestor, L., McCabe, E., Jones, J., Clancy, L., & Garavan, H. (2011). Differences in "bottom-up" and "top-down" neural activity in current and former cigarette smokers: Evidence for neural substrates which may promote nicotine abstinence through increased cognitive control. *Neuroimage, 56,* 2258-2275.

Norman, S. B., Hami Cissell, S., Means-Christensen, A. J., & Stein, M. B. (2006). Development and validation of an overall anxiety severity and impairment scale (OASIS). *Depression and Anxiety, 23(4),* 245-249.

Piper, M. E., McCarthy, D. E., Bolt, D. M., Smith, S. S., Lerman, C., Benowitz, N., ... Baker, T. B. (2008). Assessing dimensions of nicotine dependence: An evaluation of the Nicotine Dependence Syndrome Scale (NDSS) and the Wisconsin Inventory of Smoking Dependence Motives (WISDM). *Nicotine & Tobacco Research, 10,* 1009-1020.

Poorthuis, R. B., Goriounova, N. A., Couey, J. J., & Mansvelder, H. D. (2009). Nicotinic actions on neuronal networks for cognition: general principles and long-term consequences. *Biochemical Pharmacology, 78(7),* 668-676.

Prochaska, J. J., Rossi, J. S., Redding, C. A., Rosen, A. B., Tsoh, J. Y., Humfleet, G. L., ... & Hall, S. M. (2004). Depressed smokers and stage of change: implications for treatment interventions. *Drug and Alcohol Dependence, 76(2),* 143-151.

Robinson, J. C., & Forbes, W.F. (1975) The Role of Carbon Monoxide in Cigarette Smoking. *Archives of Environmental Health. 30(9),* 425-434.

Rycroft, N., Hutton, S. B., & Rusted, J. M. (2006). The antisaccade task as an index of sustained goal activation in working memory: modulation by nicotine. *Psychopharmacology, 188(4),* 521-529.

Sheehan, D.V., Lecrubier, Y., Sheehan, K.H., Amorim, P., Janavs, J., Weiller, E., … Dunber, G.C. (1998). The Mini-International Neuropsychiatric Interview (M.I.N.I.): the development and validation of a structured diagnostic psychiatric interview for DSM-IV and ICD-10. Journal of Clinical Psychiatry. 59(Suppl 20), 22–57.

Sheehan, D. V., Lecrubier, Y., Sheehan, K. H., Amorim, P., Janavs, J., Weiller, E., ... Dunbar, G. C. (1997). The validity of the Mini International Neuropsychiatric Interview (MINI) according to the SCID-P and its reliability. *European Psychiatry, 12,* 232-241.

Sirota, A. D., Rohsenow, D. J., Dolan, S. L., Martin, R. A., & Kahler, C. W. (2013). Intolerance for discomfort among smokers: Comparison of smoking-specific and non-specific measures to smoking history and patterns. *Addictive Behaviors, 38(3),* 1782-1787.

Smith, P. H., Homish, G. G., Giovino, G. A., & Kozlowski, L. T. (2014). Cigarette smoking and mental illness: a study of nicotine withdrawal. *American Journal of Public Health, 104(2),* 127-133.

Spitzer, R. L., Kroenke, K., Williams, J. B., & Löwe, B. (2006). A brief measure for assessing generalized anxiety disorder: the GAD-7. *Archives of Internal Medicine, 166(10),* 1092-1097.

Strasser, A. A., Lerman, C., Sanborn, P. M., Pickworth, W. B., & Feldman, E. A. (2007). New lower nicotine cigarettes can produce compensatory smoking and increased carbon monoxide exposure. *Drug and Alcohol Dependence, 86,* 294-300.

Stroop, J. R. (1935). Studies of interference in serial verbal reactions. *Journal of Experimental Psychology, 18(6)*:643–662.

Taylor, G., Mcneill, A., Girling, A., Farley, A., Lindson-Hawley, N., and Aveyard, P. (2014). Change in mental health after smoking cessation: systematic review and meta-analysis. *British Medical Journal, 348,* g1151.

Tengs, T.O., Ahmad, S., Savage, J.M., Moore, R., Gage, E. (2005). The AMA proposal to mandate nicotine reduction in cigarettes: a simulation of the population health impacts. *Preventive Medicine, 40,* 170-80.

Thorsteinsson, H. S., Gillin, J. C., Patten, C. A., Golshan, S., Sutton, L. D., Drummond, S., Clark, C. P., Kelsoe, J., and Rapaport, M. (2001). The effects of transdermal nicotine therapy for smoking cessation on depressive symptoms in patients with major depression. *Neuropsychopharmacology, 24(4),* 350.

Tiffany, S. T., & Drobes, D. J. (1991). The *development* and initial validation of a questionnaire on smoking urges. *British Journal of Addiction, 86,* 1467-1476.

Tsoh, J. Y., Humfleet, G. L., Muñoz, R. F., Reus, V. I., Hartz, D. T., and Hall, S. M. (2000). Development of major depression after treatment for smoking cessation. *The American Journal of Psychiatry, 157(3),* 368.

Watson, D., Clark, L. A., and Tellegen, A. (1988). Development and validation of brief measures of positive and negative affect: the PANAS scales. *Journal of Personality and Social Psychology, 54(6),* 1063.

Weinberger, A. H., Desai, R. A., and Mckee, S. A. (2010). Nicotine withdrawal in u.s. smokers with current mood, anxiety, alcohol use, and substance use disorders. *Drug and Alcohol Dependence, 108(1-2),* 7–12.

Westman, E., Levin, E., & Rose, J. (1992). Smoking while wearing the nicotine patch: Is smoking satisfying or harmful? *Clinical Research, 40,* 871A.

Ziedonis, D., Hitsman, B., Beckham, J. C., Zvolensky, M., Adler, L. E., Audrain-McGovern, J., Breslau, N., Brown, R. A., George, T. P., Williams, J., Calhoun, P. S., and Riley, W. T. (2008). Tobacco use and cessation in psychiatric disorders: National institute of mental halth report. *Nicotine Tobacco Research, 10(12),* 1691–1715.

STUDY PROTOCOL: SMOKERS WITH OPIOID USE DISORDER

Table of Contents

Objective: 49
Background Information 49
Importance of Evaluating VLNC Cigarettes in Smokers with OUD: 50
Cigarettes to Be Assessed in This Study 50
Screening Procedures 51
______Recruitment 51
______Informed Consent Process 51
______Screening Measures/Assesments and Physiological Samples to be Collected 51
______Suicidality/Mental Health Monitoring 53
______Includion/Exclusion Criters 54
______Eligibility Determination 56
Baseline Procedures 57
______Measures/Assesments and Physiological Samples to be Collected 57
______Cognitive Tasks 59
______Smoking Topography 60
______Interactive Voice Response 60
______Description of Biological Specimens 60
______Biomarker Shipping and Storage 61
______fMRI Testing 61
Experimental Procedures 62
______Experimental Period 62
______Visit Scheduling Requirements 62
______Experimental Visits Weeks 1, 3, 5, 7, 9 and 11 Procedures 62
_________Measures/Assessments 13
______Experimental Visits Weeks 2, 4, 6, 8, 10 and 12 Procedures 63
_________Measures/Assessments 63
__________ Week 12 fMRI Testing 64
______Interactive Voice Response System 64
______Variable Incentive Program 65
______Product and Procesures Compliance Review Sessions 66
______Quit Attempts During the Study Protocol 66
_________If a Participant is Currently Abstaining from Smoking with the Intention to Quit 66
_________If a Participant is Planning to Quit Smoking, But Has Not Initiated Quit Attempt 66
______Abstinence Assessment Session 67

Participants Who Meet Criteria for Abstinence ... 67
Measures/Assessments ... 67
Additional Tasks ... 68
Participants Who Do Not Meet Criteria for Abstinence ... 68
Measures/Assessments ... 68
Participant Compensation ... 68
End of Study ... 69
30 Day Follow up Phone Call ... 69
Randomization ... 69
Product Accountability ... 71
Statistical Methods and Sample Size ... 72
Potential Risks of Participation ... 72
Risks of Participation ... 72
Avoiding Risk to the Fetus ... 74
Expected Benefits of Participation ... 74
Study Debriefing ... 74
Protection Against Risk ... 74
Data Collection Protections ... 74
Data Storge ... 75
Adverse Events ... 75
Withdrawal or Monitoring of Participants ... 76
Data Safety Monitoring Board ... 77
Investigational Tobacco Product ... 78
Certificate of Confidentiality ... 78
Outcome Variables ... 79
References ... 80

Abbreviations

- VLNC: Very low nicotine content
- RNC: Reduced nicotine content
- NNC: Normal nicotine content
- CPD: Cigarettes per day
- CO: Carbon monoxide
- BAL: Breath alcohol levels
- BP: Blood pressure
- HR: Heart rate
- BPM: Beats per minute
- BMI: Body Mass Index
- NMR: Nicotine metabolite ratio
- NNN: *N′*-nitrosonornicotine
- NNAL: 4-(methylnitrosamino)-1-(3-pyridyl)-1-butanol
- BDI: Beck's Depression Inventory
- OASIS: Overall Anxiety Severity and Impairment Scale
- MINI: Mini International Neuropsychiatric Interview
- ASI: Addiction Severity Index
- FTND: Fagerström Test for Nicotine Dependence
- WISDM: Wisconsin Index of Smoking Dependence Motives
- TLFB: Timeline Follow Back
- MNWS: Minnesota Nicotine Withdrawal Scale
- QSU: Questionnaire of Smoking Urges
- CES: Cigarette Evaluation Scale
- CPT: Continuous Performance Task
- IVR: Interactive Voice Response
- EDC: Electronic Data Capture
- CPT: Cigarette Purchase Task
- Brief-A: Behavioral Rating Inventory of Executive Function
- EQ-5D: Euro-Qol
- TPQ: Time Perspectives Questionnaire
- D-KEFS: Delis-Kaplan Executive Function System

- DDT: Delayed Discounting Task
- WASI-II: Wechsler Abbreviated Scale of Intelligence-II
- SST: Stop Signal Task
- FeNO: Fractional Exhaled Nitric Oxide
- 3 HC: 3-hydroxycotinine
- COT: Cotinine

Protocol

Objective:

The primary overall objective of this study is to evaluate the effects of extended exposure to cigarettes differing in nicotine content in opioid-maintained smokers using a 3-condition, parallel groups design. After a baseline period in which daily smoking rate and other baseline assessments are completed, participants will be randomly assigned to one of three cigarette conditions (0.04, 2.4 mg, and 15.8 mg nicotine/gram of tobacco) for the 12-week experimental period.

Background Information:

The 2009 Family Smoking Prevention and Tobacco Control Act (FSPTCA) gives the Food and Drug Administration (FDA) regulatory authority over tobacco products, including nicotine levels in cigarettes. That is an exciting development as it creates the opportunity to examine the Benowitz and Henningfield (1994) hypothesis that smoking prevalence, nicotine dependence, and smoking-related morbidity and mortality can be lowered substantially by reducing the nicotine content of cigarettes to non-addictive levels. Computer modeling predicts that reducing nicotine levels in cigarettes would produce substantial improvements in population health (Tengs et al., 2005). An essential initial step towards the implementation of such a policy is to thoroughly investigate its safety and potential unintended adverse consequences. Indeed, the FDA's Center for Tobacco Products seeks to establish research centers to assist with the mission of investigating such regulatory matters related to the FSPTCA (see RFA-DA-13-003). The FDA explicitly notes that researching tobacco regulatory questions in vulnerable populations is a crosscutting agency priority, listing opioid-dependent adults among the vulnerable populations of interest.

Prevalence of smoking among opioid-dependent adults far exceeds that of the general US adult population (84-94% *vs.* 20%, respectively) (Clemmey et al., 1997; Nahvi et al., 2006; Richter et al., 2001; SAMHSA, 2007). Opioid-dependent smokers are also at elevated risk for smoking-related adverse health effects. Smoking in this group is associated with significant morbidity and mortality (Engstrom et al., 1991; Hser et al., 1994; Hurt et al., 1996), with the mortality rate of opioid-dependent smokers estimated at four-fold that of opioid-dependent nonsmokers (Hser et al., 1994), and individuals with substance use disorders more likely to die of tobacco-related disorders such as lung and larynx cancer and respiratory disease than the general population (Grinshpoon et al., 2011). In addition to these direct adverse health consequences of smoking, opioid-dependent smokers also present with additional unique risk factors related to their opioid dependence that may further increase their risk. First, opioid-maintained patients are already at elevated risk for adverse cardiac effects. Methadone is a potent blocker of the delayed rectifier potassium ion channel. Chronic administration has cardiac toxicity and arrhythogenic potential and can produce QT-prolongation and Torsades de Pointes (polymorphic ventricular tachycardia) in susceptible patients (Andrews et al., 2009; George et al., 2008; Justo et al., 2006; Huh & Park, 2010; Modesto-Lowe et al., 2010; Roy et al., 2012; Stringer et al., 2009; Wallner et al., 2008). Second, acute and chronic opioid administration is also associated with weight gain, glycemic dysregulation, and dental pathology. A recent review by Mysels and Sullivan (2010), for example, found that activation of the mu-opioid receptor is associated with increased sweet, or palatable, taste preference, hyperglycemia induced by direct action on

pancreatic islet cells and potential insulin resistance caused by dietary preference for sugary foods. Increased preference for and ingestion of sweet foods is associated with weight gain and tooth decay. The authors concluded that methadone-maintained patients are especially susceptible to weight gain and diabetes, and have poor follow-up with primary care treatment, thereby making them a population with multiple vulnerabilities. Finally, opioid-dependent patients demonstrate generally poor adherence to medication regimens, which likely interferes with their ability to take advantage of available pharmacotherapies for smoking cessation. For example, in a 12-week trial investigating the efficacy of bupropion plus nicotine replacement therapy (NRT) on smoking cessation among methadone-maintained smokers, 53% reported using their bupropion less often than prescribed (Richter et al., 2005). In a subsequent randomized trial investigating the efficacy of NRT on smoking cessation among methadone patients, only 34% of participants in that study used their assigned NRT (i.e., transdermal patch) through the end of the study (Reid et al., 2008). In both studies, suboptimal pharmacotherapy adherence likely contributed to the poor cessation rates observed. Taken together, in addition to the direct adverse consequences associated with smoking alone, combined use of tobacco and opioids may produce additive or even synergistic increases in risk for adverse health effects from tobacco use in this population.

Importance of Evaluating VLNC Cigarettes in this Vulnerable Group of Smokers:
The studies summarized in the prior section provide compelling evidence that VLNC cigarettes can substitute for usual brand cigarettes and that extended exposure to VLNC cigarettes may reduce smoking rate, toxicant exposure, and severity of nicotine dependence. These findings underscore the tremendous potential this innovative public policy strategy has for reducing smoking prevalence and smoking-related disease and death in the US. However, a serious limitation of these studies that is directly relevant to this proposal is that they uniformly excluded vulnerable populations. This is an important gap in knowledge that must be addressed to comprehensively evaluate the Benowitz and Henningfield hypothesis. Understanding how smokers with substance use disorders (SUDs) and other vulnerabilities to smoking and smoking-related problems respond to reduced-nicotine cigarettes is essential for evaluating the potential impact of a nicotine reduction policy. This project represents the first investigation of reduced-nicotine cigarettes in opioid-dependent smokers and stands to contribute new scientific information with the potential to directly inform FDA policy decisions.

Cigarettes to be assessed in this study:
The cigarettes to be used in this study were made under an NIH contract with production being overseen by the Research Triangle Institute (referred to as “Spectrum cigarettes”). NIH currently has approximately 10 million of these cigarettes (of varying types) for research purposes. The cigarettes selected for the study span the range of yields likely to produce the hypothesized effects, as described above. Spectrum cigarettes are not currently commercially available, although they are similar in many ways to marketed cigarettes (e.g., similar manufacturing, filter, paper, etc.).

Screening Procedures

Recruitment:

A sample size of 207 completers is proposed to test the primary outcome. Anticipating 25% attrition, and six pilot participants (3 at UVM, 3 at JHU), 282 participants will be enrolled across both sites (188 at UVM, 94 at JHU). Potential participants will respond to community advertisements (local newspapers, community bulletin boards, lab Facebook page, Facebook ads, lab website, center website, Craigslist, city buses, etc.) that contain a study description, link to an online survey and the name and phone number of the Research Assistant. Participants can choose to complete the pre-screening questionnaire online or by phone. If deemed eligible, those who complete the online questionnaire will be called by the Research Assistant to further discuss the study. The RA will read a script briefly explaining the study. Participants will be informed that this is not a smoking cessation program, and that smoking cessation services are available in the community independent of their decision to participate in this study. If interested, they will be scheduled for an in-person screening interview. Those who call into the laboratory will be read a script briefly explaining the study. After verbal informed consent is received, the participants will be asked questions over the phone to determine initial eligibility. Callers will be informed that this is not a smoking cessation program, and that smoking cessation services are available in the community independent of their decision to participate in this study. If eligible and interested, they will be scheduled for an in-person screening interview.

Potential participants will be instructed to bring a pack of their usual brand cigarettes, all prescription medications they are currently taking and identification (example, driver's license) to the screening visit. If participants anticipate not having acceptable ID site staff should consult with the project coordinator or study PI.

A participant must complete his/her in-person screening session within 30 days of completing the pre-screening questionnaire. If the participant is not able to attend the in-person screening visit in that timeframe, he/she will need to complete the pre-screening questionnaire again.

Informed Consent Process:

Before beginning the informed consent process, potential participants will need to produce identification as described above. The interviewer will confirm the age and identity of the participant. If the participant is not between the ages of 18 and 70, he/she will be dismissed without payment. During the in-person screening session, study information will be presented and written informed consent will be required prior to participating in the screening session. In order to ensure adequate informed consent, participants will be asked to read the first several lines aloud (to determine literacy) and will then be given ample time to read the consent document. If the interviewer suspects the participant is not literate, he or she will have them continue reading further to confirm. Inability to read and comprehend written study materials will result in ineligibility and the interviewer will inform the participant that they are not eligible. Only after the participant and the researcher are fully satisfied that the participant understands the purpose of the study, the confidentiality of the data, the procedures, the risks/benefits and his/her rights as a research participant will the consent form be signed and the participant undergo screening procedures.

Screening Measures

Those who consent will be screened for eligibility using the following measures:

The following physiological measures will be collected, recorded on paper, and entered into REDCap by the interviewer at the end of the visit:

1) Breath alcohol levels (BAL) will be measured using an Alcosensor monitor. Participants with levels over 0.01 g/l may reschedule the interview but will need to be re-consented to ensure they have received adequate informed consent. They will be excluded if they are positive the second time.
2) Weight and height will be measured to determine the participant's Body Mass Index. Weight will be measured in kilograms and height will be measured in centimeters.
3) Expired breath carbon monoxide (CO) levels will be assessed using a Smokerlyzer ED50 CO meter (Bedfont Instruments), a reliable and valid measure of recent smoking.
   a. NicAlert Strips will be used to asses urinary cotinine levels if a participant's carbon monoxide reading is less than or equal to 8 ppm.
4) A urine toxicological screen will be performed to assess the presence of illicit drugs including marijuana, cocaine, opiates, oxycodone, benzodiazepines, barbiturates, amphetamines, methadone, buprenorphine, methamphetamines, MDMA and PCP. Participants who fail the drug screen for drugs other than marijuana or their prescribed opioid medication may reschedule the interview but will need to be re-consented to ensure they have received adequate informed consent. They will be excluded if they are positive for drugs (other than marijuana or prescribed opioid medication) the second time.
5) Urine Pregnancy Test (HCG detection) will be performed for all participants.
6) Blood pressure and heart rate will be measured using a CritiCare monitor to help the licensed medical professional determine final participant eligibility.

The following screening questionnaires will be participant-administered via paper and then will be entered into REDCap by the interviewer at the end of the visit:

1) Identifying Information Form will include the participant's REDCap Subject Identifier, name, address (including the county of residence), email address, phone number, age, date of birth, and social security number (if applicable).
   a. This form will be entered into the 'Identifying Information Access Database'.
      i. Each site will have a separate 'Identifying Information Access Database'.
      ii. Identifying information will not be shared with other sites. Each site is responsible for maintaining confidentiality of this information.
      iii. Identifying information will be kept in a locked file cabinet (source document) and in a password protected Access Database (electronic version) separate from all other study data.
2) Beck Depression Inventory (BDI; Beck, Ward, & Mendelson, 1961), to assess depressive symptoms.
3) Overall Anxiety Severity and Impairment Scale (OASIS; Norman et al., 2006) to assess frequency and severity of anxiety symptoms.

The following screening assessments will be administered as an interview and then will be entered into REDCap by the interviewer at the end of the visit:

1) The Mini International Neuropsychiatric Interview (MINI) suicide subscale (Sheehan et al., 1997) to evaluate suicide risk.

2) The Mini International Neuropsychiatric Interview (MINI) PLUS 6.0 Modules
3) MINI Follow-up Questionnaire (if applicable)
4) Tobacco Use History and Exposure Questionnaire, which measures variables such as smoking amount, cigarette brand, age of initiation of smoking, number of quit attempts, duration of quit attempts and duration of smoking.
5) Smoking Cessation Therapy Use Questionnaire
6) Time Since Last Cigarette Questionnaire
7) Maintenance Drug Dose Questionnaire – Screening Version
8) Medical History Questionnaire to assess current diagnoses, symptoms and past health problems.
    a. The medications section will be transferred onto the 'Concomitant Medications' form and entered into REDCap.

The following screening assessments will be completed by the participant directly in REDCap, except where noted:

1) Demographic History Questionnaire, which will assess age, gender, ethnicity, race, education, income, marital status, and employment history.
2) Alcohol Use Questionnaire (12 month and 1 month version)
3) Drug Use Questionnaire (12 month and 1 month version)
4) Fagerström Test for Nicotine Dependence (FTND; Heatherton et al., 1991)
5) Wisconsin Inventory of Smoking Dependence Motives-Brief Scale (WISDM; Piper et al., 2008), will be administered to assess nicotine dependence severity.
6) Smoking Stages of Change Algorithm as well as a contemplation ladder to assess intention to quit smoking (DiClemente et al., 1991).
7) Addiction Severity Index-MV (ASI; McLellan et al., 1985) for the assessment of substance use-related problems.
    a. Will be completed by participant directly in ASI-MV Connect system.

8) The Mini International Neuropsychiatric Interview (MINI 6.0) (Sheehan et al., 1990) a structured diagnostic interview to evaluate psychiatric disorders.
    a. Will be completed by participant through the In-Home Screening system supported by Medical Outcomes Systems.

In the event that the REDCap website is not functioning, the assessments will be printed out and administered on paper. The source documents will be kept in the participant's binder. The interviewer will enter the data into REDCap when it resumes functioning properly. This information should be recorded in the 'End of Visit Evaluation Form' and filed in the participant's binder.

Suicidality/Mental Health Monitoring
Participants who endorse any suicidal ideation questions, indicate suicidal intention in the past month or a suicide attempt in the past 6 months as indicated on the BDI (score > 0 on question 9) or MINI suicide subscale (endorse question 3, 4 and/or 5 on the MINI suicide subscale or question 6 on the MINI suicide subscale with suicide attempt in the past 6 months) or answer "yes" to question A3g on the MINI Neuropsychiatric interview and symptoms have occurred in the past two weeks, will not be eligible to participate in the study. The research staff member will contact a licensed on-site clinician for evaluation. In the event that no clinician is available, staff will put the participant in contact with the National Suicide Prevention Lifeline at 1-800-273-

8255. They will also contact the Study Coordinator and Site PI to inform them of the situation as soon as possible. Additionally, they will contact the Project Coordinator to inform her of the situation. The participant will be paid $25 (+$25 bonus is applicable) and provided with local mental health resources. Post enrollment, any report of suicidal ideation or attempt by a participant will be grounds for immediate withdrawal from the study.

Inclusion/Exclusion Criteria

Inclusion Criteria:

1) Men and women ages 18-70, who are currently receiving methadone or buprenorphine maintenance treatment for opioid dependence,
2) Report smoking ≥ 5 cigarettes per day for the past year,
3) Provide an intake breath CO sample >8 ppm, (if ≤ 8 ppm, then NicAlert Strip > 2)
4) Be without current (within the past year) serious mental disorder that would interfere with study results or completion as determined by the licensed medical professional or PI,
5) Be sufficiently literate to complete the research-related tasks,
6) Be in good physical health without serious illness or change in health or medication (not including methadone or buprenorphine dose) in the past three months as determined by the license medical professional at each site,
7) Not pregnant or nursing, and report using oral, implant, patch, ring, IUD, injection or barrier contraceptives or report being surgically sterile, or post-menopausal,
8) Report no significant use of other tobacco or nicotine products within the past month (more than 9 days in the past 30) and,
9) Participants must be maintained on a stable methadone or buprenorphine dose for the past month, with no evidence of regular illicit-drug abuse (<30% positive specimens in the past 30 days).
    a. Consent to confirm dose and drug abstinence with the participant's opioid clinic will be obtained at screening and we will monitor any changes in dose throughout the study.
    b. Participants must provide at least three urine samples within the last 30 days. If they do not have three they will be asked to come in and provide a sample. They may leave up to two samples per week with at least one full day between samples.

Exclusion Criteria:

1) Any prior regular use (used as primary cigarette outside of laboratory) of Spectrum cigarettes (i.e., research cigarettes with reduced nicotine content),
2) Exclusive use of roll-your-own cigarettes,
3) Planning to quit smoking in the next 30 days,
4) A quit attempt in the past 30 days resulting in greater than 3 days of abstinence,
5) Currently taking anticonvulsant medications including:
    a. Phenytoin [Brand Name: Dilantin]
    b. Carbamazepine [Brand Name: Tegretol, Carbatrol, Equetro, Epitol]
    c. Oxcarbazepine [Brand Name: Trileptal]
    d. Primidone [Brand Name: Mysoline]
    e. Phenobarbital

6) Positive toxicology screen for any of the following drugs: cocaine, illicit opiates, oxycodone, benzodiazepines, barbiturates, amphetamines, methamphetamines, MDMA and PCP
    a. Marijuana will be tested for but will not be an exclusionary criterion. Participants will be discouraged from smoking marijuana during the study.
    b. Participants with valid prescriptions for opiates, benzodiazepines, barbiturates or amphetamines will not necessarily be excluded.
    c. Participants failing the toxicology screen will be allowed to re-screen once. These participants will need to be re-consented before being rescreened to ensure they have received adequate informed consent.
7) Not currently enrolled in a treatment program for opioid dependence and/or not currently stable on their methadone or buprenorphine dose,
8) Breath alcohol level > 0.01
    a. Participants failing the breath alcohol screen will be allowed to re-screen once. These participants will need to be re-consented before being rescreened to ensure they have received adequate informed consent.
9) Self-report of binge drinking alcohol (more than 9 days in the past 30 days, 4/5 drinks in a 2 hour period in females/males),
10) Systolic blood pressure < 90 or ≥ 160 mmHg
    a. Participants failing for blood pressure will be allowed to re-screen once.
11) Diastolic blood pressure < 50 or ≥ 100 mmHg
    a. Participants failing for blood pressure will be allowed to re-screen once.
12) Breath CO > 80 ppm,
13) Heart rate is greater than or equal to 115 bpm or less than 45 bpm
    a. Participants failing for heart rate will be allowed to re-screen once.
14) Currently seeking treatment for smoking cessation,
15) Have used nicotine replacement, bupropion or other pharmacotherapies as cessation aids in the past month (bupropion will be allowed for treatment of depression),
16) Current symptoms of psychosis, dementia or mania,
17) Suicidal ideation in the past month (score > 0 on the BDI question 9 or endorse question 3, 4 and/or 5 on the MINI suicide subscale),
18) Answer "yes" to question A3g on the MINI Neuropsychiatric Interview Major Depressive Episode Module and symptoms occurred within the past two weeks,
19) Suicide attempt in past 6 months (endorse question 6 on the MINI suicide subscale with suicide attempt in the past 6 months) or,
20) Participation in another research study in the past 30 days.
21) Co- habitation with any former research participant who was provided with Spectrum research cigarettes to smoke outside the lab

Children under age 18 are excluded because they cannot legally buy cigarettes. Those with unstable medical, psychiatric, or medication conditions (as determined by the licensed medical professional) are excluded as these symptoms could affect a participant's ability to complete the study. Examples include but are not limited to the following: angina, stroke, heart attack which occurred since phone screening, blood clots in the arms or legs for which the individual is undergoing active medical treatment, cancer requiring active chemotherapy or radiation therapy, severe shortness of breath caused by conditions such as uncontrolled asthma, COPD, or arrhythmia, active untreated infection such as pneumonia, active untreated endocrine disorder such as hyperthyroidism. We will exclude those currently seeking smoking treatment and those who plan to quit in the next 30 days, as participation in this study may not lead to reductions in smoking. We will exclude pregnant or nursing women and women of reproductive potential who are unwilling to use acceptable forms of birth control throughout the study. We will also exclude

anyone with current or recent alcohol or drug abuse problems as these factors could independently affect smoking behavior during the study. Individuals with baseline CO readings greater than 80 ppm, those with heart rate or blood pressure readings that are out of range (systolic: 90-159 mmHg; diastolic: 50-99 mmHg; HR: 45-114 bpm) and anyone who has attempted suicide in the past six months will be excluded from the study for safety concerns. Individuals who smoke ‘roll your own’ cigarettes exclusively will be excluded from the study because we will be unable to standardize their baseline smoking behavior. Individuals who have recently participated in a research study will be excluded as participation may have changed their smoking behavior, which may preclude a stable smoking baseline. Because participants are required to complete portions of the protocol independently, they will need to be able to independently read and comprehend the study materials.

Eligibility Determination:

The research assistant will review the entire screening assessment battery for initial eligibility determination, confirming the subject meets the above described inclusion/exclusion criteria. The final eligibility of the participant will be determined by a licensed medical professional (MD, DO, NP, PA, Master’s prepared RN or CRN) at each site after reviewing the Medical History Questionnaire, BDI, Mini Neuropsychiatric Interview, and the MINI suicide subscale. The licensed medical professional may meet with a participant if available and think it necessary for eligibility determination. He/she will sign off on eligibility prior to the first baseline visit. If the licensed medical professional determines the participant is not medically eligible to participate in the study, has current symptomatology that would interfere with interpretation of the data or is unlikely to complete the study he/she will inform the research assistants who will contact the participant prior to the first baseline visit. The licensed medical professional will not need to review the medical history forms of participants who are not eligible for other, non-medical reasons.

If a participant fails the urine toxicology screen due to a prescription medication he/she is taking, then he/she will not be automatically excluded. The interviewer will make note of this when he/she submits the forms to the licensed medical professional for final eligibility determination.

Once all the screening procedures have been completed, researchers will pay participants $25 (+$25 bonus if applicable) for their time as long as they pass the drug and breath alcohol tests and meet the minimum requirements for carbon monoxide or NicAlert levels. Those participants who do not pass these tests or meet these requirements will be dismissed from the study without payment. Marijuana will be tested for but will not be an exclusionary criterion. If a participant does not pass the drug test but has a current, valid prescription that would explain the failed test he/she will not be automatically excluded and will still receive the visit payment. Participants who meet all other eligibility criteria, sans the medical criteria, will be scheduled for the first baseline visit.

At the end of the screening session, the researcher will complete the End of Visit Evaluation Form, which will be filed in the subject’s binder. This will allow the researcher to make note of any problems encountered during the visit, to track which computers were used for which tasks, and to assess the truthfulness of the participant in regards to self-report of tobacco use.

Baseline Procedures

This study will use a one-week, two-session baseline period to collect baseline individual difference measures and monitor daily usual-brand smoking behavior. At Baseline 1, participants will be provided their usual brand cigarettes to smoke, equivalent to 150% of their daily smoking rate. A time line follow back (TLFB) will be used to assess the daily cigarette use for the past 7 days. Participants will be provided their usual brand cigarettes for the first seven days of the baseline period. If the baseline period extends past seven days, participants will need to purchase their own usual brand cigarettes. Use of a two session baseline period will ensure stability of daily smoking reports, reduce reactivity to the daily cigarette monitoring, and reduce participant burden. During the two baseline sessions, participants will complete subjective questionnaires, assessments of cognitive functioning, and smoking topography. Each visit will last approximately two to four hours. At the end of each baseline session, the researcher will complete the End of Visit Evaluation Form, which will be filed in the participants' binder. This will allow the researcher to make note of any problems encountered during the visit, to track which computers were used for which tasks, and to assess the truthfulness of the participant in regards to self-report of tobacco use.

<u>Visit scheduling requirements for baseline period:</u>

Participants will be required to schedule the Baseline 1 visit within 30 days of their screening visit. If a participant still wants to be in the study after 30 days, he/she will need to be re-screened. The participant will need to be re-consented but will maintain the original REDCap Subject Identifier. The ideal target window separating Baseline 1 and Baseline 2 is between 7 and 12 days. The minimum is 7 days and the maximum is 21 days. If the participant does not complete the visit within 21 days, then he/she will not be rescheduled and will be discontinued from the study.

<u>Measures/Assessments</u>

Physiological measures collected at Baseline 1, recorded on paper, and entered into REDCap by the interviewer at the end of the visit:

1) BAL
2) Weight
3) CO
4) Blood Pressure
5) Heart Rate
6) Urine Toxicology

The following questionnaires will be participant-administered via paper at Baseline 1 and then will be entered into REDCap by the interviewer at the end of the visit:

1) BDI
2) OASIS

The following assessments will be administered as an interview at Baseline 1 and then entered into REDCap by the interviewer at the end of the visit:

1) Concomitant Medications Form
2) Health Changes Questionnaire, which will assess any weekly health changes,
3) Time Since Last Cigarette Questionnaire

The following assessments will be administered at Baseline 1 and completed by the participant directly in REDCap:

1) Perceived Health Risks Rating (Hatsukami et al., 2010), a measure of the perceived addictive potential and other health risks associated with cigarettes
2) Respiratory Health Questionnaire, a measure of cough, shortness of breath and other respiratory symptoms
3) Minnesota Nicotine Withdrawal Scale (MNWS; Hughes & Hatsukami, 1986), a measure of nicotine withdrawal
4) Questionnaire of Smoking Urges-brief scale - Usual Cigarette (QSU; Cox, Tiffany, & Christen, 2001; Tiffany & Drobes, 1991), which measures the urge to smoke
5) Cigarette Evaluation Scale – Usual Cigarette (CES; Westman, Levin, & Rose, 1992), which measures responses to cigarettes (e.g., reward, satisfaction).
6) Intolerance for Discomfort Questionnaire - (IDQ; Sirota et al., 2013), assesses intolerance for the discomfort of smoking abstinence. The measure includes three subscales: physical discomfort, emotional discomfort and smoking withdrawal discomfort.

10) Cigarette Purchase Task – Usual Brand Version (CPT; MacKillop et al., 2008), a self-report analogue of a progressive-ratio schedule that measures the relative reinforcing efficacy of cigarettes by querying how many of that day's cigarette they would consume in a day at varying prices. This task will indicate whether prolonged VLNC cigarette use reduces cigarette demand and increases sensitivity to increases in cigarette costs.
11) Perceived Stress Scale - 4 item (PSS-4; Cohen, Kamarck, & Mermelstein, 1983), which measures the degree to which life situations are appraised as stressful.
12) Positive and Negative Affect Schedule (PANAS; Watson, Clark, & Tellegan, 1988), which measures symptoms of positive and negative affect.

Physiological measures collected at Baseline 2, recorded on paper and entered into REDCap by the interviewer at the end of the visit:

8) BAL
9) Weight
10) CO
11) Blood Pressure
12) Heart Rate
13) Urine Toxicology
14) Urine Pregnancy

The following assessments will be administered as an interview at Baseline 2 and then entered into REDCap by the interviewer at the end of the visit:

1) Concomitant Medications Form
2) Health Changes Questionnaire
3) Time Since Last Cigarette Questionnaire

The following assessments will be administered at Baseline 2 and completed by the participant on paper and entered into REDCap by the interviewer at the end of the visit:

1) BDI
2) OASIS

The following assessments will be administered at Baseline 2 and completed by the participant directly in REDCap:

1) FTND
2) WISDM

In the event that the REDCap website is not functioning, the assessments will be printed out and administered on paper. The source documents will be kept in the participant's binder. The interviewer will enter the data into REDCap when it resumes functioning properly. This information should be recorded in the 'End of Visit Evaluation Form' and filed in the participant's binder.

Cognitive Tasks (Baseline 2 Only):
Cognitive functioning will be assessed using a battery of computer-based assessments. We will assess domains that are theoretically linked to smoking and likely to be sensitive to nicotine abstinence (Heishman, 1999; Kleykamp et al., 2005; Rycroft et al., 2006). Prior to test administration, participants will be trained to ensure their understanding of each test. Tests will be administered on a desktop computer.

6) **N-Back (0,2) Task** (Ernst et al., 2001): A measure of working memory in which participants view serially presented letters on a computer. They must indicate whether each letter presented is the same or different from the letter presented a specified number of positions back in the string of letters (e.g. 2-back).
7) **2-Letter Search** (Ernst et al., 2001): A measure of focused attention in which participants view strings of letters on a computer screen looking for whether each string contains or does not contain two target letters.
8) **Continuous Performance Test** (CPT; Myers et. Al., 2008): A measure of sustained attention, participants must monitor a string of stimuli (e.g. letters) serially presented on a computer screen monitoring for presentation of a target stimulus. The task is balanced so that they either must respond, or inhibit a response each time the target is presented.
9) **Stop Signal Task** (SST; Logan et al., 1984): A computer administered test of behavioral inhibition. Participants make frequent motor responses (e.g., left/right responses indicating if a visually presented arrow points left or right) and occasional, unpredictable response inhibitions (e.g., when a second arrow, pointing upwards, is presented). The stop signal delay (the interval between the onset of the go signal and stop signal) is

adjusted after each stop trial according to the participants' performance to achieve 50 percent inhibition success rate.

10) **Nicotine Stroop Task** (Stroop, 1935): Frequently used measure of inhibitory control functioning. It measures the ability to focus attention on relevant stimuli while ignoring distracters and to suppress a prepotent response (i.e., word reading) in favor of an atypical one (i.e., color naming). Participants will be shown a number of images. The images will either be nicotine related, evocative, or neutral in nature with different color borders (red, blue, green yellow). The participants will be asked to use response triggers to identify the color of the border for each picture as they appear on the screen.

Smoking Topography (Baseline 2 Only):

Puff Topography, a precise measure of smoking behavior (Brauer et al., 1996; Herning et al., 1981; Robinson & Forbes, 1975), will be used to examine whether prolonged use of the experimental cigarettes affects topography measures that may indicate smoking compensation (Strasser et al., 2007). Puff topography will be assessed using a CReSS pocket device that provides a valid measurement of puff number, puff volume, inter-puff interval and other indices (Blank et al., 2009). Carbon monoxide readings will be collected before and 15 minutes after puff topography. Participants will smoke one cigarette of their usual brand.

Interactive Voice Response System:

At the end of the first baseline visit, participants will be trained to use the Interactive Voice Response (IVR) System, which will contact participants each day throughout the study and ask about their smoking behavior as well as withdrawal symptoms the week before and after Baseline 2. We will also review the IVR adherence incentive program, which consists of $1 per call plus a $10 bonus for seven consecutive calls. Participants will be provided a study cell phone if they have unreliable telephone access, do not have enough monthly cell phone minutes or prefer not to use their own phone.

The IVR system is operated by TeleSage. To be enrolled in the IVR system, research staff will enter the participants initials, telephone number, subject identifier, and visit dates into the IVR TCORS website. Identifying information (initials and telephone numbers) will not be extracted with the data by the bioinformatics group. Please refer to TeleSage's privacy statement and HIPAA compliance form for additional information.

Baseline 2 biological specimens:

1) <u>Urine sample for smoking biomarker assessment</u>**:**
   Participants will be asked to bring a urine sample (first void of the day) to the second baseline session for biomarker assessment. Samples will be stored at temperatures no more than -80°C. The tobacco-specific carcinogen biomarkers are total NNAL and PAH. Anatabine and anabasine will be tested in the VLNC condition to validate abstinence or measure the extent of nicotine replacement therapy being used. Total cotinine levels will also be assessed to measure daily nicotine exposure. Participant's will be reminded with a phone call the day before the visit, those who forget will be asked to provide an onsite urine sample.
2) <u>Pulmonary Marker:</u>
   Fractional Exhaled Nitric Oxide (FeNO) will be assessed as a measure of lung function using the NIOX VERO, a hand-held device for exhaled NO analysis. FeNO involves no storing or shipping of specimens, rather, the participant will exhale slowly through the device to obtain the result, which will be recorded in the participant's source.

3) Cardiovascular Markers:
Blood samples will be used for measurement of a battery of cardiovascular biomarkers primarily focusing on three areas: glucose tolerance (fasting insulin, glucose, hemoglobin A1C), clotting markers (thrombin, fibrinogen, PAI-1), inflammatory markers (C-reactive protein, interleukin-6, D-Dimer). Secondary measures include: Fasting lipid profile (total cholesterol, triglycerides, HDL-C, LDL-C). Participants will be required to fast for a minimum of 8 hours. Ideally, participants will not eat or drink after midnight and blood draws will be done in the morning. After the blood draw, participants will be provided with a meal voucher so that they may eat before performing the remaining visit tasks. The following volumes and tubes will be collected: Two 5 mL SST tubes, one 10 mL EDTA tube and two 2.7 mL citrate tubes.
4) Additional Blood Samples:
Blood samples will also be used for assessing individual differences in nicotine metabolism by phenotyping (i.e., Nicotine Metabolic Ratio, NMR, which is phenotypically estimated as the ratio of 3-hydroxycotinine [3 HC] to cotinine [COT] in plasma). One 10 mL EDTA tube will be collected.

We will store blood for the purposes of analyzing additional cardiovascular biomarkers or genotyping of individual differences in nicotine metabolism (CYP2A6) analyses of nicotine metabolism (variation in CYP2A6) or nicotinic acetylcholine receptor gene subtypes. All samples will be stored at the University of Vermont Tracy Lab.

Biomarker shipping and storage:
Biomarkers will be shipped quarterly to the University of Vermont Laboratory for Clinical Biochemistry Research (Tracy Lab). The Tracy Lab will serve as a central repository for all biomarker specimens and will be responsible for distributing specimens to the appropriate labs on a quarterly basis. Urine samples will be analyzed and stored at the University of Minnesota Hecht Lab. Cardiovascular Biomarkers will be analyzed and stored at the Tracey Lab. Additional blood samples for the purposes of phenotyping will be analyzed and stored at the University of Toronto Tyndale Lab.

Baseline fMRI testing (University of Vermont only):
Participants at the UVM site will complete the neuroimaging battery two or three days after the first baseline assessment, depending on availability. This battery will be completed only among a randomly selected subset of participants in the lowest and the highest dose conditions (45 participants/dose condition for total of 90 participants), which will provide the greatest likelihood of detecting differences between nicotine doses. Forty-five participants from each of the two conditions will be selected with the goal of having 20 completers from each of the doses. Participants who consent to neuroimaging and meet the eligibility criteria will be encouraged to abstain from smoking for approximately 24 hours before their scan. Abstinence will be verified by expired breath carbon monoxide levels that have decreased by at least 50% from the measure taken during the Baseline 1 visit. The battery includes fMRI assessments that parallel the behavioral/cognitive assessments described above (i.e., a sustained attention task, inhibitory control test of executive function) and that are sensitive to abstinence-related disruptions in performance.

Prior to Baseline scan, participants will partake in a practice session of the fMRI cognitive battery tasks in a mock scanner at the Clinical Research Center (CRC) in order to practice each task in an environment that closely mimics that of the actual fMRI machine itself.

The neuroimaging battery also includes a high-resolution anatomical scan to assess total and regional grey matter volumes and cortical thickness, a resting- state scan to assess intra- and inter-regional brain connectivity, and arterial spin labeling to provide a quantitative measure of blood flow. Baseline characterization and comparison with a second scan approximately 12 weeks later will provide the potential for insights into the neurobiology of dependence and withdrawal (including individual differences in dependence severity) and differential changes that may arise from being exposed for an extended period to VLNC versus usual nicotine content levels in commercially available cigarettes.

Experimental Procedures

Experimental Period:

Participants will be seen weekly throughout the 12-week experimental period. Weeks 2, 6, 12 and the abstinence visit will take approximately 2-4 hours each. All other sessions will last approximately 2 hours. Upon arrival at the laboratory, participants will provide urine and breath BAL and CO samples. If the participant has a positive urine toxicology screen the Research Assistant will initiate the Field Sobriety SOP to determine if the participant can continue with the session or if it should be rescheduled. At the end of each experimental session, the researcher will complete the End of Visit Evaluation Form, which will be filed in the participant's binder. This will allow the researcher to make note of any problems encountered during the visit, to track which computers were used for which tasks, and to assess the truthfulness of the participant in regards to self-report of tobacco use and compliance to study procedures.

Visit scheduling requirements for experimental period:

The ideal scheduling window between each visit is 7 days based on the date of the Baseline 2 Visit. For additional scheduling requirements, refer to the '*Scheduling Visits SOP*'. If a participant misses a visit and is not able to reschedule during the window (± 3 days), that visit will not be 'made-up' in the future. All measures that were not completed will be considered missing data and will not be collected during future visits. If a visit mistakenly occurs outside of the designated window, this is a protocol deviation. A 'Non-Medical Event Form' will need to be completed. Additionally, each visit should occur at approximately the same time of day ± 2 hours.

If a participant is not able to attend his/her Week 12 visit, then it should be rescheduled even if it is outside of the scheduling window. This will be documented as a protocol deviation.

Experimental Visits Weeks 1, 3, 5, 7, 9, and 11 Procedures

Measures/Assessments

Physiological Measures Collected, recorded on paper, and entered into REDCap by the interviewer at the end of the visit:

1) BAL
2) Weight
3) CO
4) Blood Pressure
5) Heart Rate
6) Urine Toxicology

The following questionnaires will be participant-administered via paper at and then will be entered into REDCap by the interviewer at the end of the visit:

1) BDI
2) OASIS

The following assessments will be administered as an interview and will be entered into REDCap by the interviewer at the end of the visit:

1) Concomitant Medications
2) Medical Event Form, if applicable
3) Health Changes Questionnaire
4) Time Since Last Cigarette Questionnaire

The following assessments will be completed by the participant directly in REDCap:

1) MNWS
2) QSU brief - Usual Brand Cigarette
3) QSU brief - Study Cigarette
4) Cigarette Evaluation Scale - Study Cigarette

In the event that the REDCap website is not functioning, the assessments will be printed out and administered on paper. The source documents will be kept in the participant's binder. The interviewer will enter the data into REDCap when it resumes functioning properly. This information should be recorded in the 'End of Visit Evaluation Form' and filed in the participant's binder.

Experimental Visits Weeks 2, 4, 6, 8, 10 and 12 Procedures:

Measures/Assessments

Physiological measures collected, recorded on paper, and entered into REDCap by interviewer at the end of the visit:

1) BAL
2) Weight
3) CO
4) Blood Pressure
5) Heart Rate
6) Urine Toxicology
7) Urine Pregnancy test (if applicable

The following questionnaires will be participant-administered via paper at and then will be entered into REDCap by the interviewer at the end of the visit:

1) BDI
2) OASIS

The following assessments will be administered as an interview and will be entered into REDCap by the interviewer at the end of the visit:

1) Concomitant Medications
2) Medical Event Form, if applicable
3) Health Changes Questionnaire
4) Time Since Last Cigarette Questionnaire

The following assessments will be completed by the participant directly in REDCap:

1) Respiratory Health Questionnaire (weeks 2, 6 and 12 only)
2) FTND
3) Perceived Health Risks Questionnaire (weeks 2, 6 and 12 only)
4) Smoking Stages of Change Algorithm and Contemplation Ladder (Week 12 only)
5) Cigarette Purchase Task - Usual Brand Cigarette Version (weeks 2, 6 and 12 only)
6) Cigarette Purchase Task - Study Cigarette Version (weeks 2, 6 and 12 only)
7) WISDM-Brief
8) Drug Use Questionnaire - 1 month version (weeks 6 and 12 only)
9) PANAS (weeks 2, 4, 6, 8, 10 and 12)
10) Perceived Stress Scale (weeks 2, 6, and 12 only)
11) Alcohol Use Questionnaire - 1 month version (weeks 6 and 12 only)

In the event that the REDCap website is not functioning, the assessments will be printed out and administered on paper. The source documents will be kept in the participant's binder. The interviewer will enter the data into REDCap when it resumes functioning properly. This information should be recorded in the 'End of Visit Evaluation Form' and filed in the participant's binder.

Participants will also complete the following tasks:

1) Cognitive tasks (weeks 2, 6 and 12 only)
2) Smoking Topography - study cigarette (weeks 2, 6 and 12 only)

Week 12 fMRI testing (University of Vermont only):

Participants at the UVM site will also complete the neuroimaging battery again to assess changes after extended exposure to different doses. Participants who have initiated a quit attempt will not be asked to smoke prior to the scan. Participants willing to smoke the research cigarettes will take two puffs 30 minutes prior to the scan. If the participant is unwilling to smoke the research cigarette, they will be allowed to smoke their usual brand.

Biological Samples to be collected:

1) First void urine sample (Weeks 6 and 12 only)
2) Blood Samples (Weeks 6 and 12 only)
3) Collect FeNO (Weeks 6 and 12 only)

Interactive Voice Response System:

Participants will continue to use the IVR system on a daily basis throughout the experimental period to record the number of study cigarettes smoked per day and use of non-study

cigarettes. During the first week after Baseline 2, the IVR system will collect information about withdrawal symptoms.

Variable Incentive Program:
An incentive program has been developed with the goal of improving attendance at scheduled assessment sessions, compliance with using only study-provided tobacco products, and encouraging honest self-reports regarding all nicotine/tobacco use.

Briefly, participants will receive a total of five tickets for each weekly visit they attend after randomization (Visits 03-14, weeks 1-12). In total, participants could earn 60 valid tickets across the 12 visits. Participants will be instructed that these tickets correspond to attendance (one ticket), honest reporting (one ticket), and adherence to using only the assigned study product (three tickets). They will be further instructed that these tickets "could" be eligible for entry into a monthly drawing for prizes, but that only tickets that are "validated" will be eligible for prizes.

Since it is prohibitively expensive to test urine samples each week for each participant and because it is currently not feasible to detect with reasonable precision non-compliance based on biomarkers in the two higher nicotine group, we plan to only validate the attendance tickets. Hence, each participant who attends their regularly scheduled weekly session will have a total of five validated tickets entered into the monthly drawing.

To convey the message that we may be validating honest reporting and use of only study-provided products, we will collect a weekly urine specimen from participants. Further, in a bogus pipeline of sorts, participants will be instructed and that these urine specimens MAY be used to biochemically verify compliance to the study product by testing different nicotine and tobacco products found in the urine. Likewise, participants will also be instructed that their honesty ticket MAY be validated if their self-reported tobacco use matches what's in their urine. So there is some minor deception involved, but technically we could conduct urine toxicology testing for both purposes. Hence, if the urine toxicology testing is presented as something that MAY be done for validation purposes, we feel that any deception is relatively minor. For scientific/economic reasons we are just electing to restrict validation to attendance. Nevertheless, we will debrief all participants upon the completion of the trial. We will inform them that the incentive program was based exclusively on attendance due to the relatively high cost of urine toxicology testing and other practical problems with shipping the urines for prompt testing.

Drawings will be conducted on the 1st of each month. Validation will be performed by staff who have no participant interaction and are not blind to condition. Any ticket drawn will be eligible for an incentive as the only true contingency is for attendance. There will be no mention of the basis for earning incentives (i.e., whether the ticket was for attendance, honesty, adherence,). Participants will simply be informed that he or she earned an incentive from the drawing.

Each drawing will be independent (without replacement); consequently, some participants will not win a prize and others may win more than one during the study if more than one of their tickets is drawn. After confirming winners, the remaining tickets from each month will be discarded (i.e., tickets will only be entered into one drawing). The monthly prize amounts are detailed below.

We estimate based on the 2½ years we estimate it will take to complete this study, that

participants will win an average of approximately $65 in prizes or an additional $5.50 per week per participant.

Grand Prize (1): $500 cash
Second Prize (1): $200 cash
Third Prize (5): $10 cash

Product and Procedures Compliance Review Sessions:
At each visit, Baseline 2 through Week 11, participants will be counseled about their use of the study cigarettes. Participants will be asked about any concerns or obstacles associated with use of the study cigarettes. The importance of honest self-reporting will be stressed. Participants will be told that they will not be penalized for use of other nicotine or tobacco products and that it is crucial for them to report any use of these products. If difficulties are encountered, participants will be asked why they think they are experiencing difficulties (e.g., taste, withdrawal symptoms) and to problem-solve how to deal with these difficulties in order to meet the protocol requirements. Additionally, participants will be counseled about their IVR completion, visit attendance, task engagement and product accountability. Refer to the '*Product and Procedures Compliance Review Sessions SOP*' for more information.

Quit Attempts During the Study Protocol:
At each weekly session, we will ask the participant if he/she is currently abstaining from smoking with the intention of quitting. If the answer is no, then we will also ask if he/she is planning to quit smoking prior to his/her next scheduled visit.

If a Participant is Currently Abstaining from Smoking with the Intention to Quit:

- Encourage participant to continue abstaining from smoking
- Schedule the participant for normal weekly visits, but no puff topography
- Provide the participant with the '*Clearing the Air*' manual and local smoking cessation resources
- Give the participant the option to take home study product rather than require him/her to take the product
- If the participant choses to take home the study product have him/her sign a form acknowledging that cigarette availability could be detrimental to the quit attempt. Recommend that he/she put the product "away" at home as to avoid unwanted cues to smoke.
- If the participant chooses not to take home the study product, have him/her contact the lab if he/she lapses and would like to pick up or be mailed the study product prior to his/her next visit.

If a Participant is Planning to Quit Smoking, but has not initiated the quit attempt:

- Ask if he/she has identified a target quit date and, if so, what that target date is.
- Provide the participant with the '*Clearing the Air*' manual and local smoking cessation resources.
- Provide the participant with the study product as usual. Recommend that on the target date he/she put the product "away" at home as to avoid unwanted cues to smoke.

Abstinence Assessment Session:
After the week 12 visit, participants will be required to come back for one additional visit the following day. During this visit, participants will have been encouraged to abstain from smoking until their next scheduled visit (approximately 24 hours later). The abstinence assessment session should be scheduled no less than 18 hours and no more than 30 hours after the Week 12 visit. Abstinence will be verified by expired breath carbon monoxide levels that have decreased to $\leq$ 4 ppm. This session will allow us to determine whether the experimental cigarettes have reduced the effects of abstinence on these measures relative to the control conditions. If the participant does NOT meet abstinence criteria, he/she will only receive $20 for the visit.

Measures/Assessments

Physiological measures collected, recorded on paper, and entered into REDCap by the interviewer at the end of the visit:

1) BAL
2) CO
3) Blood Pressure
4) Heart Rate
5) Urine Toxicology

The following questionnaires will be participant-administered via paper at and then will be entered into REDCap by the interviewer at the end of the visit:

1) BDI
2) OASIS

The following assessments will be administered as an interview and will be entered into REDCap by the interviewer at the end of the visit:

1) Concomitant Medications
2) Medical Event Form, if applicable
3) Health Changes Questionnaire
4) Time Since Last Cigarette Questionnaire

The following assessments will be completed by the participant directly in REDCap:

1) MNWS
2) QSU-brief - Usual Cigarette
3) QSU-brief - Study Cigarette
4) Cigarette Purchase Task - Usual Brand Cigarette Version
5) Cigarette Purchase Task - Study Cigarette Version
6) Cigarette Evaluation Scale – Usual Brand Cigarette Version

In the event that the REDCap website is not functioning, the assessments will be printed out and administered on paper. The source documents will be kept in the participant's binder. The interviewer will enter the data into REDCap when it resumes functioning properly. This

information should be recorded in the ‘End of Visit Evaluation Form’ and filed in the subject’s binder.

Participants will also complete the following task:

1) Cognitive tasks
2) University of Vermont only: Participants at the UVM site will also complete the neuroimaging battery again to assess changes after extended exposure to different doses.

Participants who do NOT meet abstinence criteria will be required to complete the following assessments:

1) BAL
2) CO
3) Blood Pressure
4) Heart Rate
5) Urine Toxicology
6) Concomitant Medications
7) Health Changes Questionnaire
8) Medical Event Form, if applicable
9) TLFB

Participant Compensation:
Participants will receive $25 for completing the screening visit, plus an additional $25 bonus for completing the visit on time as scheduled. Payment will be made regardless of enrollment as long as the participant passes the drug test, breath alcohol test, and meets the minimum requirements for carbon monoxide or NicAlert levels. Participants who do not pass these tests will be dismissed from the screening visit without payment, except in the event they can produce a prescription for the medication that caused them to fail the drug test. Participants will receive $100 for each of the shorter sessions (Baseline 1, Weeks 1, 2, 3, 4, 5, 7, 8, 9, 10, 11), $150 for each of the longer sessions (Baseline 2, Weeks 6 &12), up to $160 for the abstinence visit ($150 for the visit + up to $10 for the preference test), $20 for biochemical verification of abstinence, up to $221 for completing daily IVR reports of study cigarette and other nicotine and tobacco use. Participants will also have a chance to earn an additional $50 bonus for every three visits that are completed on time as scheduled. There will also be a $100 bonus for completing the study for a total bonus of $325. If the participant does not attend the screening visit or one of the weekly visits as scheduled, they will forfeit the bonus. They will have a chance to earn another bonus payment with the next set of three visits. Participants who do not complete the entire study will receive compensation for the sessions that they do complete. UVM participants who undergo fMRI testing will receive an additional $150/scan. Total compensation for completing Study 2, including study visit payments, daily IVR calls and bonuses is $2301 (or $2601 if participating in the fMRI testing). Participants will also have a chance to earn additional money through the Variable Incentive program. As mentioned above, participants will have a chance to earn additional incentives each month for compliance, honesty and attendance, however, we anticipate that on average, participants will win approximately $150 in prizes.

End of Study:
After a participant has completed all study procedures and has been paid for participation the research assistant will read the following script and give the participant the *Clearing the Air Manual.*

"If you've reduced your smoking during this study, we encourage you to continue these reductions or even consider quitting. We would like to provide you with some resources should you decide to try to abstain from smoking (give "Clearing the Air" and hotline information). Please also feel free to consult with your physician and use any medications he/she deems appropriate. We will call you in approximately 30 days to ask about your smoking since leaving the study. There is no right answer and we know how difficult quitting can be. Please just answer honestly. The call will take less than 5 minutes. Thanks again for your participation."

The following assessments will be administered using REDCap:

1) End of Study Questionnaire

30 Day Follow up Phone Call:
Participants will receive a follow-up phone call between 25 and 35 days after the abstinence assessment session to assess their smoking patterns. The phone questionnaire will last less than five minutes. The questionnaire will ask if the participant is still smoking, how much and whether he/she has attempted to quit smoking since the end of the study. Participants will receive 5 variable incentive program lottery tickets for completing the call as compensation. Those who report abstinence will be invited to come in for biochemical verification and be compensated $40 for doing so. A urine sample will be collected to test urine cotinine levels. Additionally, any Medical Event Forms that remain open from the last session will be discussed. If the participant became pregnant during the study, this would have been recorded as a medical event. During this phone call, the research assistant will confirm her due date. This event will remain open until delivery. At that time the licensed medical professional will contact the participant to ask a few questions about the baby's health and will update the Medical Event Form.

Once a participant has completed all study procedures and all open events have been closed, the PI will review the participant's binder and sign a form indicating study completion for that participant.

Randomization
At the end of the Baseline 1 session, participants will be randomized into one of three cigarette conditions. Participants in each condition will be assigned cigarettes that match their menthol preference. Participants will be randomized, using block randomization, in equal number to the dose conditions, with randomization stratified by study site and menthol status. Each site will randomize participants until the total goal of 282 participants across both sites is reached, and no effort will be made to recruit a specific number of menthol and non-menthol smokers at each site.

Condition	TPMF Code	Type*	Specifications Nicotine Yield	Specifications Tar Yield	Specification Range for Nicotine Yield	Specifications Nicotine Content
1	NRC600	CN	0.8 ± 0.15	9 ± 1.5	0.65 - 0.95	15.30 ± 0.18
1	NRC601	CN-Men	0.8 ± 0.15	9 ± 1.5	0.65 - 0.95	16.03 ± 0.47
2	NRC300	RN	0.12 ± 0.03	9 ± 1.5	0.09 - 0.15	2.27 ± 0.08
2	NRC301	RN-Men	0.12 ± 0.03	9 ± 1.5	0.09 - 0.15	0.104 ± 0.002
3	NRC102	RN	0.03 ± 0.01	9 ± 1.5	0.02 - 0.04	0.37 ± 0.01
3	NRC103	RN-Men	0.03 ± 0.01	9 ± 1.5	0.02 - 0.04	0.39 ± 0.00

*Legend:	
RN	Reduced Nicotine
RN-Men	Reduced Nicotine-Menthol
CN	Conventional Nicotine
CN-Men	Conventional Nicotine-Menthol

The lead statistician will create a randomization schedule for each of the two sites, amounting to 150% of expected enrollment at each site. The excess randomization codes will be used in the event that a site will have to enroll extra participants due to unexpectedly slow enrollment at another site. The nicotine doses will be identified by letter code and the number 2 (V2, W2, X2, Y2) and only Administrative Core personnel with no participant contact will have the link between the statistician's letter code and dose assignments. The randomization schedules and the link between the alphabetic code and treatment assignment will be maintained securely by the Administrative Core. A second, sealed, copy will be secured in a separate building to protect against loss related to fire or other unforeseen events.

The University of Vermont will be responsible for removing all identifying information from cigarettes received from the Research Triangle Institute (RTI), labeling each carton with a blind code, assigning product using this blind code based on the randomization schedule being provided by the UVM Biostatistics Core, and shipping cigarettes to each site as needed based on recruitment. Each site will be responsible for tracking product received and distributed to participants, collecting unused product from participants, and returning unused cigarettes to

UVM. The participants, investigators and study staff will not have knowledge of which product is given to a participant or whether different participants received the same or different product.

During the experimental period, participants will be provided with a 14-day supply of research cigarettes equivalent to 150% of their daily smoking rate. This rate will be calculated at Baseline 2 and will be an average daily smoking rate based on the IVR data for the first seven days of the baseline period. This will ensure adequate availability of cigarettes in the numerous locations participants may typically keep a supply (home, work, vehicle, etc.) as well as avoid expending the entire supply if they miss a scheduled visit. Participants will be instructed to use the research cigarettes for 12 weeks, at which point they are to discontinue product use.

If there is prior knowledge a participant will be missing a visit (i.e. planned vacation, laboratory closure, etc.), then the participant will be provided with an adequate supply of cigarettes to make up for the missed visit(s). The participant will be given a 21-day supply if one visit is going to be missed and a 28-day supply if two visits are going to be missed.

Participants will be asked to refrain from use of other non-study cigarettes during the study period. If participants have to use another nicotine product, they will be told to use a non-combustible product (gum, patch, etc.). Additionally, they will be told there is not a penalty for use of non-study cigarettes, and that it is crucial for them to report any use of non-study cigarettes or other nicotine or tobacco products. Throughout the baseline and experimental periods, an Interactive Voice Response (IVR) system will be used on a daily basis to record the number of study cigarettes and non-study cigarettes used the previous day. During the baseline and first experimental week, participants will also answer daily IVR questions about their mood. Participants will be seen weekly for assessments. Brief standardized review sessions focusing on compliance with the study cigarettes and other study procedures will be provided at each visit. At the end of the 12-week trial, participants will undergo an assessment of withdrawal, craving, and cognitive function following a brief period of abstinence.

Product Accountability:

Participants will be required to keep track of all the cigarettes provided to them. Therefore, they will be instructed to return all unused cigarettes and empty cigarette packs to the laboratory each week. Research staff will complete the 'Product Accountability Log' with the participants. Any discrepancies in the product dispensed versus product returned will be discussed and recorded in the log. Empty cigarette packs will not be saved. Unused cigarette packs will be re-distributed to the participants during Weeks 1-11. During Week 12, any remaining unused cigarettes returned by the participants will be collected by the research staff.

Participants who report running out of cigarettes prior to a scheduled weekly visit will be allowed to come in for an unscheduled visit to obtain more research cigarettes. If a participant has more than two unanticipated visits we will determine if a rate change is necessary. To determine this, we will look at the past two CO levels as compared to the Baseline 2 CO. If the CO trend is consistent with the self-report of smoking all of the allotted cigarettes then a rate increase will be granted. The participant will then receive cigarettes at a rate of 175% of their daily smoking rate. The maximum increase is 200% of their daily smoking rate. If participants lose more than two packs of cigarettes and require an unscheduled visit to the laboratory to supplement their

supply, they will be told the next time they lose more than two packs they will have to wait until their next scheduled appointment to receive more cigarettes.

Statistical Methods and Sample Size

Statistical methods. See Statistical Analysis Plan at the end of this Supplemental document.

Sample size. Sample size for other analyses was determined using power analysis for hypothesis tests related to the Primary Aim of Study 2, specifically to detect a significant difference between the reduced-nicotine conditions and the high-nicotine yield condition in the primary endpoints, cigarettes per day (CPD) and urine cotinine, at the end of the trial. Donny et al. (2015) found a reduction of 4.52 CPD and 6.07 CPD among subjects smoking 2.4 mg/g and 0.04 mg/g cigarettes, respectively, compared to those smoking normal nicotine cigarettes. In addition, they reported a decrease of 0.59 and 0.39 in urine cotinine among those smoking these same RNC cigarettes, compared to those smoking NNC cigarettes. A sample size of 69 completers per condition will provide 90% power to detect similar differences in CPD and greater than 95% power to detect differences in urine cotinine, with a two-sided type I error rates of 0.02. The type I error rate reflects the Bonferroni correction needed to allow testing of all pair-wise comparisons. Regarding fMRI power, the analysis was based on the estimated effect size of 2.04 (Cohen's d) from the cortical activation differences previously observed between smokers and ex-smokers on the same inhibitory control task proposed here (Nestor et al., 2011). With 20 completers in each condition, there is 80% power at $p = 0.05$ to detect effects about half as large (Cohen $d=0.91$) between any two conditions.

Potential Risks of Participation

1) Survey Questionnaires: The interview will include questions about medical history, drug and alcohol use, and questionnaires about mood. Answering these personal questions could make the participant feel uncomfortable.
2) Breach of Confidentiality: The risk of the interview is loss of privacy if other people find out the results.
3) Coercion: Coercion is a possible risk due to monetary compensation for participating in these studies. The likelihood of this risk is low because the compensation is commensurate with the amount of time and effort required for these studies.
4) Drug Testing: A breach of confidentiality could occur and other people could learn of the participant's drug use.
5) Obtaining blood pressure: The blood pressure cuff may cause minimal discomfort. In obtaining blood pressure, researchers may find out the participant has abnormal blood pressure.
6) Smoking Cigarettes: All cigarettes are detrimental to a person's health and can lead to significant medical problems including:
    g. Cardiovascular Diseases: Coronary heart disease, heart attack, stroke, peripheral vascular disease, reduced blood circulation, abdominal aortic aneurysm
    h. Respiratory Diseases: Emphysema, bronchitis, and chronic airway obstruction
    i. Cancers: Cancer of the lung, bladder, cervix, esophagus, kidney, larynx, mouth, pancreas, throat, and stomach; leukemia
    j. Metabolic Diseases: Type 2 Diabetes

k. Other Health Risks Associated with Smoking: Including but not limited to infertility, lower bone density in postmenopausal women, and hip fracture in women
l. Death

7) Smoking study cigarettes: In addition to the above medical problems, participants may experience some minor adverse health effects such as headaches or experience withdrawal symptoms, which are listed below. Due to the altered nicotine levels, there could be a change in their cigarette use including the manner in which they inhale the smoke. Smoking the study cigarettes does not provide any less risk than their usual brand cigarette and could pose increased health risks. Participants may also experience increases in levels of carbon monoxide, a gas from smoke.

8) Smoking Withdrawal: Participants may experience smoking withdrawal symptoms during this study. The symptoms can be uncomfortable but are typically of minimal risk. Smoking withdrawal symptoms include:
    a. Anger, irritability, frustration
    b. Anxiousness, nervousness
    c. Depressed mood or sadness
    d. Desire or craving to smoke
    e. Difficulty concentrating
    f. Increased appetite, hunger or weight gain
    g. Insomnia, problems sleeping or awakening at night
    h. Restlessness
    i. Impatience
    j. Constipation
    k. Dizziness
    l. Coughing
    m. Dreaming or nightmares
    n. Nausea
    o. Sore Throat

9) Returning to Regular Smoking: It is possible that if participants return to smoking their usual brand of cigarette at the end of the study they may experience mild and transient nausea, dizziness, and lightheadedness.

10) Risk to Fetus**:** Smoking during pregnancy can lead to miscarriage, preterm delivery, stillbirth, low birth weight, problems with the placenta, birth defects such as cleft palate, sudden infant death syndrome (SIDS), and early childhood behavioral problems.

11) Changes in blood pressure and/or heart rate: Smoking and nicotine can affect the cardiovascular system, which may result in changes in blood pressure and/or heart rate.

12) Exacerbation of psychiatric symptoms: Smoking and nicotine can affect a person's mood and emotions and are associated with psychiatric disorders including major depressive disorder, general anxiety disorder, bipolar disorder and eating disorders. Any changes in nicotine or cigarettes consumption could adversely affect psychiatric conditions.

13) MRI: The MRI scanner produces a loud banging noise and may be uncomfortable for people who become anxious in confined spaces. The presence of metal in or on a participant's body during an MRI scan can present a serious health risk. The MRI staff will ask participants in detail about any possible metal they may have in or on them. Regarding unexpected MRI findings, the participant will be informed of what was found. In addition, information about the incidental finding can be provided to the participant's primary doctor or the study team can refer them to an appropriate specialist. The costs

for any care that would be needed to diagnose or treat an incidental finding would not be covered by the research study and would be the responsibility of the participant.

Avoiding Risks to Fetus:

If participants choose to be sexually active, they should use an appropriate "double barrier" method of birth control (such as female use of a diaphragm, or contraceptive sponge, in addition to male use of a condom) or the female should be using prescribed "birth control" pills, patch, ring, injections, or implants or intrauterine device (IUD). Participants will be tested for pregnancy every two weeks beginning at screening through the last study visit. If a participant becomes pregnant during the study, she will be withdrawn from the study. Approximately 30 days after being withdrawn or having a positive pregnancy test at the last study visit, the research staff will call the participant to confirm her due date. The licensed medical professional will follow-up with the participant after delivery to ask questions about the baby's health.

Expected benefits of participation:

There are no immediate benefits from participating in the study. The information obtained from this study may ultimately help the Food and Drug Administration decide how best to regulate tobacco products with the goal of improving public health.

Study Debriefing:

After data collection is complete, participants will receive a letter telling them which condition they were randomized into and the results of the study thus far.

Protection Against Risk

Research data without identifiers will be maintained in a locked file cabinet and on password-protected computers in the research staff workplace, with only code numbers identifying subjects. Study consent forms and the linkage between the participants' names and codes will be stored in a locked file cabinet. Interviews with participants will be conducted in private rooms. Urine samples for drug and pregnancy tests and tobacco exposure biomarkers will be obtained in a private bathroom within the laboratory suite. Blood draws will be performed in a private patient room. Subjective measures will be administered electronically. The biostatistics and data-management team will provide consistent data-management practices for all data in the Center. Validity and reliability of data will be maximized by using REDCap, which is housed on the Fletcher Allen Health Care, HIPAA compliant, computing system. REDCap is a secure, web-based system that accommodates local and remote data collection by each project team, and allows for data entry work-flow monitoring and data quality control monitoring by biometry staff. For data integrity, data entry windows will follow the structure of paper forms as much as possible to allow for ease of entry, and will use predefined choices to minimize errors when possible. Data quality monitoring will be facilitated with periodic down loads and analysis using a variety of common statistical program format such as SAS, Stata, R, and SPSS. Quality control procedures will be conducted for all data collected, including analysis of missing data and logic checks for out of range and other anomalous values. This secure electronic data gathering and transmission plan, overseen by the experienced biostatistical team, will minimize opportunities for breaches of confidentiality. Biological samples for nicotine and carcinogen biomarker analysis will be marked with participant ID, stored in the locked laboratory suite, and sent to a laboratory for analysis on a quarterly basis.

All information collected as part of this study will be accessible only to research staff. No information will be shared with participants' clinicians unless the participant requests this in writing. All investigators and staff have undergone (and any new staff will undergo) human subjects' ethics training as required by UVM and are fully conversant with relevant ethical principals around confidentiality. Assessments, consenting and study procedures will be closely supervised by the PI.

The sponsors (NIDA/FDA) as well as the Institutional Review Board and regulatory authorities could be granted direct access to original medical and research records for verification of clinical trial procedures and/or data. If this is required, it will be done under conditions that will protect privacy to the fullest extent possible consistent with laws relating to public disclosure of information and the law-enforcement responsibilities of the agency.

Data Storage:
Data will be stored locally at each site, at the University of Minnesota Masonic Cancer Center's Bioinformatics Core and at the University of Vermont. Long-term storage of all study data, for at least 7 years after study completion, will be at the University of Vermont.

Adverse Events
The research assistant will ask about adverse events at each session, using a form that assesses the nature, severity, duration, action taken, and outcome of study-related adverse events. AEs will be captured from the time of first study cigarette. Participants will be given contact cards to inform us of events that occur between study contacts. Any AE that remains open will be reviewed and closed at an interview conducted 30 days after the study completion date (completers) or when the study should have ended had the participant completed the study (dropouts and those withdrawn by investigator).

All procedures will be monitored to ensure that they conform to the approved protocol. In addition, monitoring will be done of all unforeseen circumstances that might arise and affect safety; of all reports of serious adverse events as defined in 38 CFR 46 (death, new or prolonged hospitalization, persistent or significant disability/incapacity); of other significant adverse events (adverse events that lead to drop out by the participant or termination by the investigator); of unexpected adverse events resulting from the study, and of expected adverse events.

Any SAE will be brought to the attention of the site PIs as soon as possible and not longer than 24 hours. Any AE or SAE that is both unexpected and related to study participation will be reported to the IRB within 7 days of the event. The local IRB will make a determination as to whether additional reporting requirements are needed. IRB actions will be reported to the funding agency by the PIs no less than annually and more frequently as recommended by the local IRB. Any SAEs will be summarized in the yearly Progress Reports to the funding agency, including a review of frequency and severity. All SAEs will be followed through ongoing consultation with the physician caring for the patient until they resolve, result in death, or stabilize and are not expected to improve. The study staff will be in close contact with participants and health care providers throughout the study to monitor for potential unanticipated

problems. Any unanticipated problems will be discussed at the weekly research staff meetings and reported as required to the local IRB.

Withdrawal or Monitoring of Participants

For the participant's protection, participants will be withdrawn immediately from the study if any of the following occur:

1) Cardiovascular disease (CVD) event: Typically includes MI (heart attack), PTCA (angioplasty/stenting), bypass surgery, stroke, peripheral vascular disease (arterial blockages in arms or legs leading to procedure or surgery). Less common CVD problems would be new cardiac arrhythmias (e.g., new atrial fibrillation) or new valvular disease (e.g., mitral or aortic regurgitation).
2) DVT/PE (deep vein thrombosis/pulmonary embolism, i.e., blood clots in the venous system).
3) Suicide Attempt: A participant will be withdrawn if he/she attempts suicide at any time during participation in the study.
4) Psychiatric Hospitalization: A participant will be withdrawn if he/she is hospitalized for psychiatric reasons at any time during participation in the study.
5) Pregnancy: If participant indicates she is pregnant or has a positive pregnancy test at any time during the study, she will be withdrawn from the study, and this event will remain open until delivery. At that time the licensed medical professional will contact the participant to ask a few questions about the baby's health and will update the open 'Medical Event Form'. A positive pregnancy test at Session 14 in Study 1 or Week 12 in Study 2 will trigger a 'Medical Event Form' to be completed but will not result in withdrawal since she is no longer receiving study product.
6) Expired breath carbon monoxide increase: A participant will be withdrawn from the study if the average of two consecutive CO readings during the same visit is 100 ppm or greater.
7) Marked increase in smoking: A participant will be withdrawn from the study if he/she meets **BOTH** of the following criteria for two consecutive weeks
    a. Cigarette per day increase: The average CPD increases by more than 100% from the average CPD during baseline.
    b. Expired breath carbon monoxide increase: If the average of two consecutive CO measurements in the same visit is
        i. CO is greater than 50 ppm if CO at Baseline 1 is <20 ppm.
        ii. CO is greater than 60 ppm if CO at Baseline 1 is 20 – 34 ppm.
        iii. CO is greater than 70 ppm if CO at Baseline 1 is 35 – 49 ppm.
        iv. CO is greater than 80 ppm if CO at Baseline 1 is 50 – 64 ppm.
        v. CO is greater than 90 ppm if CO at Baseline 1 is 65 – 80 ppm.
8) Note: If the second consecutive visit is the last study visit, then the participant would not be withdrawn from the study.

The following will be monitored and can lead to the participant being withdrawn by the PI or Licensed Medical Professional:

1) Cigarettes per day increase: Continued participation will be evaluated by the site PI if the average number of cigarettes per day (CPD) increases by more than 100% from the average CPD during baseline as determined by CPD at Baseline 2.

2) Blood pressure (BP) or heart rate (HR) changes: If any of the following occur post-enrollment: 1) BP is at or above 160/100 or below 90/50, or 2) HR is at or above 115 bpm or below 45 bpm a manual blood pressure and heart rate measurement will be taken after 10 minutes have passed. If the manual reading is still out of range, a 'Blood Pressure and Heart Rate Symptom Checklist' and 'Medical Event Form' will be completed, and the participant will be monitored by the medical professional.
3) Expired breath Carbon Monoxide increase: If the average of two consecutive CO measurements meets the criteria below then the 'Medical Event Form' will be completed and the participant will be monitored by the licensed medical professional.
    a. CO is greater than 50 ppm if CO at Baseline 1 is <20 ppm.
    b. CO is greater than 60 ppm if CO at Baseline 1 is 20 – 34 ppm.
    c. CO is greater than 70 ppm if CO at Baseline 1 is 35 – 49 ppm.
    d. CO is greater than 80 ppm if CO at Baseline 1 is 50 – 64 ppm.
    e. CO is greater than 90 ppm if CO at Baseline 1 is 65 – 80 ppm.
4) Any hospitalization or debilitation in which participation in the study could be detrimental to the recovery process. This will be self-reported by the participant and will be reviewed by the site PI and licensed medical professional to determine whether continued participation in the study is appropriate.
5) If a participant is behaving in an inappropriate or threatening manner, admits to lying about eligibility criteria, is participating in other smoking research studies that could affect the primary outcome measures, etc., then the PI can withdraw him/her from the study at the PI's discretion.
6) If a participant fails to attend regularly scheduled research assessment visits or comply with the research procedures or schedule, then the PI can withdraw him/her from the study at the PI's discretion.
7) Increase in psychiatric symptoms: Exacerbation in symptoms noted during the study (i.e., change in BDI category from mild to moderate or moderate to severe) will trigger review by the study's licensed medical professional. The PI will withdraw the participant upon the licensed medical professional's recommendation.

Data Safety Monitoring Board

A Data and Safety Monitoring Board (DSMB) has been established to monitor safety outcomes and will be comprised of five members. The DSMB will be chaired by Dr. Eden Evins, Associate Professor of Psychiatry at Harvard Medical School and Director of the Center for Addiction Medicine at Massachusetts General Hospital. Other members include: Kevin Delucchi, PhD., Professor in Residence of Biostatistics in Psychiatry at the University of California San Francisco and Director of the Quantitative Core of the San Francisco Treatment Research Center; Hendree E. Jones, Ph.D., Professor of Obstetrics and Gynecology and Director of UNC Horizons at University of North Carolina Chapel Hill; Wallace Pickworth, Ph.D., Research Leader, Baltimore Operation, Centers for Public Health Research and Evaluation, Battelle; Kimber Richter, Ph.D., M.P.H., Associate Professor of Preventive Medicine and Public Health at the University of Kansas and Director of the University of Kansas Hospital's tobacco treatment program.

Conflict of interest

None of the members will be otherwise affiliated with the center and each member will complete a conflict of interest disclosure form prior to each meeting. Ad hoc specialists may be invited to participate as non-voting members at any time if additional expertise is desired.

Monitoring activities and frequency of meetings

The DSMB will set their own agenda and decisions about monitoring; e.g. how frequently to monitor, what threshold requires changes to protocol or stopping the study, and whether to view raw or analyzed data. The DSMB will be given FDA and EMEA guidelines for DSMBs and recent reviews on DSMBs. A brief report will be generated from each meeting for the study record and forwarded to each of the study site's Institutional Review Boards (IRB) and NIDA's Program Officer with the progress report. The DSMB will be available to convene outside of the regular meetings, if necessary. If concerns should arise regarding a particular subject, or any troublesome trends in the experiences of participants, they will make appropriate recommendations for changes in protocol, as needed. The project investigators will continue to examine safety data, blind to study condition, in case they wish to make study modifications. Before modifications are made, they will inform the DSMB and request their comments.

Communication plan to IRB, NIDA, and FDA (if applicable)

All IRBs, the FDA and the NIDA's Program Officer will be informed of any significant action taken as a result of the Data and Monitoring Board's findings. Study Participants will be informed of any changes in risk.

Protection of confidentiality

For DSMB meetings only de-identified data, including blinded study site and condition type, will be provided to the board. All data and discussion during the meeting will be confidential.

Investigational Tobacco Product

The Vermont Center on Tobacco and Regulatory Science has received an Investigational Tobacco Product (ITP) application from the FDA to cover the experimental cigarettes being used in this study. This application encompasses both trial sites.

Certificate of Confidentiality

To help protect the participant's privacy, Dr. Stephen Higgins, PhD, has received a Certificate of Confidentiality from the National Institutes of Health. With this certificate, the researchers cannot be forced to disclose information that may identify the participants, even by a court subpoena, in any federal, state, or local civil, criminal, administrative, legislative, or other proceedings. The researchers will use the Certificate to resist any demands for information that would identify the participants, except as explained below. The Certificate cannot be used to resist a demand for information from personnel of the United States Government that is used for auditing or evaluation of federally funded projects or for information that must be disclosed in order to meet the requirements of the Federal Food and Drug Administration (FDA).

The Certificate of Confidentiality does not prevent the participant or a member of their family from voluntarily releasing information about themself and their involvement in the research. If an insurer, employer or other person obtains the participant's written consent to receive research information, then the researcher may not use the Certificate to withhold that information.

The Certificate of Confidentiality does not prevent the researchers from disclosing voluntarily, without consent, information that would identify the individual as a participant of the research

project in instances such as evidence of child abuse or a participant's threatened violence to self or others.

Outcome Variables

Primary Endpoints:

2) Total number of cigarettes smoked per day (CPD) during Week 12 is the primary outcome;

Secondary Endpoints:

16) Study CPD during Week 12, total and study CPD across weeks, simulated consumer demand
17) Measures of adherence: non-study cigarette use, drop-out rate
18) Measures of psychiatric symptoms: BDI, OASIS
19) Measures of discomfort/dysfunction: MNWS, QSU
20) Measures of other health-related behaviors: breath alcohol, urine drug screen, TLFB-drug use, Alcohol Use Questionnaire, Drug Use Questionnaire, weight
21) Measures of nicotine/tobacco dependence: FTND, WISDM
22) Measures of tobacco exposure: CO, total nicotine equivalents, NNAL, minor alkaloids
23) Measures of intention to quit: Stages of Change, Contemplation Ladder
24) Measures of compensatory smoking: puff topography, filter analysis
25) Measures of other tobacco use: TLFB-other tobacco
26) Measures of cigarette characteristics: CES
27) Measures of cognitive function: BRIEF-A, EQ-5D, TPQ, D-KEFS, WASI-II, DDT, SST
28) Measures of cardiovascular function: heart rate, blood pressure, urine 11-dehydroTXB2
29) Measures of perceived risk: Perceived Health Risk Questionnaire
30) Safety outcome variables: Adverse Events (AEs), Serious Adverse Events (SAEs)

References

Andrews, C.M., Krantz, M.J., Wedam, E.F., Marcuson, M.J., Capacchione, J.F., & Haigney, M.C. (2009). Methadone-induced mortality in the treatment of chronic pain: role of QT prolongation. *Cardiology Journal, 16(3),* 210-217.

Beck, A. T., Ward, C., & Mendelson, M. (1961). Beck depression inventory (BDI). *Archives of General Psychiatry, 4,* 561-571.

Benowitz, N. L., & Henningfield, J. E. (1994). Establishing a nicotine threshold for addiction. The implications for tobacco regulation. *New England Journal of Medicine, 331,* 123-125.

Blank, M. D., Disharoon, S., & Eissenberg, T. (2009). Comparison of methods for measurement of smoking behavior: Mouthpiece-based computerized devices versus direct observation. *Nicotine & Tobacco Research, 11,* 896-903.

Brauer, L.H., Hatsukami, D., Hanson, K., & Shiffman, S. (1996). Smoking topography in tobacco chippers and dependent smokers. *Addictive Behaviors, 21*(*2*), 233-238.

Clemmey, P., Brooner, R., Chutuape, M.A., Kidorf, M., & Stitzer, M. (1997). Smoking habits and attitudes in a methadone maintenance treatment population. *Drug and Alcohol Dependence, 44(2-3),* 123-132.

Cohen, J. (1988). *Statistical power analysis for the behavioral sciences* (2nd ed.). New Jersey: Lawrence Erlbaum Associates, Publishers.

Cohen, S., Kamarck, T., & Mermelstein, R. (1983). A global measure of perceived stress. *Journal of Health and Social Behavior, 24(4),* 385-396.

Cox, L. S., Tiffany, S. T., & Christien, A. G. (2001). Evaluation of the brief questionnaire of smoking urges (QSU-brief) in laboratory and clinical settings. *Nicotine & Tobacco Research, 3,* 7-17.

DiClemente, C.C., Prochaska, J.O., Fairhurst, S.K., Velicer, W.F., Velasquez, M.M., & Rossi, J.S. (1991). The process of smoking cessation: an analysis of precontemplation, contemplation, and preparation stages of change*. Journal of Consulting and Clinical Psychology, 59(2),* 295-304.

Donny, E.C., Denlinger, R.L, Tidey, J. W., Koopmeiners, J. S., Benowitz, N. L.,Vandrey, R. G., … Hatsukami, D. K. (2015). Randomized trial of reduced-nicotine standards for cigarettes. *New England Journal of Medicine* 373(*Suppl* 14): 1340-1349.

Engström, A., Adamsson, C., Allebeck, P., & Rydberg, U. (1991). Mortality in patients with substance abuse: a follow-up in Stockholm County, 1973-1984*. The International Journal of the Addictions, 26(1),* 91-106.

Ernst, M., Heishman, S. J., Spurgeon, L., & London, E. D. (2001). Smoking history and nicotine effects on cognitive performance. *Neuropsychopharmacology, 25,* 313-319.

George, S., Moreira, K., & Fapohunda, M. (2008). Methadone and the heart: what the clinician needs to know. *Current Drug Abuse Reviews, 1(3),* 297-302.

Grinshpoon, A., Barchana, M., Lipshitz, I., Rosca, P., Weizman, A., & Ponizovsky, A.M. (2011). Methadone maintenance and cancer risk: an Israeli case registry study. *Drug and Alcohol Dependence, 119(1-2),* 88-92.

Hatsukami, D., Kotlyar, M., Hertsgaard, L. A., Zhang, Y., Carmella, S. G., Jensen, J. A., ... Hecht, S. S. (2010). Reduced nicotine content cigarettes: Effects on toxicant exposure, dependence and cessation. *Addiction, 105,* 343-55.

Heatherton, T. F., Kozlowski, L. T., Frecker, R. C., & Fagerström, K. O. (1991). The Fagerström Test for Nicotine Dependence: A revision of the Fagerström Tolerance Questionnaire. *British Journal of Addictions, 86,* 1119-1127.

Heishman, S. J. (1999, September). Behavioral and cognitive effects of smoking: Relationship to nicotine addiction. *Nicotine & Tobacco Research, 1(Suppl 2),* S143–S147.

Herning, R.I, Jones, R.T., Bachman, J., & Mines, A.H. (1981). Puff volume increases when low-nicotine cigarettes are smoked. *British Medical Journal (Clinical Research Ed), 283,* 187-189.

Hser, Y.I., McCarthy, W.J., & Anglin, M.D. (1994). Tobacco use as a distal predictor of mortality among long-term narcotics addicts. *Preventive Medicine, 23(1),* 61-69.

Hughes, J. R., & Hatsukami, D. K. (1986). Signs and symptoms of tobacco withdrawal. *Archives of General Psychiatry, 43,* 289-294. Hughes, J. R., & Hatsukami, D. K. (1998). Errors in using tobacco withdrawal scales. *Tobacco Control, 7,* 92-93.

Huh, B., & Park, C.H. (2010). Retrospective analysis of low-dose methadone and QTc prolongation in chronic pain patients. *Korean Journal of Anesthesiology, 58(4),* 338-343.

Hurt, R.D., Offord, K.P., Croghan, I.T., Gomez-Dahl, L., Kottke, T.E., Morse, R.M., & Melton, L.J. 3rd. (1996). Mortality following inpatient addictions treatment. Role of tobacco use in a community-based cohort. *Journal of American Medical Association, 275(14),* 1097-1103.

Justo, D., Gal-Oz, A., Paran, Y., Goldin, Y., & Zeltser, D. (2006). Methadone-associated Torsades de Pointes (polymorphic ventricular tachycardia) in opioid-dependent patients. *Addiction, 101(9),* 1333-1338.

Kleykamp, B. A., Jennings, J. M., Blank, M. D., & Eissenberg, T. (2005). The Effects of Nicotine on Attention and Working Memory in Never-Smokers. *Psychology of Addictive Behaviors, 19(4),* 433–438.

Logan, G. D., Cowan, W. B., & Davis, K. A. (1984). On the ability to inhibit simple and choice reaction time responses: A model and a method. *Journal of Experimental Psychology: Human Perception and Performance, 10(2),* 276–291.

MacKillop, J., Murphy, J. G., Ray, L. A., Eisenberg, D. T., Lisman, S. A., Lum, J. K., & Wilson, D. S. (2008). Further validation of a cigarette purchase task for assessing the relative reinforcing efficacy of nicotine in college smokers. *Experimental and Clinical Psychopharmacology, 16,* 57-65.

McLellan, A.T., Luborsky, L, Cacciola, J., Griffith, J., Evans, F., Barr, H.L., & O'Brien, C.P. (1985). New data from the Addiction Severity Index. Reliability and validity in three centers. *The Journal of Nervous and Mental Disease, 173(7),* 412-423.

Modesto-Lowe, V., Brooks, D., & Petry, N. (2010). Methadone deaths: risk factors in pain and addicted populations. *Journal of General Internal Medicine, 25(4),* 305-309.

Myers, C. S., Taylor, R. C., Moolchan, E. T., & Heishman, S. J. (2008). Dose-related enhancement of mood and cognition in smokers administered nicotine nasal spray. *Neuropsychopharmacology, 33(3),* 588-598.

Mysels, D. J. & Sullivan, M. A. (2010). The relationship between opioid and sugar intake: Review of evidence and clinical applications. *Journal of Opioid Management, 6(6)*, 445–452.

Nahvi, S., Richter, K., Li, X., Modali, L., and Arnsten, J. (2006). Cigarette smoking and interest in quitting in methadone maintenance patients. *Addictive Behaviors, 31(11)*, 2127–2134.

Nestor, L., McCabe, E., Jones, J., Clancy, L., & Garavan, H. (2011). Differences in "bottom-up" and "top-down" neural activity in current and former cigarette smokers: Evidence for neural substrates which may promote nicotine abstinence through increased cognitive control. *Neuroimage, 56,* 2258-2275.

Norman, S. B., Hami Cissell, S., Means-Christensen, A. J., & Stein, M. B. (2006). Development and validation of an overall anxiety severity and impairment scale (OASIS). *Depression and Anxiety, 23(4),* 245-249.

Piper, M. E., McCarthy, D. E., Bolt, D. M., Smith, S. S., Lerman, C., Benowitz, N., ... Baker, T. B. (2008). Assessing dimensions of nicotine dependence: An evaluation of the Nicotine Dependence Syndrome Scale (NDSS) and the Wisconsin Inventory of Smoking Dependence Motives (WISDM). *Nicotine & Tobacco Research, 10,* 1009-1020.

Reid, M., Fallon, B., Sonne, S., Flammino, F., Nunes, E., Jiang, H., … Rotrosen, J. (2008). Smoking cessation treatment in community-based substance abuse rehabilitation programs. *Journal of Substance Abuse Treatment, 35(1),* 68–77.

Richter, K. P., Gibson, C. A., Ahluwalia, J. S., and Schmelzle, K. H. (2001). Tobacco use and quit attempts among methadone maintenance clients. *American Journal of Public Health, 91(2),* 296.

Richter, K. P., McCool, R. M., Cately, D., Hall, M., and Ahluwalia, J. S. (2006). Dual pharmacotherapy and motivational interviewing for tobacco dependence among drug treatment patients. *Journal of Addictive Diseases, 24(4),* 79–90.

Robinson, J. C., & Forbes, W.F. (1975) The Role of Carbon Monoxide in Cigarette Smoking. *Archives of Environmental Health. 30(9),* 425-434

Roy, A. K., McCarthy, C., Kiernan, G., McGorrian, C., Keenan, E., Mahon, N. G., and Sweeney, B. (2012). Increased incidence of qt interval prolongation in a population receiving lower doses of methadone maintenance therapy. *Addiction, 107(6),* 1132–1139.

Rycroft, N., Hutton, S. B., & Rusted, J. M. (2006). The antisaccade task as an index of sustained goal activation in working memory: modulation by nicotine. *Psychopharmacology, 188(4),* 521-529

Substance Abuse and Mental Health Services Administration (SAMHSA). (2008). *Results from the 2007 National Survey on Drug Use and Health: National findings* (Office of Applied Studies, NSDUH Series H-34, DHHS Publication No. SMA 08-4343). Rockville, MD.

Sheehan, D. V., Lecrubier, Y., Sheehan, K. H., Amorim, P., Janavs, J., Weiller, E., ... Dunbar, G. C. (1997). The validity of the Mini International Neuropsychiatric Interview (MINI) according to the SCID-P and its reliability. *European Psychiatry, 12,* 232-241.

Sirota, A. D., Rohsenow, D. J., Dolan, S. L., Martin, R. A., & Kahler, C. W. (2013). Intolerance for discomfort among smokers: Comparison of smoking-specific and non-specific measures to smoking history and patterns. *Addictive Behaviors, 38(3),* 1782-1787.

Strasser, A. A., Lerman, C., Sanborn, P. M., Pickworth, W. B., & Feldman, E. A. (2007). New lower nicotine cigarettes can produce compensatory smoking and increased carbon monoxide exposure. *Drug and Alcohol Dependence, 86,* 294-300.

Stringer, J., Welsh, C., and Tommasello, A. (2009). Methadone-associated q-t interval prolongation and torsades de pointes. *American Journal of Health-System Pharmacy, 66(9),* 825–833.

Stroop, J. R. (1935). Studies of interference in serial verbal reactions. *Journal of Experimental Psychology, 18(6)*:643–662.

Tengs, T.O., Ahmad, S., Savage, J.M., Moore, R., Gage, E. (2005). The AMA proposal to mandate nicotine reduction in cigarettes: a simulation of the population health impacts. *Preventive Medicine, 40,* 170-80.

Tiffany, S. T., & Drobes, D. J. (1991). The *development* and initial validation of a questionnaire on smoking urges. *British Journal of Addiction, 86,* 1467-1476.

Wallner, C., Stollberger, C., Hlavin, A., Finsterer, J., Hager, I., and Hermann, P. (2008). Electrocardio-graphic abnormalities in opiate addicts*. Addiction, 103(12),* 1987–1993.

Watson, D., Clark, L. A., and Tellegen, A. (1988). Development and validation of brief measures of positive and negative affect: the PANAS scales. *Journal of Personality and Social Psychology, 54(6),* 1063.

Westman, E., Levin, E., & Rose, J. (1992). Smoking while wearing the nicotine patch: Is smoking satisfying or harmful? *Clinical Research, 40,* 871A.

STUDY PROTOCOL: SMOKERS WITH SOCIOECONOMIC DISADVANTAGE (WOMEN OF REPRODUCTIVE AGE

Table of Contents

Objective: 89
Background Information 89
Special Health Risks of Smoking Among Women: 89
Cigarettes to Be Assessed in This Study 90
Screening Procedures 90
______Recruitment 90
______Informed Consent Process 91
______Screening Measures/Assesments and Physiological Samples to be Collected 91
______Suicidality/Mental Health Monitoring 93
______Includion/Exclusion Criters 93
______Eligibility Determination 95
Baseline Procedures 96
______Measures/Assesments and Physiological Samples to be Collected 96
______Cognitive Tasks 98
______Smoking Topography 99
______Interactive Voice Response 13
______Description of Biological Specimens 99
______Biomarker Shipping and Storage 100
______fMRI Testing 100
Experimental Procedures 101
______Experimental Period 101
______Visit Scheduling Requirements 101
______Experimental Visits Weeks 1, 3, 5, 7, 9 and 11 Procedures 102
_________Measures/Assessments 102
______Experimental Visits Weeks 2, 4, 6, 8, 10 and 12 Procedures 102
_________Measures/Assessments 102
_________ Week 12 fMRI Testing 103
______Interactive Voice Response System 104
______Variable Incentive Program 104
______Product and Procedures Compliance Review Sessions 105
______Quit Attempts During the Study Protocol 105
_________If a Participant is Currently Abstaining from Smoking with the Intention to Quit 105
_________If a Participant is Planning to Quit Smoking, But Has Not Initiated Quit Attempt 106

Abstinence Assessment Session ... 106
Participants Who Meet Criteria for Abstinence ... 106
Measures/Assessments ... 106
Additional Tasks ... 107
Participants Who Do Not Meet Criteria for Abstinence ... 107
Measures/Assessments ... 107
Participant Compensation ... 107
End of Study ... 108
30 Day Follow up Phone Call ... 108
Randomization ... 109
Product Accountability ... 110
Statistical Methods and Sample Size ... 111
Potential Risks of Participation ... 111
Risks of Participation ... 111
Avoiding Risk to the Fetus ... 113
Expected Benefits of Participation ... 113
Study Debriefing ... 113
Protection Against Risk ... 113
Data Collection Protections ... 113
Data Storge ... 114
Adverse Events ... 114
Withdrawal or Monitoring of Participants ... 115
Data Safety Monitoring Board ... 116
Investigational Tobacco Product ... 117
Certificate of Confidentiality ... 13
Outcome Variables ... 118
References ... 119

Abbreviations

- VLNC: Very low nicotine content
- RNC: Reduced nicotine content
- NNC: Normal nicotine content
- CPD: Cigarettes per day
- CO: Carbon monoxide
- BAL: Breath alcohol levels
- BP: Blood pressure
- HR: Heart rate
- BPM: Beats per minute
- BMI: Body Mass Index
- NMR: Nicotine metabolite ratio
- NNN: *N′*-nitrosonornicotine
- NNAL: 4-(methylnitrosamino)-1-(3-pyridyl)-1-butanol
- BDI: Beck's Depression Inventory
- OASIS: Overall Anxiety Severity and Impairment Scale
- MINI: Mini International Neuropsychiatric Interview
- FTND: Fagerström Test for Nicotine Dependence
- WISDM: Wisconsin Index of Smoking Dependence Motives
- TLFB: Timeline Follow Back
- MNWS: Minnesota Nicotine Withdrawal Scale
- QSU: Questionnaire of Smoking Urges
- CES: Cigarette Evaluation Scale
- CPT: Continuous Performance Task
- IVR: Interactive Voice Response
- EDC: Electronic Data Capture
- CPT: Cigarette Purchase Task
- Brief-A: Behavioral Rating Inventory of Executive Function
- EQ-5D: Euro-Qol
- TPQ: Time Perspectives Questionnaire
- D-KEFS: Delis-Kaplan Executive Function System
- DDT: Delayed Discounting Task

- WASI-II: Wechsler Abbreviated Scale of Intelligence-II
- SST: Stop Signal Task
- FeNO: Fractional Exhaled Nitric Oxide
- 3 HC: 3-hydroxycotinine
- COT: Cotinine

Protocol

Objective:

The primary overall objective of these studies is to evaluate the effects of extended exposure to cigarettes differing in nicotine content in socioeconomically disadvantaged ($\leq$ high school educational attainment) women of childbearing age using a 3-condition, parallel groups design. After a baseline period in which daily smoking rate and other baseline assessments are completed, participants will be randomly assigned to one of three cigarette conditions (nicotine content: 0.04, 2.4, and 15.8 mg nicotine/gram of tobacco) for the 12-week experimental period.

Background Information:

The 2009 Family Smoking Prevention and Tobacco Control Act (FSPTCA) gives the Food and Drug Administration (FDA) regulatory authority over tobacco products, including nicotine levels in cigarettes. That is an exciting development as it creates the opportunity to examine the Benowitz and Henningfield (1994) hypothesis that smoking prevalence, nicotine dependence, and smoking-related morbidity and mortality can be lowered substantially by reducing the nicotine content of cigarettes to non-addictive levels. Computer modeling predicts that reducing nicotine levels in cigarettes would produce substantial improvements in population health (Tengs et al., 2005). An essential initial step towards the implementation of such a policy is to thoroughly investigate its safety and potential unintended adverse consequences. Indeed, the FDA's Center for Tobacco Products seeks to establish research centers to assist with the mission of investigating such regulatory matters related to the FSPTCA (see RFA-DA-13-003). The FDA explicitly notes that researching tobacco regulatory questions in vulnerable populations is a crosscutting agency priority, listing women of childbearing age (15-44) and pregnant women among the vulnerable populations of interest.

Approximately 23% of U.S. women of childbearing age (15-44 years) are current cigarette smokers (CDC, 2011). However, smoking is overrepresented among socioeconomically disadvantaged women, especially those with less education. For example, smoking prevalence rates are 43% and 36% among women with < 12 yrs or a high school education, compared to 28% and 16% among those with some college and undergraduate degrees (SAMHSA, 2010). Prevalence of nicotine dependence similarly varies by educational attainment, with rates among smokers being 63%, 57%, 44%, and 27% among women with < 12 yrs, high school, some college, and undergraduate degrees (SAMHSA, 2010). Of particular potential relevance to the topic of this application, preference for high nicotine yield (i.e., "full-flavor" brand) cigarettes also varies by educational attainment, with 64%, 49%, 35%, and 14% of women in these same educational categories endorsing that preference. Overall, these data underscore a robust and pervasive inverse association between educational attainment and smoking among women of childbearing age. In this application, we will focus on women who have $\leq$ 12 years of education, as this subgroup has the highest prevalence of smoking, nicotine dependence, and preference for cigarette brands with the highest nicotine yield (Kandel et al., 2009; SAMHSA, 2010).

Special Health Risks of Smoking Among Women

In addition to the adverse health consequences of smoking that cross genders, smoking also has adverse consequences specific to women's reproductive health. Women who smoke have an increased risk of cardiovascular disease, but women who smoke and use oral contraceptives

have a dose-dependent higher risk of heart attacks and strokes (WHO, 1997, 2010). Women who smoke also have increased risk of cervical cancer, infertility, and early menopause (Hughes & Brennan, 1996; Sun et al., 2012; US DHHS Surgeon General's Report, 2004; WHO 2004).

There is tremendous potential in this innovative public policy of reducing the nicotine content of cigarettes below an addiction threshold to reduce smoking prevalence and smoking-related disease and death in the US. However, a serious limitation of these studies that is directly relevant to this proposal is that they uniformly excluded vulnerable populations. This is an important gap in knowledge that must be addressed to comprehensively evaluate the Benowitz and Henningfield hypothesis. Understanding how smokers with psychiatric comorbidities and other vulnerabilities to smoking and smoking-related problems respond to reduced-nicotine cigarettes is essential for evaluating the potential impact of a nicotine reduction policy. This project represents the first investigation of reduced-nicotine cigarettes in smokers with mood and anxiety disorders and stands to contribute new scientific information with the potential to inform FDA policy decisions.

Cigarettes to be assessed in this study:

The cigarettes to be used in this study were made under an NIH contract with production being overseen by the Research Triangle Institute (referred to as "Spectrum cigarettes"). NIH currently has approximately 10 million of these cigarettes (of varying types) for research purposes. The cigarettes selected for the study span the range of yields likely to produce the hypothesized effects, as described above. The Spectrum cigarettes are not currently commercially available, although they are similar in many ways to marketed cigarettes (e.g., similar manufacturing, filter, paper, etc.).

The primary overall objective of this study is to evaluate the effects of extended exposure to cigarettes differing in nicotine content in female adult smokers of childbearing age (18-44 yrs) whose highest academic degree is high school using a 3-condition, parallel groups design. After a baseline period in which daily smoking rate and other baseline assessments are completed, participants will be randomly assigned to one of three cigarette conditions (nicotine content: 0.04 mg, 2.4 mg, and 15.8 mg nicotine/g of tobacco) for the 12-week experimental period.

Screening Procedures

Recruitment:

A sample size of 207 completers is proposed to test the primary outcome. Anticipating 25% attrition, and six pilot participants (3 at UVM, 3 at JHU), 282 participants will be enrolled across both sites (188 at UVM, 94 at JHU). Potential participants will respond to community advertisements (local newspapers, community bulletin boards, lab Facebook page, Facebook ads, lab website, center website, Craigslist, city buses, etc.) that contain a study description, link to an online survey and the name and phone number of the Research Assistant. Participants can choose to complete the pre-screening questionnaire online or by phone. If deemed eligible, those who complete the online questionnaire will be called by the Research Assistant to further discuss the study. The RA will read a script briefly explaining the study. Participants will be informed that this is not a smoking cessation program, and that smoking cessation services are available in the community independent of their decision to participate in this study. If interested,

they will be scheduled for an in-person screening interview. Those who call into the laboratory will be read a script briefly explaining the study. After verbal informed consent is received, the participants will be asked questions over the phone to determine initial eligibility. Callers will be informed that this is not a smoking cessation program, and that smoking cessation services are available in the community independent of their decision to participate in this study. If eligible and interested, they will be scheduled for an in-person screening interview.

Potential participants will be instructed to bring a pack of their usual brand cigarettes, all prescription medications they are currently taking and identification (example, driver's license) to the screening visit. If participants anticipate not having acceptable ID site staff should consult with the project coordinator or study PI.

A participant must complete her in-person screening session within 30 days of completing the pre-screening questionnaire. If the participant is not able to attend the in-person screening visit in that timeframe, she will need to complete the pre-screening questionnaire again.

Informed Consent Process:
Before beginning the informed consent process, potential participants will need to produce identification as described above. The interviewer will confirm the age and identity of the participant. If the participant is not between the ages of 18 and 44, she will be dismissed without payment. During the in-person screening session, study information will be presented and written informed consent will be required prior to participating in the screening session. In order to ensure adequate informed consent, participants will be asked to read the first several lines aloud (to determine literacy) and will then be given ample time to read the consent document. If the interviewer suspects the participant is not literate, he or she will have them continue reading further to confirm. Inability to read and comprehend written study materials will result in ineligibility and the interviewer will inform the participant that they are not eligible. Only after the participant and the researcher are fully satisfied that the participant understands the purpose of the study, the confidentiality of the data, the procedures, the risks/benefits and her rights as a research participant will the consent form be signed and the participant undergo screening procedures.

Screening Measures
Those who consent will be screened for eligibility using the following measures:

The following physiological measures will be collected, recorded on paper, and entered into REDCap by the interviewer at the end of the visit:

1) Breath alcohol levels (BAL) will be measured using an Alcosensor monitor. Participants with levels over 0.01 g/l may reschedule the interview but will need to be re-consented to ensure they have received adequate informed consent. They will be excluded if they are positive the second time.
2) Weight and height will be measured to determine the participant's Body Mass Index. Weight will be measured in kilograms and height will be measured in centimeters.
3) Expired breath carbon monoxide (CO) levels will be assessed using a Smokerlyzer ED50 CO meter (Bedfont Instruments), a reliable and valid measure of recent smoking.
    a. NicAlert Strips will be used to asses urinary cotinine levels if a participant's carbon monoxide reading is less than or equal to 8 ppm.

4) A urine toxicological screen will be performed to assess the presence of illicit drugs including marijuana, cocaine, opiates, oxycodone, benzodiazepines, barbiturates, amphetamines, methadone, buprenorphine, methamphetamines, MDMA and PCP. Participants who fail the drug screen for drugs other than marijuana may reschedule the interview but will need to be re-consented to ensure they have received adequate informed consent. They will be excluded if they are positive for drugs other than marijuana the second time. Urine Pregnancy Test (HCG detection) will be performed for all participants.
5) Blood pressure and heart rate will be measured using a CritiCare monitor to help the licensed medical professional determine final participant eligibility.

The following screening questionnaires will be participant-administered via paper and then will be entered into REDCap by the interviewer at the end of the visit:

1) Identifying Information Form will include the participant's REDCap Subject Identifier, name, address (including the county of residence), email address, phone number, age, date of birth, and social security number (if applicable).
    a. This form will be entered into the 'Identifying Information Access Database'.
        i. Each site will have a separate 'Identifying Information Access Database'.
        ii. Identifying information will not be shared with other sites. Each site is responsible for maintaining confidentiality of this information.
        iii. Identifying information will be kept in a locked file cabinet (source document) and in a password protected Access Database (electronic version) separate from all other study data.
2) Beck Depression Inventory (BDI; Beck, Ward, & Mendelson, 1961), to assess depressive symptoms.
3) Overall Anxiety Severity and Impairment Scale (OASIS; Norman et al., 2006) to assess frequency and severity of anxiety symptoms.

The following screening assessments will be administered as an interview and then will be entered into REDCap by the interviewer at the end of the visit:

1) The Mini International Neuropsychiatric Interview (MINI) suicide subscale (Sheehan et al., 1997) to evaluate suicide risk.
2) The Mini International Neuropsychiatric Interview (MINI) PLUS 6.0 Modules
3) MINI Follow-up Questionnaire (if applicable)
4) Tobacco Use History and Exposure Questionnaire, which measures variables such as smoking amount, cigarette brand, age of initiation of smoking, number of quit attempts, duration of quit attempts and duration of smoking.
5) Smoking Cessation Therapy Use Questionnaire
6) Time Since Last Cigarette Questionnaire
7) Medical History Questionnaire to assess current diagnoses, symptoms and past health problems.
    a. The medications section will be transferred onto the 'Concomitant Medications' form and entered into REDCap.

The following screening assessments will be completed by the participant directly in REDCap, except where noted:

1) Demographic History Questionnaire, which will assess age, gender, ethnicity, race, education, income, marital status, and employment history.
2) Alcohol Use Questionnaire (12 month and 1 month version)
3) Drug Use Questionnaire (12 month and 1 month version)
4) Fagerström Test for Nicotine Dependence (FTND; Heatherton et al., 1991)
5) Wisconsin Inventory of Smoking Dependence Motives-Brief Scale (WISDM; Piper et al., 2008), will be administered to assess nicotine dependence severity.
6) Smoking Stages of Change Algorithm as well as a contemplation ladder to assess intention to quit smoking (DiClemente et al., 1991).
7) The Mini International Neuropsychiatric Interview (MINI 6.0) (Sheehan et al., 1990) a structured diagnostic interview to evaluate psychiatric disorders.
    a. Will be completed by participant through the In-Home Screening system supported by Medical Outcomes Systems.

In the event that the REDCap website is not functioning, the assessments will be printed out and administered on paper. The source documents will be kept in the participant's binder. The interviewer will enter the data into REDCap when it resumes functioning properly. This information should be recorded in the 'End of Visit Evaluation Form' and filed in the participant's binder.

Suicidality/Mental Health Monitoring

Participants who endorse any suicidal ideation questions, indicate suicidal intention in the past month or a suicide attempt in the past 6 months as indicated on the as indicated on the BDI (score > 0 on question 9) or MINI suicide subscale (endorse question 3, 4 and/or 5 on the MINI suicide subscale or question 6 on the MINI suicide subscale with suicide attempt in the past 6 months) or answer "yes" to question A3g on the MINI Neuropsychiatric interview and symptoms have occurred in the past two weeks, will not be eligible to participate in the study. The research staff member will contact a licensed on-site clinician for evaluation. In the event that no clinician is available, staff will put the participant in contact with the National Suicide Prevention Lifeline at 1-800-273-8255. They will also contact the Study Coordinator and Site PI to inform them of the situation as soon as possible. Additionally, they will contact the Project Coordinator to inform her of the situation. The participant will be paid $25 (+$25 bonus if applicable) and provided with local mental health resources. Post enrollment, any report of suicidal ideation or attempt by a participant will be grounds for immediate withdrawal from the study.

Inclusion/Exclusion Criteria

Inclusion Criteria:

1) Women ages 18-44 years who have < an Associate's degree
2) Report smoking ≥ 5 cigarettes per day for the past year,
3) Provide an intake breath CO sample >8 ppm, (if ≤ 8 ppm, then NicAlert Strip > 2)
4) Be without current (within the past year) serious mental disorder that would interfere with study results or completion as determined by the licensed medical professional or PI,
5) Be without current substance abuse/dependence other than nicotine,
6) Be sufficiently literate to complete the research-related tasks,
7) Be in good physical health without serious illness or change in health or medication in the past three months as determined by the licensed medical professional at each site,

8) Not pregnant or nursing and report using oral, implant, patch, ring, IUD, injection or barrier contraceptives or report being surgically sterile, or post-menopausal,
9) Report no significant use of other tobacco or nicotine products within the past month (more than 9 days in the past 30).

Exclusion Criteria:

1) Any prior regular use (used as primary cigarette outside of the laboratory) of Spectrum cigarettes (i.e., research cigarettes with reduced nicotine content),
2) Exclusive use of roll-your-own cigarettes,
3) Planning to quit smoking in the next 30 days,
4) A quit attempt in the past 30 days resulting in greater than 3 days of abstinence,
5) Currently taking anticonvulsant medications including:
    a. Phenytoin [Brand Name: Dilantin]
    b. Carbamazepine [Brand Name: Tegretol, Carbatrol, Equetro, Epitol]
    c. Oxcarbazepine [Brand Name: Trileptal]
    d. Primidone [Brand Name: Mysoline]
    e. Phenobarbital
6) Positive toxicology screen for any of the following drugs: cocaine, opiates, oxycodone, methadone, buprenorphine, benzodiazepines, barbiturates, amphetamines, methamphetamines, MDMA and PCP
    a. Marijuana will be tested for but will not be an exclusionary criterion. Participants will be discouraged from using marijuana during the study.
    b. Participants with valid prescriptions for opiates, benzodiazepines, barbiturates or amphetamines will not necessarily be excluded.
    c. Participants failing the toxicology screen will be allowed to re-screen once. These participants will need to be re-consented before being rescreened to ensure they have received adequate informed consent.
7) Breath alcohol level > 0.01
    a. Participants failing the breath alcohol screen will be allowed to re-screen once. These participants will need to be re-consented before being rescreened to ensure they have received adequate informed consent.
8) Self-report of binge drinking alcohol (more than 9 days in the past 30 days, 4 drinks in a 2 hour period)
9) Systolic blood pressure < 90 or ≥ 160 mmHg
    a. Participants failing for blood pressure will be allowed to re-screen once.
10) Diastolic blood pressure < 50 or ≥ 100 mmHg
    a. Participants failing for blood pressure will be allowed to re-screen once.
11) Breath CO > 80 ppm,
12) Heart rate is greater than or equal to 115 bpm or less than 45 bpm
    a. Participants failing for heart rate will be allowed to re-screen once.
13) Currently seeking treatment for smoking cessation,
14) Have used nicotine replacement, bupropion or other pharmacotherapies as cessation aids in the past month (bupropion will be allowed for treatment of depression),
15) Current symptoms of psychosis, dementia or mania,
16) Suicidal ideation in the past month (score > 0 on the BDI question 9 or endorse question 3, 4 and/or 5 on the MINI suicide subscale),
17) Answer “yes” to question A3g on the MINI Neuropsychiatric Interview Major Depressive Episode Module and symptoms occurred within the past two weeks,

18) Suicide attempt in past 6 months (endorse question 6 on the MINI suicide subscale with suicide attempt in the past 6 months) or,
19) Participation in another research study in the past 30 days.
20) Co- habitation with any former research participant who was provided with Spectrum research cigarettes to smoke outside the lab.

Children under age 18 are excluded because they cannot legally buy cigarettes. Those with unstable medical, psychiatric, or medication conditions (as determined by the licensed medical professional) are excluded as these symptoms could affect a participant's ability to complete the study. Examples include but are not limited to the following: angina, stroke, heart attack which occurred since phone screening, blood clots in the arms or legs for which the individual is undergoing active medical treatment, cancer requiring active chemotherapy or radiation therapy, severe shortness of breath caused by conditions such as uncontrolled asthma, COPD, or arrhythmia, active untreated infection such as pneumonia, active untreated endocrine disorder such as hyperthyroidism. We will exclude those currently seeking smoking treatment and those who plan to quit in the next 30 days, as participation in this study may not lead to reductions in smoking. We will exclude pregnant or nursing women and women of reproductive potential who are unwilling to use acceptable forms of birth control throughout the study. We will also exclude anyone with current or recent alcohol or drug abuse problems as these factors could independently affect smoking behavior during the study. Individuals with baseline CO readings greater than 80 ppm, those with heart rate or blood pressure readings that are out of range (systolic: 90-159 mmHg; diastolic: 50-99 mmHg; HR: 45-114 bpm) and anyone who has attempted suicide in the past six months will be excluded from the study for safety concerns. Individuals who smoke 'roll your own' cigarettes exclusively will be excluded from the study because we will be unable to standardize their baseline smoking behavior. Individuals who have recently participated in a research study will be excluded as participation may have changed their smoking behavior, which may preclude a stable smoking baseline. Because participants are required to complete portions of the protocol independently, they will need to be able to independently read and comprehend the study materials.

Eligibility Determination:

The research assistant will review the entire screening assessment battery for initial eligibility determination, confirming the subject meets the above described inclusion/exclusion criteria. The final eligibility of the participant will be determined by a licensed medical professional (MD, DO, NP, PA, Master's prepared RN or CRN) at each site after reviewing the Medical History Questionnaire, BDI, Mini Neuropsychiatric Interview, and the MINI suicide subscale. The licensed medical professional may meet with a participant if available and think it necessary for eligibility determination. He/she will sign off on eligibility prior to the first baseline visit. If the licensed medical professional determines the participant is not medically eligible to participate in the study, has current symptomatology that would interfere with interpretation of the data or is unlikely to complete the study he/she will inform the research assistants who will contact the participant prior to the first baseline visit. The licensed medical professional will not need to review the medical history forms of participants who are not eligible for other, non-medical reasons.

If a participant fails the urine toxicology screen due to a prescription medication he/she is taking, then he/she will not be automatically excluded. The interviewer will make note of this when he/she submits the forms to the licensed medical professional for final eligibility determination.

Once all the screening procedures have been completed, researchers will pay participants $25 (+$25 bonus if applicable) for their time as long as they pass the drug and breath alcohol tests and meet the minimum requirements for carbon monoxide or NicAlert levels. Those participants who do not pass these tests or meet these requirements will be dismissed from the study without payment. Marijuana will be tested for but will not be an exclusionary criterion. If a participant does not pass the drug test but has a current, valid prescription that would explain the failed test he/she will not be automatically excluded and will still receive the visit payment. Participants who meet all other eligibility criteria, sans the medical criteria, will be scheduled for the first baseline visit.

At the end of the screening session, the researcher will complete the End of Visit Evaluation Form, which will be filed in the subject's binder. This will allow the researcher to make note of any problems encountered during the visit, to track which computers were used for which tasks, and to assess the truthfulness of the participant in regards to self-report of tobacco use.

Baseline Procedures

This study will use a one-week, two-session baseline period to collect baseline individual difference measures and monitor daily usual-brand smoking behavior. At Baseline 1, participants will be provided their usual brand cigarettes to smoke, equivalent to 150% of their daily smoking rate. A time line follow back (TLFB) will be used to assess the daily cigarette use for the past 7 days. Participants will be provided their usual brand cigarettes for the first seven days of the baseline period. If the baseline period extends past seven days, participants will need to purchase their own usual brand cigarettes. Use of a two session baseline period will ensure stability of daily smoking reports, reduce reactivity to the daily cigarette monitoring, and reduce participant burden. During the two baseline sessions, participants will complete subjective questionnaires, assessments of cognitive functioning, and smoking topography. Each visit will last approximately two to four hours. At the end of each baseline session, the researcher will complete the End of Visit Evaluation Form, which will be filed in the participants' binder. This will allow the researcher to make note of any problems encountered during the visit, to track which computers were used for which tasks, and to assess the truthfulness of the participant in regards to self-report of tobacco use.

<u>Visit scheduling requirements for baseline period:</u>

Participants will be required to schedule the Baseline 1 visit within 30 days of their screening visit. If a participant still wants to be in the study after 30 days, he/she will need to be re-screened. The participant will need to be re-consented but will maintain the original REDCap Subject Identifier. The ideal target window separating Baseline 1 and Baseline 2 is between 7 and 12 days. The minimum is 7 days and the maximum is 21 days. If the participant does not complete the visit within 21 days, then he/she will not be rescheduled and will be discontinued from the study.

<u>Measures/Assessments</u>

Physiological measures collected at Baseline 1, recorded on paper, and entered into REDCap by the interviewer at the end of the visit:

1) BAL
2) Weight

3) CO
4) Blood Pressure
5) Heart Rate
6) Urine Toxicology

The following questionnaires will be participant-administered via paper at Baseline 1 and then will be entered into REDCap by the interviewer at the end of the visit:

1) BDI
2) OASIS

The following assessments will be administered as an interview at Baseline 1 and then entered into REDCap by the interviewer at the end of the visit:

1) Concomitant Medications Form
2) Health Changes Questionnaire, which will assess any weekly health changes,
3) Time Since Last Cigarette Questionnaire

The following assessments will be administered at Baseline 1 and completed by the participant directly in REDCap:

1) Perceived Health Risks Rating (Hatsukami et al., 2010), a measure of the perceived addictive potential and other health risks associated with cigarettes
2) Respiratory Health Questionnaire, a measure of cough, shortness of breath and other respiratory symptoms
3) Minnesota Nicotine Withdrawal Scale (MNWS; Hughes & Hatsukami, 1986), a measure of nicotine withdrawal
4) Questionnaire of Smoking Urges-brief scale - Usual Cigarette (QSU; Cox, Tiffany, & Christen, 2001; Tiffany & Drobes, 1991), which measures the urge to smoke
5) Cigarette Evaluation Scale – Usual Cigarette (CES; Westman, Levin, & Rose, 1992), which measures responses to cigarettes (e.g., reward, satisfaction).
6) Intolerance for Discomfort Questionnaire - (IDQ; Sirota et al., 2013), assesses intolerance for the discomfort of smoking abstinence. The measure includes three subscales: physical discomfort, emotional discomfort and smoking withdrawal discomfort.

13) Cigarette Purchase Task – Usual Brand Version (CPT; MacKillop et al., 2008), a self-report analogue of a progressive-ratio schedule that measures the relative reinforcing efficacy of cigarettes by querying how many of that day's cigarette they would consume in a day at varying prices. This task will indicate whether prolonged VLNC cigarette use reduces cigarette demand and increases sensitivity to increases in cigarette costs.
14) Perceived Stress Scale - 4 item (PSS-4; Cohen, Kamarck, & Mermelstein, 1983), which measures the degree to which life situations are appraised as stressful.
15) Positive and Negative Affect Schedule (PANAS; Watson, Clark, & Tellegan, 1988), which measures symptoms of positive and negative affect.

Physiological measures collected at Baseline 2, recorded on paper and entered into REDCap by the interviewer at the end of the visit:

1) BAL
2) Weight
1) CO
2) Blood Pressure
3) Heart Rate
4) Urine Toxicology
5) Urine Pregnancy

The following assessments will be administered as an interview at Baseline 2 and then entered into REDCap by the interviewer at the end of the visit:

1) Concomitant Medications Form
2) Health Changes Questionnaire
3) Time Since Last Cigarette Questionnaire

The following assessments will be administered at Baseline 2 and completed by the participant on paper and entered into REDCap by the interviewer at the end of the visit:

1) BDI
2) OASIS

The following assessments will be administered at Baseline 2 and completed by the participant directly in REDCap:

1) FTND
2) WISDM

In the event that the REDCap website is not functioning, the assessments will be printed out and administered on paper. The source documents will be kept in the participant's binder. The interviewer will enter the data into REDCap when it resumes functioning properly. This information should be recorded in the 'End of Visit Evaluation Form' and filed in the participant's binder.

Cognitive Tasks (Baseline 2 Only):
Cognitive functioning will be assessed using a battery of computer-based assessments. We will assess domains that are theoretically linked to smoking and likely to be sensitive to nicotine abstinence (Heishman, 1999; Kleykamp et al., 2005; Rycroft et al., 2006). Prior to test administration, participants will be trained to ensure their understanding of each test. Tests will be administered on a desktop computer.

1) **N-Back (0,2) Task** (Ernst et al., 2001): A measure of working memory in which participants view serially presented letters on a computer. They must indicate whether each letter presented is the same or different from the letter presented a specified number of positions back in the string of letters (e.g. 2-back).
2) **2-Letter Search** (Ernst et al., 2001): A measure of focused attention in which participants view strings of letters on a computer screen looking for whether each string contains or does not contain two target letters.
3) **Continuous Performance Test** (CPT; Myers et. Al., 2008): A measure of sustained attention, participants must monitor a string of stimuli (e.g. letters) serially presented on

a computer screen monitoring for presentation of a target stimulus. The task is balanced so that they either must respond, or inhibit a response each time the target is presented.

4) **Stop Signal Task** (SST; Logan et al., 1984): A computer administered test of behavioral inhibition. Participants make frequent motor responses (e.g., left/right responses indicating if a visually presented arrow points left or right) and occasional, unpredictable response inhibitions (e.g., when a second arrow, pointing upwards, is presented). The stop signal delay (the interval between the onset of the go signal and stop signal) is adjusted after each stop trial according to the participants' performance to achieve 50 percent inhibition success rate.
5) **Nicotine Stroop Task** (Stroop, 1935): Frequently used measure of inhibitory control functioning. It measures the ability to focus attention on relevant stimuli while ignoring distracters and to suppress a prepotent response (i.e., word reading) in favor of an atypical one (i.e., color naming). Participants will be shown a number of images. The images will either be nicotine related, evocative, or neutral in nature with different color borders (red, blue, green yellow). The participants will be asked to use response triggers to identify the color of the border for each picture as they appear on the screen.

Smoking Topography (Baseline 2 Only):

Puff Topography, a precise measure of smoking behavior (Brauer et al., 1996; Herning et al., 1981; Robinson & Forbes, 1975), will be used to examine whether prolonged use of the experimental cigarettes affects topography measures that may indicate smoking compensation (Strasser et al., 2007). Puff topography will be assessed using a CReSS pocket device that provides a valid measurement of puff number, puff volume, inter-puff interval and other indices (Blank et al., 2009). Carbon monoxide readings will be collected before and 15 minutes after puff topography. Participants will smoke one cigarette of their usual brand.

Interactive Voice Response System:

At the end of the first baseline visit, participants will be trained to use the Interactive Voice Response (IVR) System, which will contact participants each day throughout the study and ask about their smoking behavior as well as withdrawal symptoms the week before and after Baseline 2. We will also review the IVR adherence incentive program, which consists of $1 per call plus a $10 bonus for seven consecutive calls. Participants will be provided a study cell phone if they have unreliable telephone access, do not have enough monthly cell phone minutes or prefer not to use their own phone.

The IVR system is operated by TeleSage. To be enrolled in the IVR system, research staff will enter the participants initials, telephone number, subject identifier, and visit dates into the IVR TCORS website. Identifying information (initials and telephone numbers) will not be extracted with the data by the bioinformatics group. Please refer to TeleSage's privacy statement and HIPAA compliance form for additional information.

Baseline 2 biological specimens:

1) <u>Urine sample for smoking biomarker assessment</u>**:**
   Participants will be asked to bring a urine sample (first void of the day) to the second baseline session for biomarker assessment. Samples will be stored at temperatures no more than -80°C. The tobacco-specific carcinogen biomarkers are total NNAL and PAH. Anatabine and anabasine will be tested in the VLNC condition to validate abstinence or measure the extent of nicotine replacement therapy being used. Total cotinine levels will also be assessed to measure daily nicotine exposure. Participant's will be reminded

with a phone call the day before the visit, those who forget will be asked to provide an onsite urine sample.

2) Pulmonary Marker:
Fractional Exhaled Nitric Oxide (FeNO) will be assessed as a measure of lung function using the NIOX VERO, a hand-held device for exhaled NO analysis. FeNO involves no storing or shipping of specimens, rather, the participant will exhale slowly through the device to obtain the result, which will be recorded in the participant's source.
3) Cardiovascular Markers:
Blood samples will be used for measurement of a battery of cardiovascular biomarkers primarily focusing on three areas: glucose tolerance (fasting insulin, glucose, hemoglobin A1C), clotting markers (thrombin, fibrinogen, PAI-1), inflammatory markers (C-reactive protein, interleukin-6, D-Dimer). Secondary measures include: Fasting lipid profile (total cholesterol, triglycerides, HDL-C, LDL-C). Participants will be required to fast for a minimum of 8 hours. Ideally, participants will not eat or drink after midnight and blood draws will be done in the morning. After the blood draw, participants will be provided with a meal voucher so that they may eat before performing the remaining visit tasks. The following volumes and tubes will be collected: Two 5 mL SST tubes, one 10 mL EDTA tube and two 2.7 mL citrate tubes.
4) Additional Blood Samples:
Blood samples will also be used for assessing individual differences in nicotine metabolism by phenotyping (i.e., Nicotine Metabolic Ratio, NMR, which is phenotypically estimated as the ratio of 3-hydroxycotinine [3 HC] to cotinine [COT] in plasma). One 10 mL EDTA tube will be collected.

We will store blood for the purposes of analyzing additional cardiovascular biomarkers or genotyping of individual differences in nicotine metabolism (CYP2A6) analyses of nicotine metabolism (variation in CYP2A6) or nicotinic acetylcholine receptor gene subtypes. All samples will be stored at the University of Vermont Tracy Lab.

Biomarker shipping and storage:
Biomarkers will be shipped quarterly to the University of Vermont Laboratory for Clinical Biochemistry Research (Tracy Lab). The Tracy Lab will serve as a central repository for all biomarker specimens and will be responsible for distributing specimens to the appropriate labs on a quarterly basis. Urine samples will be analyzed and stored at the University of Minnesota Hecht Lab. Cardiovascular Biomarkers will be analyzed and stored at the Tracey Lab. Additional blood samples for the purposes of phenotyping will be analyzed and stored at the University of Toronto Tyndale Lab.

Baseline fMRI testing (University of Vermont only):
Participants at the UVM site will complete the neuroimaging battery two or three days after the first baseline assessment, depending on availability. This battery will be completed only among a randomly selected subset of participants in the lowest and the highest dose conditions (45 participants/dose condition for total of 90 participants), which will provide the greatest likelihood of detecting differences between nicotine doses. Forty-five participants from each of the two conditions will be selected with the goal of having 20 completers from each of the doses. Participants who consent to neuroimaging and meet the eligibility criteria will be encouraged to abstain from smoking for approximately 24 hours before their scan. Abstinence will be verified by expired breath carbon monoxide levels that have decreased by at least 50% from the

measure taken during the Baseline 1 visit. The battery includes fMRI assessments that parallel the behavioral/cognitive assessments described above (i.e., a sustained attention task, inhibitory control test of executive function) and that are sensitive to abstinence-related disruptions in performance.

Prior to Baseline scan, participants will partake in a practice session of the fMRI cognitive battery tasks in a mock scanner at the Clinical Research Center (CRC) in order to practice each task in an environment that closely mimics that of the actual fMRI machine itself.

The neuroimaging battery also includes a high-resolution anatomical scan to assess total and regional grey matter volumes and cortical thickness, a resting- state scan to assess intra- and inter-regional brain connectivity, and arterial spin labeling to provide a quantitative measure of blood flow. Baseline characterization and comparison with a second scan approximately 12 weeks later will provide the potential for insights into the neurobiology of dependence and withdrawal (including individual differences in dependence severity) and differential changes that may arise from being exposed for an extended period to VLNC versus usual nicotine content levels in commercially available cigarettes.

Experimental Procedures

Experimental Period:

Participants will be seen weekly throughout the 12-week experimental period. Weeks 2, 6, 12 and the abstinence visit will take approximately 2-4 hours each. All other sessions will last approximately 2 hours. Upon arrival at the laboratory, participants will provide urine and breath BAL and CO samples. If the participant has a positive urine toxicology screen the Research Assistant will initiate the Field Sobriety SOP to determine if the participant can continue with the session or if it should be rescheduled. At the end of each experimental session, the researcher will complete the End of Visit Evaluation Form, which will be filed in the participant's binder. This will allow the researcher to make note of any problems encountered during the visit, to track which computers were used for which tasks, and to assess the truthfulness of the participant in regards to self-report of tobacco use and compliance to study procedures.

Visit scheduling requirements for experimental period:

The ideal scheduling window between each visit is 7 days based on the date of the Baseline 2 Visit. For additional scheduling requirements, refer to the '*Scheduling Visits SOP*'. If a participant misses a visit and is not able to reschedule during the window (± 3 days), that visit will not be 'made-up' in the future. All measures that were not completed will be considered missing data and will not be collected during future visits. If a visit mistakenly occurs outside of the designated window, this is a protocol deviation. A 'Non-Medical Event Form' will need to be completed. Additionally, each visit should occur at approximately the same time of day ± 2 hours.

If a participant is not able to attend his/her Week 12 visit, then it should be rescheduled even if it is outside of the scheduling window. This will be documented as a protocol deviation.

Experimental Visits Weeks 1, 3, 5, 7, 9, and 11 Procedures

Measures/Assessments

Physiological Measures Collected, recorded on paper, and entered into REDCap by the interviewer at the end of the visit:

1) BAL
2) Weight
3) CO
4) Blood Pressure
5) Heart Rate
6) Urine Toxicology

The following questionnaires will be participant-administered via paper at and then will be entered into REDCap by the interviewer at the end of the visit:

1) BDI
2) OASIS

The following assessments will be administered as an interview and will be entered into REDCap by the interviewer at the end of the visit:

1) Concomitant Medications
2) Medical Event Form, if applicable
3) Health Changes Questionnaire
4) Time Since Last Cigarette Questionnaire

The following assessments will be completed by the participant directly in REDCap:

1) MNWS
2) QSU brief - Usual Brand Cigarette
3) QSU brief - Study Cigarette
4) Cigarette Evaluation Scale - Study Cigarette

In the event that the REDCap website is not functioning, the assessments will be printed out and administered on paper. The source documents will be kept in the participant's binder. The interviewer will enter the data into REDCap when it resumes functioning properly. This information should be recorded in the 'End of Visit Evaluation Form' and filed in the participant's binder.

Experimental Visits Weeks 2, 4, 6, 8, 10 and 12 Procedures:

Measures/Assessments

Physiological measures collected, recorded on paper, and entered into REDCap by interviewer at the end of the visit:

1) BAL
2) Weight

3) CO
4) Blood Pressure
5) Heart Rate
6) Urine Toxicology
7) Urine Pregnancy test (if applicable

The following questionnaires will be participant-administered via paper at and then will be entered into REDCap by the interviewer at the end of the visit:

1) BDI
2) OASIS

The following assessments will be administered as an interview and will be entered into REDCap by the interviewer at the end of the visit:

1) Concomitant Medications
2) Medical Event Form, if applicable
3) Health Changes Questionnaire
4) Time Since Last Cigarette Questionnaire

The following assessments will be completed by the participant directly in REDCap:

1) Respiratory Health Questionnaire (weeks 2, 6 and 12 only)
2) FTND
3) Perceived Health Risks Questionnaire (weeks 2, 6 and 12 only)
4) Smoking Stages of Change Algorithm and Contemplation Ladder (Week 12 only)
5) Cigarette Purchase Task - Usual Brand Cigarette Version (weeks 2, 6 and 12 only)
6) Cigarette Purchase Task - Study Cigarette Version (weeks 2, 6 and 12 only)
7) WISDM-Brief
8) Drug Use Questionnaire - 1 month version (weeks 6 and 12 only)
9) PANAS (weeks 2, 4, 6, 8, 10 and 12)
10) Perceived Stress Scale (weeks 2, 6, and 12 only)
11) Alcohol Use Questionnaire - 1 month version (weeks 6 and 12 only)

In the event that the REDCap website is not functioning, the assessments will be printed out and administered on paper. The source documents will be kept in the participant's binder. The interviewer will enter the data into REDCap when it resumes functioning properly. This information should be recorded in the 'End of Visit Evaluation Form' and filed in the participant's binder.

Participants will also complete the following tasks:

1) Cognitive tasks (weeks 2, 6 and 12 only)
2) Smoking Topography - study cigarette (weeks 2, 6 and 12 only)

Week 12 fMRI testing (University of Vermont only):

Participants at the UVM site will also complete the neuroimaging battery again to assess changes after extended exposure to different doses. Participants who have initiated a quit attempt will not be asked to smoke prior to the scan. Participants willing to smoke the research

cigarettes will take two puffs 30 minutes prior to the scan. If the participant is unwilling to smoke the research cigarette, they will be allowed to smoke their usual brand.

Biological Samples to be collected:

1) First void urine sample (Weeks 6 and 12 only)
2) Blood Samples (Weeks 6 and 12 only)
3) Collect FeNO (Weeks 6 and 12 only)

Interactive Voice Response System:

Participants will continue to use the IVR system on a daily basis throughout the experimental period to record the number of study cigarettes smoked per day and use of non-study cigarettes. During the first week after Baseline 2, the IVR system will collect information about withdrawal symptoms.

Variable Incentive Program:

An incentive program has been developed with the goal of improving attendance at scheduled assessment sessions, compliance with using only study-provided tobacco products, and encouraging honest self-reports regarding all nicotine/tobacco use.

Briefly, participants will receive a total of five tickets for each weekly visit they attend after randomization (Visits 03-14, weeks 1-12). In total, participants could earn 60 valid tickets across the 12 visits. Participants will be instructed that these tickets correspond to attendance (one ticket), honest reporting (one ticket), and adherence to using only the assigned study product (three tickets). They will be further instructed that these tickets “could” be eligible for entry into a monthly drawing for prizes, but that only tickets that are “validated” will be eligible for prizes.

Since it is prohibitively expensive to test urine samples each week for each participant and because it is currently not feasible to detect with reasonable precision non-compliance based on biomarkers in the two higher nicotine group, we plan to only validate the attendance tickets. Hence, each participant who attends their regularly scheduled weekly session will have a total of five validated tickets entered into the monthly drawing.

To convey the message that we may be validating honest reporting and use of only study-provided products, we will collect a weekly urine specimen from participants. Further, in a bogus pipeline of sorts, participants will be instructed and that these urine specimens MAY be used to biochemically verify compliance to the study product by testing different nicotine and tobacco products found in the urine. Likewise, participants will also be instructed that their honesty ticket MAY be validated if their self-reported tobacco use matches what’s in their urine. So there is some minor deception involved, but technically we could conduct urine toxicology testing for both purposes. Hence, if the urine toxicology testing is presented as something that MAY be done for validation purposes, we feel that any deception is relatively minor. For scientific/economic reasons we are just electing to restrict validation to attendance. Nevertheless, we will debrief all participants upon the completion of the trial. We will inform them that the incentive program was based exclusively on attendance due to the relatively high cost of urine toxicology testing and other practical problems with shipping the urines for prompt testing.

Drawings will be conducted on the 1st of each month. Validation will be performed by staff who

have no participant interaction and are not blind to condition. Any ticket drawn will be eligible for an incentive as the only true contingency is for attendance. There will be no mention of the basis for earning incentives (i.e., whether the ticket was for attendance, honesty, adherence,). Participants will simply be informed that he or she earned an incentive from the drawing.

Each drawing will be independent (without replacement); consequently, some participants will not win a prize and others may win more than one during the study if more than one of their tickets is drawn. After confirming winners, the remaining tickets from each month will be discarded (i.e., tickets will only be entered into one drawing). The monthly prize amounts are detailed below.

We estimate based on the 2½ years we estimate it will take to complete this study, that participants will win an average of approximately $65 in prizes or an additional $5.50 per week per participant.

Grand Prize (1): $500 cash
Second Prize (1): $200 cash
Third Prize (5): $10 cash

Product and Procedures Compliance Review Sessions:
At each visit, Baseline 2 through Week 11, participants will be counseled about their use of the study cigarettes. Participants will be asked about any concerns or obstacles associated with use of the study cigarettes. The importance of honest self-reporting will be stressed. Participants will be told that they will not be penalized for use of other nicotine or tobacco products and that it is crucial for them to report any use of these products. If difficulties are encountered, participants will be asked why they think they are experiencing difficulties (e.g., taste, withdrawal symptoms) and to problem-solve how to deal with these difficulties in order to meet the protocol requirements. Additionally, participants will be counseled about their IVR completion, visit attendance, task engagement and product accountability. Refer to the '*Product and Procedures Compliance Review Sessions SOP*' for more information.

Quit Attempts During the Study Protocol:
At each weekly session, we will ask the participant if he/she is currently abstaining from smoking with the intention of quitting. If the answer is no, then we will also ask if he/she is planning to quit smoking prior to his/her next scheduled visit.

If a Participant is Currently Abstaining from Smoking with the Intention to Quit:

- Encourage participant to continue abstaining from smoking
- Schedule the participant for normal weekly visits, but no puff topography
- Provide the participant with the '*Clearing the Air*' manual and local smoking cessation resources
- Give the participant the option to take home study product rather than require him/her to take the product
- If the participant choses to take home the study product have him/her sign a form acknowledging that cigarette availability could be detrimental to the quit attempt. Recommend that he/she put the product "away" at home as to avoid unwanted cues to smoke.

- If the participant chooses not to take home the study product, have him/her contact the lab if he/she lapses and would like to pick up or be mailed the study product prior to his/her next visit.

If a Participant is Planning to Quit Smoking, but has not initiated the quit attempt:

- Ask if he/she has identified a target quit date and, if so, what that target date is.
- Provide the participant with the '*Clearing the Air*' manual and local smoking cessation resources.
- Provide the participant with the study product as usual. Recommend that on the target date he/she put the product "away" at home as to avoid unwanted cues to smoke.

Abstinence Assessment Session:
After the week 12 visit, participants will be required to come back for one additional visit the following day. During this visit, participants will have been encouraged to abstain from smoking until their next scheduled visit (approximately 24 hours later). The abstinence assessment session should be scheduled no less than 18 hours and no more than 30 hours after the Week 12 visit. Abstinence will be verified by expired breath carbon monoxide levels that have decreased to $\leq$ 4 ppm. This session will allow us to determine whether the experimental cigarettes have reduced the effects of abstinence on these measures relative to the control conditions. If the participant does NOT meet abstinence criteria, he/she will only receive $20 for the visit.

Measures/Assessments

Physiological measures collected, recorded on paper, and entered into REDCap by the interviewer at the end of the visit:

1) BAL
2) CO
3) Blood Pressure
4) Heart Rate
5) Urine Toxicology

The following questionnaires will be participant-administered via paper at and then will be entered into REDCap by the interviewer at the end of the visit:

1) BDI
2) OASIS

The following assessments will be administered as an interview and will be entered into REDCap by the interviewer at the end of the visit:

1) Concomitant Medications
2) Medical Event Form, if applicable
3) Health Changes Questionnaire
4) Time Since Last Cigarette Questionnaire

The following assessments will be completed by the participant directly in REDCap:

1) MNWS
2) QSU-brief - Usual Cigarette
3) QSU-brief - Study Cigarette
4) Cigarette Purchase Task - Usual Brand Cigarette Version
5) Cigarette Purchase Task - Study Cigarette Version
6) Cigarette Evaluation Scale – Usual Brand Cigarette Version

In the event that the REDCap website is not functioning, the assessments will be printed out and administered on paper. The source documents will be kept in the participant's binder. The interviewer will enter the data into REDCap when it resumes functioning properly. This information should be recorded in the 'End of Visit Evaluation Form' and filed in the subject's binder.

Participants will also complete the following task:

1) Cognitive tasks
2) University of Vermont only: Participants at the UVM site will also complete the neuroimaging battery again to assess changes after extended exposure to different doses.

Participants who do <u>NOT</u> meet abstinence criteria will be required to complete the following assessments:

1) BAL
2) CO
3) Blood Pressure
4) Heart Rate
5) Urine Toxicology
6) Concomitant Medications
7) Health Changes Questionnaire
8) Medical Event Form, if applicable
9) TLFB

Participant Compensation:
Participants will receive $25 for completing the screening visit, plus an additional $25 bonus for completing the visit on time as scheduled. Payment will be made regardless of enrollment as long as the participant passes the drug test, breath alcohol test, and meets the minimum requirements for carbon monoxide or NicAlert levels. Participants who do not pass these tests will be dismissed from the screening visit without payment, except in the event they can produce a prescription for the medication that caused them to fail the drug test. Participants will receive $100 for each of the shorter sessions (Baseline 1, Weeks 1, 2, 3, 4, 5, 7, 8, 9, 10, 11), $150 for each of the longer sessions (Baseline 2, Weeks 6 &12), up to $160 for the abstinence visit ($150 for the visit + up to $10 for the preference test), $20 for biochemical verification of abstinence, up to $221 for completing daily IVR reports of study cigarette and other nicotine and tobacco use. Participants will also have a chance to earn an additional $50 bonus for every three visits that are completed on time as scheduled. There will also be a $100 bonus for

completing the study for a total bonus of $325. If the participant does not attend the screening visit or one of the weekly visits as scheduled, they will forfeit the bonus. They will have a chance to earn another bonus payment with the next set of three visits. Participants who do not complete the entire study will receive compensation for the sessions that they do complete. UVM participants who undergo fMRI testing will receive an additional $150/scan. Total compensation for completing Study 2, including study visit payments, daily IVR calls and bonuses is $2301 (or $2601 if participating in the fMRI testing). Participants will also have a chance to earn additional money through the Variable Incentive program. As mentioned above, participants will have a chance to earn additional incentives each month for compliance, honesty and attendance, however, we anticipate that on average, participants will win approximately $150 in prizes.

End of Study:
After a participant has completed all study procedures and has been paid for participation the research assistant will read the following script and give the participant the *Clearing the Air Manual.*

"If you've reduced your smoking during this study, we encourage you to continue these reductions or even consider quitting. We would like to provide you with some resources should you decide to try to abstain from smoking (give "Clearing the Air" and hotline information). Please also feel free to consult with your physician and use any medications he/she deems appropriate. We will call you in approximately 30 days to ask about your smoking since leaving the study. There is no right answer and we know how difficult quitting can be. Please just answer honestly. The call will take less than 5 minutes. Thanks again for your participation."

The following assessments will be administered using REDCap:

1) End of Study Questionnaire

30 Day Follow up Phone Call:
Participants will receive a follow-up phone call between 25 and 35 days after the abstinence assessment session to assess their smoking patterns. The phone questionnaire will last less than five minutes. The questionnaire will ask if the participant is still smoking, how much and whether he/she has attempted to quit smoking since the end of the study. Participants will receive 5 variable incentive program lottery tickets for completing the call as compensation. Those who report abstinence will be invited to come in for biochemical verification and be compensated $40 for doing so. A urine sample will be collected to test urine cotinine levels. Additionally, any Medical Event Forms that remain open from the last session will be discussed. If the participant became pregnant during the study, this would have been recorded as a medical event. During this phone call, the research assistant will confirm her due date. This event will remain open until delivery. At that time the licensed medical professional will contact the participant to ask a few questions about the baby's health and will update the Medical Event Form.

Once a participant has completed all study procedures and all open events have been closed,

the PI will review the participant’s binder and sign a form indicating study completion for that participant.

Randomization

At the end of the Baseline 1 session, participants will be randomized into one of three cigarette conditions. Participants in each condition will be assigned cigarettes that match their menthol preference. Participants will be randomized, using block randomization, in equal number to the dose conditions, with randomization stratified by study site and menthol status. Each site will randomize participants until the total goal of 282 participants across both sites is reached, and no effort will be made to recruit a specific number of menthol and non-menthol smokers at each site.

**Condition**	**TPMF Code**	**Type***	**Specifications Nicotine Yield**	**Specifications Tar Yield**	**Specification Range for Nicotine Yield**	**Specifications Nicotine Content**
1	NRC600	CN	0.8 ± 0.15	9 ± 1.5	0.65 - 0.95	15.30 ± 0.18
1	NRC601	CN-Men	0.8 ± 0.15	9 ± 1.5	0.65 - 0.95	16.03 ± 0.47
2	NRC300	RN	0.12 ± 0.03	9 ± 1.5	0.09 - 0.15	2.27 ± 0.08
2	NRC301	RN-Men	0.12 ± 0.03	9 ± 1.5	0.09 - 0.15	0.104 ± 0.002
3	NRC102	RN	0.03 ± 0.01	9 ± 1.5	0.02 - 0.04	0.37 ± 0.01
3	NRC103	RN-Men	0.03 ± 0.01	9 ± 1.5	0.02 - 0.04	0.39 ± 0.00

*Legend:	
RN	Reduced Nicotine
RN-Men	Reduced Nicotine-Menthol
CN	Conventional Nicotine
CN-Men	Conventional Nicotine-Menthol

The lead statistician will create a randomization schedule for each of the two sites, amounting to 150% of expected enrollment at each site. The excess randomization codes will be used in the event that a site will have to enroll extra participants due to unexpectedly slow enrollment at another site. The nicotine doses will be identified by letter code and the number 2 (V2, W2, X2,

Y2) and only Administrative Core personnel with no participant contact will have the link between the statistician's letter code and dose assignments. The randomization schedules and the link between the alphabetic code and treatment assignment will be maintained securely by the Administrative Core. A second, sealed, copy will be secured in a separate building to protect against loss related to fire or other unforeseen events.

The University of Vermont will be responsible for removing all identifying information from cigarettes received from the Research Triangle Institute (RTI), labeling each carton with a blind code, assigning product using this blind code based on the randomization schedule being provided by the UVM Biostatistics Core, and shipping cigarettes to each site as needed based on recruitment. Each site will be responsible for tracking product received and distributed to participants, collecting unused product from participants, and returning unused cigarettes to UVM. The participants, investigators and study staff will not have knowledge of which product is given to a participant or whether different participants received the same or different product.

During the experimental period, participants will be provided with a 14-day supply of research cigarettes equivalent to 150% of their daily smoking rate. This rate will be calculated at Baseline 2 and will be an average daily smoking rate based on the IVR data for the first seven days of the baseline period. This will ensure adequate availability of cigarettes in the numerous locations participants may typically keep a supply (home, work, vehicle, etc.) as well as avoid expending the entire supply if they miss a scheduled visit. Participants will be instructed to use the research cigarettes for 12 weeks, at which point they are to discontinue product use.

If there is prior knowledge a participant will be missing a visit (i.e. planned vacation, laboratory closure, etc.), then the participant will be provided with an adequate supply of cigarettes to make up for the missed visit(s).   The participant will be given a 21-day supply if one visit is going to be missed and a 28-day supply if two visits are going to be missed.

Participants will be asked to refrain from use of other non-study cigarettes during the study period. If participants have to use another nicotine product, they will be told to use a non-combustible product (gum, patch, etc.). Additionally, they will be told there is not a penalty for use of non-study cigarettes, and that it is crucial for them to report any use of non-study cigarettes or other nicotine or tobacco products. Throughout the baseline and experimental periods, an Interactive Voice Response (IVR) system will be used on a daily basis to record the number of study cigarettes and non-study cigarettes used the previous day. During the baseline and first experimental week, participants will also answer daily IVR questions about their mood. Participants will be seen weekly for assessments. Brief standardized review sessions focusing on compliance with the study cigarettes and other study procedures will be provided at each visit. At the end of the 12-week trial, participants will undergo an assessment of withdrawal, craving, and cognitive function following a brief period of abstinence.

Product Accountability:

Participants will be required to keep track of all the cigarettes provided to them. Therefore, they will be instructed to return all unused cigarettes and empty cigarette packs to the laboratory each week. Research staff will complete the 'Product Accountability Log' with the participants. Any discrepancies in the product dispensed versus product returned will be discussed and

recorded in the log. Empty cigarette packs will not be saved. Unused cigarette packs will be re-distributed to the participants during Weeks 1-11. During Week 12, any remaining unused cigarettes returned by the participants will be collected by the research staff.

Participants who report running out of cigarettes prior to a scheduled weekly visit will be allowed to come in for an unscheduled visit to obtain more research cigarettes. If a participant has more than two unanticipated visits we will determine if a rate change is necessary. To determine this, we will look at the past two CO levels as compared to the Baseline 2 CO. If the CO trend is consistent with the self-report of smoking all of the allotted cigarettes then a rate increase will be granted. The participant will then receive cigarettes at a rate of 175% of their daily smoking rate. The maximum increase is 200% of their daily smoking rate. If participants lose more than two packs of cigarettes and require an unscheduled visit to the laboratory to supplement their supply, they will be told the next time they lose more than two packs they will have to wait until their next scheduled appointment to receive more cigarettes.

Statistical Methods and Sample Size

Statistical methods. See Statistical Analysis Plan at the end of this Supplemental document.

Sample size. Sample size for other analyses was determined using power analysis for hypothesis tests related to the Primary Aim of Study 2, specifically to detect a significant difference between the reduced-nicotine conditions and the high-nicotine yield condition in the primary endpoints, cigarettes per day (CPD) and urine cotinine, at the end of the trial. Donny et al. (2015) found a reduction of 4.52 CPD and 6.07 CPD among subjects smoking 2.4 mg/g and 0.04 mg/g cigarettes, respectively, compared to those smoking normal nicotine cigarettes. In addition, they reported a decrease of 0.59 and 0.39 in urine cotinine among those smoking these same RNC cigarettes, compared to those smoking NNC cigarettes. A sample size of 69 completers per condition will provide 90% power to detect similar differences in CPD and greater than 95% power to detect differences in urine cotinine, with a two-sided type I error rates of 0.02. The type I error rate reflects the Bonferroni correction needed to allow testing of all pair-wise comparisons. Regarding fMRI power, the analysis was based on the estimated effect size of 2.04 (Cohen's d) from the cortical activation differences previously observed between smokers and ex-smokers on the same inhibitory control task proposed here (Nestor et al., 2011). With 20 completers in each condition, there is 80% power at $p = 0.05$ to detect effects about half as large (Cohen d=0.91) between any two conditions.

Potential Risks of Participation

1) <u>Survey Questionnaires</u>: The interview will include questions about medical history, drug and alcohol use, and questionnaires about mood. Answering these personal questions could make the participant feel uncomfortable.
2) <u>Breach of Confidentiality</u>: The risk of the interview is loss of privacy if other people find out the results.
3) <u>Coercion</u>: Coercion is a possible risk due to monetary compensation for participating in these studies. The likelihood of this risk is low because the compensation is commensurate with the amount of time and effort required for these studies.
4) <u>Drug Testing</u>: A breach of confidentiality could occur and other people could learn of the participant’s drug use.

5) Obtaining blood pressure: The blood pressure cuff may cause minimal discomfort. In obtaining blood pressure, researchers may find out the participant has abnormal blood pressure.

6) Smoking Cigarettes: All cigarettes are detrimental to a person's health and can lead to significant medical problems including:
    m. Cardiovascular Diseases: Coronary heart disease, heart attack, stroke, peripheral vascular disease, reduced blood circulation, abdominal aortic aneurysm
    n. Respiratory Diseases: Emphysema, bronchitis, and chronic airway obstruction
    o. Cancers: Cancer of the lung, bladder, cervix, esophagus, kidney, larynx, mouth, pancreas, throat, and stomach; leukemia
    p. Metabolic Diseases: Type 2 Diabetes
    q. Other Health Risks Associated with Smoking: Including but not limited to infertility, lower bone density in postmenopausal women, and hip fracture in women
    r. Death

7) Smoking study cigarettes: In addition to the above medical problems, participants may experience some minor adverse health effects such as headaches or experience withdrawal symptoms, which are listed below. Due to the altered nicotine levels, there could be a change in their cigarette use including the manner in which they inhale the smoke. Smoking the study cigarettes does not provide any less risk than their usual brand cigarette and could pose increased health risks. Participants may also experience increases in levels of carbon monoxide, a gas from smoke.

8) Smoking Withdrawal: Participants may experience smoking withdrawal symptoms during this study. The symptoms can be uncomfortable but are typically of minimal risk. Smoking withdrawal symptoms include:
    a. Anger, irritability, frustration
    b. Anxiousness, nervousness
    c. Depressed mood or sadness
    d. Desire or craving to smoke
    e. Difficulty concentrating
    f. Increased appetite, hunger or weight gain
    g. Insomnia, problems sleeping or awakening at night
    h. Restlessness
    i. Impatience
    j. Constipation
    k. Dizziness
    l. Coughing
    m. Dreaming or nightmares
    n. Nausea
    o. Sore Throat

9) Returning to Regular Smoking: It is possible that if participants return to smoking their usual brand of cigarette at the end of the study they may experience mild and transient nausea, dizziness, and lightheadedness.

10) Risk to Fetus**:** Smoking during pregnancy can lead to miscarriage, preterm delivery, stillbirth, low birth weight, problems with the placenta, birth defects such as cleft palate, sudden infant death syndrome (SIDS), and early childhood behavioral problems.

11) Changes in blood pressure and/or heart rate: Smoking and nicotine can affect the cardiovascular system, which may result in changes in blood pressure and/or heart rate.

12) Exacerbation of psychiatric symptoms: Smoking and nicotine can affect a person's mood and emotions and are associated with psychiatric disorders including major depressive disorder, general anxiety disorder, bipolar disorder and eating disorders. Any changes in nicotine or cigarettes consumption could adversely affect psychiatric conditions.

13) MRI: The MRI scanner produces a loud banging noise and may be uncomfortable for people who become anxious in confined spaces. The presence of metal in or on a participant's body during an MRI scan can present a serious health risk. The MRI staff will ask participants in detail about any possible metal they may have in or on them. Regarding unexpected MRI findings, the participant will be informed of what was found. In addition, information about the incidental finding can be provided to the participant's primary doctor or the study team can refer them to an appropriate specialist. The costs for any care that would be needed to diagnose or treat an incidental finding would not be covered by the research study and would be the responsibility of the participant.

Avoiding Risks to Fetus:

If participants choose to be sexually active, they should use an appropriate "double barrier" method of birth control (such as female use of a diaphragm, or contraceptive sponge, in addition to male use of a condom) or the female should be using prescribed "birth control" pills, patch, ring, injections, or implants or intrauterine device (IUD). Participants will be tested for pregnancy every two weeks beginning at screening through the last study visit. If a participant becomes pregnant during the study, she will be withdrawn from the study. Approximately 30 days after being withdrawn or having a positive pregnancy test at the last study visit, the research staff will call the participant to confirm her due date. The licensed medical professional will follow-up with the participant after delivery to ask questions about the baby's health.

Expected benefits of participation:

There are no immediate benefits from participating in the study. The information obtained from this study may ultimately help the Food and Drug Administration decide how best to regulate tobacco products with the goal of improving public health.

Study Debriefing:

After data collection is complete, participants will receive a letter telling them which condition they were randomized into and the results of the study thus far.

Protection Against Risk

Research data without identifiers will be maintained in a locked file cabinet and on password-protected computers in the research staff workplace, with only code numbers identifying subjects. Study consent forms and the linkage between the participants' names and codes will be stored in a locked file cabinet. Interviews with participants will be conducted in private rooms. Urine samples for drug and pregnancy tests and tobacco exposure biomarkers will be obtained in a private bathroom within the laboratory suite. Blood draws will be performed in a private patient room. Subjective measures will be administered electronically. The biostatistics and data-management team will provide consistent data-management practices for all data in the Center. Validity and reliability of data will be maximized by using REDCap, which is housed on the Fletcher Allen Health Care, HIPAA compliant, computing system. REDCap is a secure, web-based system that accommodates local and remote data collection by each project team, and allows for data entry work-flow monitoring and data quality control monitoring by biometry

staff. For data integrity, data entry windows will follow the structure of paper forms as much as possible to allow for ease of entry, and will use predefined choices to minimize errors when possible. Data quality monitoring will be facilitated with periodic down loads and analysis using a variety of common statistical program format such as SAS, Stata, R, and SPSS. Quality control procedures will be conducted for all data collected, including analysis of missing data and logic checks for out of range and other anomalous values. This secure electronic data gathering and transmission plan, overseen by the experienced biostatistical team, will minimize opportunities for breaches of confidentiality. Biological samples for nicotine and carcinogen biomarker analysis will be marked with participant ID, stored in the locked laboratory suite, and sent to a laboratory for analysis on a quarterly basis.

All information collected as part of this study will be accessible only to research staff. No information will be shared with participants' clinicians unless the participant requests this in writing. All investigators and staff have undergone (and any new staff will undergo) human subjects' ethics training as required by UVM and are fully conversant with relevant ethical principals around confidentiality. Assessments, consenting and study procedures will be closely supervised by the PI.

The sponsors (NIDA/FDA) as well as the Institutional Review Board and regulatory authorities could be granted direct access to original medical and research records for verification of clinical trial procedures and/or data. If this is required, it will be done under conditions that will protect privacy to the fullest extent possible consistent with laws relating to public disclosure of information and the law-enforcement responsibilities of the agency.

Data Storage:
Data will be stored locally at each site, at the University of Minnesota Masonic Cancer Center's Bioinformatics Core and at the University of Vermont. Long-term storage of all study data, for at least 7 years after study completion, will be at the University of Vermont.

Adverse Events
The research assistant will ask about adverse events at each session, using a form that assesses the nature, severity, duration, action taken, and outcome of study-related adverse events. AEs will be captured from the time of first study cigarette. Participants will be given contact cards to inform us of events that occur between study contacts. Any AE that remains open will be reviewed and closed at an interview conducted 30 days after the study completion date (completers) or when the study should have ended had the participant completed the study (dropouts and those withdrawn by investigator).

All procedures will be monitored to ensure that they conform to the approved protocol. In addition, monitoring will be done of all unforeseen circumstances that might arise and affect safety; of all reports of serious adverse events as defined in 38 CFR 46 (death, new or prolonged hospitalization, persistent or significant disability/incapacity); of other significant adverse events (adverse events that lead to drop out by the participant or termination by the investigator); of unexpected adverse events resulting from the study, and of expected adverse events.

Any SAE will be brought to the attention of the site PIs as soon as possible and not longer than 24 hours. Any AE or SAE that is both unexpected and related to study participation will be reported to the IRB within 7 days of the event. The local IRB will make a determination as to whether additional reporting requirements are needed. IRB actions will be reported to the funding agency by the PIs no less than annually and more frequently as recommended by the local IRB. Any SAEs will be summarized in the yearly Progress Reports to the funding agency, including a review of frequency and severity. All SAEs will be followed through ongoing consultation with the physician caring for the patient until they resolve, result in death, or stabilize and are not expected to improve. The study staff will be in close contact with participants and health care providers throughout the study to monitor for potential unanticipated problems. Any unanticipated problems will be discussed at the weekly research staff meetings and reported as required to the local IRB.

Withdrawal or Monitoring of Participants

For the participant's protection, participants will be withdrawn immediately from the study if any of the following occur:

1) Cardiovascular disease (CVD) event: Typically includes MI (heart attack), PTCA (angioplasty/stenting), bypass surgery, stroke, peripheral vascular disease (arterial blockages in arms or legs leading to procedure or surgery). Less common CVD problems would be new cardiac arrhythmias (e.g., new atrial fibrillation) or new valvular disease (e.g., mitral or aortic regurgitation).
2) DVT/PE (deep vein thrombosis/pulmonary embolism, i.e., blood clots in the venous system).
3) Suicide Attempt: A participant will be withdrawn if he/she attempts suicide at any time during participation in the study.
4) Psychiatric Hospitalization: A participant will be withdrawn if he/she is hospitalized for psychiatric reasons at any time during participation in the study.
5) Pregnancy: If participant indicates she is pregnant or has a positive pregnancy test at any time during the study, she will be withdrawn from the study, and this event will remain open until delivery. At that time the licensed medical professional will contact the participant to ask a few questions about the baby's health and will update the open 'Medical Event Form'. A positive pregnancy test at Session 14 in Study 1 or Week 12 in Study 2 will trigger a 'Medical Event Form' to be completed but will not result in withdrawal since she is no longer receiving study product.
6) Expired breath carbon monoxide increase: A participant will be withdrawn from the study if the average of two consecutive CO readings during the same visit is 100 ppm or greater.
7) Marked increase in smoking: A participant will be withdrawn from the study if he/she meets **BOTH** of the following criteria for two consecutive weeks
    a. Cigarette per day increase: The average CPD increases by more than 100% from the average CPD during baseline.
    b. Expired breath carbon monoxide increase: If the average of two consecutive CO measurements in the same visit is
        i. CO is greater than 50 ppm if CO at Baseline 1 is <20 ppm.
        ii. CO is greater than 60 ppm if CO at Baseline 1 is 20 – 34 ppm.
        iii. CO is greater than 70 ppm if CO at Baseline 1 is 35 – 49 ppm.
        iv. CO is greater than 80 ppm if CO at Baseline 1 is 50 – 64 ppm.

    v. CO is greater than 90 ppm if CO at Baseline 1 is 65 – 80 ppm.

8) Note: If the second consecutive visit is the last study visit, then the participant would not be withdrawn from the study.

The following will be monitored and can lead to the participant being withdrawn by the PI or Licensed Medical Professional:

1) <u>Cigarettes per day increase</u>: Continued participation will be evaluated by the site PI if the average number of cigarettes per day (CPD) increases by more than 100% from the average CPD during baseline as determined by CPD at Baseline 2.
2) <u>Blood pressure (BP) or heart rate (HR) changes</u>: If any of the following occur post-enrollment: 1) BP is at or above 160/100 or below 90/50, or 2) HR is at or above 115 bpm or below 45 bpm a manual blood pressure and heart rate measurement will be taken after 10 minutes have passed. If the manual reading is still out of range, a 'Blood Pressure and Heart Rate Symptom Checklist' and 'Medical Event Form' will be completed, and the participant will be monitored by the medical professional.
3) <u>Expired breath Carbon Monoxide increase</u>: If the average of two consecutive CO measurements meets the criteria below then the 'Medical Event Form' will be completed and the participant will be monitored by the licensed medical professional.
    a. CO is greater than 50 ppm if CO at Baseline 1 is <20 ppm.
    b. CO is greater than 60 ppm if CO at Baseline 1 is 20 – 34 ppm.
    c. CO is greater than 70 ppm if CO at Baseline 1 is 35 – 49 ppm.
    d. CO is greater than 80 ppm if CO at Baseline 1 is 50 – 64 ppm.
    e. CO is greater than 90 ppm if CO at Baseline 1 is 65 – 80 ppm.
4) Any hospitalization or debilitation in which participation in the study could be detrimental to the recovery process. This will be self-reported by the participant and will be reviewed by the site PI and licensed medical professional to determine whether continued participation in the study is appropriate.
5) If a participant is behaving in an inappropriate or threatening manner, admits to lying about eligibility criteria, is participating in other smoking research studies that could affect the primary outcome measures, etc., then the PI can withdraw him/her from the study at the PI's discretion.
6) If a participant fails to attend regularly scheduled research assessment visits or comply with the research procedures or schedule, then the PI can withdraw him/her from the study at the PI's discretion.
7) <u>Increase in psychiatric symptoms:</u> Exacerbation in symptoms noted during the study (i.e., change in BDI category from mild to moderate or moderate to severe) will trigger review by the study's licensed medical professional. The PI will withdraw the participant upon the licensed medical professional's recommendation.

Data Safety Monitoring Board

A Data and Safety Monitoring Board (DSMB) has been established to monitor safety outcomes and will be comprised of five members. The DSMB will be chaired by Dr. Eden Evins, Associate Professor of Psychiatry at Harvard Medical School and Director of the Center for Addiction Medicine at Massachusetts General Hospital. Other members include: Kevin Delucchi, PhD., Professor in Residence of Biostatistics in Psychiatry at the University of California San Francisco and Director of the Quantitative Core of the San Francisco Treatment Research Center; Hendree E. Jones, Ph.D., Professor of Obstetrics and Gynecology and Director of UNC Horizons at University of North Carolina Chapel Hill; Wallace Pickworth, Ph.D., Research

Leader, Baltimore Operation, Centers for Public Health Research and Evaluation, Battelle; Kimber Richter, Ph.D., M.P.H., Associate Professor of Preventive Medicine and Public Health at the University of Kansas and Director of the University of Kansas Hospital's tobacco treatment program.

Conflict of interest

None of the members will be otherwise affiliated with the center and each member will complete a conflict of interest disclosure form prior to each meeting. Ad hoc specialists may be invited to participate as non-voting members at any time if additional expertise is desired.

Monitoring activities and frequency of meetings

The DSMB will set their own agenda and decisions about monitoring; e.g. how frequently to monitor, what threshold requires changes to protocol or stopping the study, and whether to view raw or analyzed data. The DSMB will be given FDA and EMEA guidelines for DSMBs and recent reviews on DSMBs. A brief report will be generated from each meeting for the study record and forwarded to each of the study site's Institutional Review Boards (IRB) and NIDA's Program Officer with the progress report. The DSMB will be available to convene outside of the regular meetings, if necessary. If concerns should arise regarding a particular subject, or any troublesome trends in the experiences of participants, they will make appropriate recommendations for changes in protocol, as needed. The project investigators will continue to examine safety data, blind to study condition, in case they wish to make study modifications. Before modifications are made, they will inform the DSMB and request their comments.

Communication plan to IRB, NIDA, and FDA (if applicable)

All IRBs, the FDA and the NIDA's Program Officer will be informed of any significant action taken as a result of the Data and Monitoring Board's findings. Study Participants will be informed of any changes in risk.

Protection of confidentiality

For DSMB meetings only de-identified data, including blinded study site and condition type, will be provided to the board. All data and discussion during the meeting will be confidential.

Investigational Tobacco Product

The Vermont Center on Tobacco and Regulatory Science has received an Investigational Tobacco Product (ITP) application from the FDA to cover the experimental cigarettes being used in this study. This application encompasses both trial sites.

Certificate of Confidentiality

To help protect the participant's privacy, Dr. Stephen Higgins, PhD, has received a Certificate of Confidentiality from the National Institutes of Health. With this certificate, the researchers cannot be forced to disclose information that may identify the participants, even by a court subpoena, in any federal, state, or local civil, criminal, administrative, legislative, or other proceedings. The researchers will use the Certificate to resist any demands for information that would identify the participants, except as explained below. The Certificate cannot be used to resist a demand for information from personnel of the United States Government that is used for auditing or evaluation of federally funded projects or for information that must be disclosed in order to meet the requirements of the Federal Food and Drug Administration (FDA).

The Certificate of Confidentiality does not prevent the participant or a member of their family from voluntarily releasing information about themself and their involvement in the research. If an insurer, employer or other person obtains the participant's written consent to receive research information, then the researcher may not use the Certificate to withhold that information.

The Certificate of Confidentiality does not prevent the researchers from disclosing voluntarily, without consent, information that would identify the individual as a participant of the research project in instances such as evidence of child abuse or a participant's threatened violence to self or others.

Outcome Variables

Primary Endpoints:

1) Total number of cigarettes smoked per day (CPD) during Week 12 is the primary outcome;

Secondary Endpoints:

1) Study CPD during Week 12, total and study CPD across weeks, simulated consumer demand
2) Measures of adherence: non-study cigarette use, drop-out rate
3) Measures of psychiatric symptoms: BDI, OASIS
4) Measures of discomfort/dysfunction: MNWS, QSU
5) Measures of other health-related behaviors: breath alcohol, urine drug screen, TLFB-drug use, Alcohol Use Questionnaire, Drug Use Questionnaire, weight
6) Measures of nicotine/tobacco dependence: FTND, WISDM
7) Measures of tobacco exposure: CO, total nicotine equivalents, NNAL, minor alkaloids
8) Measures of intention to quit: Stages of Change, Contemplation Ladder
9) Measures of compensatory smoking: puff topography, filter analysis
10) Measures of other tobacco use: TLFB-other tobacco
11) Measures of cigarette characteristics: CES
12) Measures of cognitive function: BRIEF-A, EQ-5D, TPQ, D-KEFS, WASI-II, DDT, SST
13) Measures of cardiovascular function: heart rate, blood pressure, urine 11-dehydroTXB2
14) Measures of perceived risk: Perceived Health Risk Questionnaire
15) Safety outcome variables: Adverse Events (AEs), Serious Adverse Events (SAEs)

References

Beck, A. T., Ward, C., & Mendelson, M. (1961). Beck depression inventory (BDI). *Archives of General Psychiatry, 4,* 561-571.

Benowitz, N. L., & Henningfield, J. E. (1994). Establishing a nicotine threshold for addiction. The implications for tobacco regulation. *New England Journal of Medicine, 331,* 123-125.

Blank, M. D., Disharoon, S., & Eissenberg, T. (2009). Comparison of methods for measurement of smoking behavior: Mouthpiece-based computerized devices versus direct observation. *Nicotine & Tobacco Research, 11,* 896-903.

Brauer, L.H., Hatsukami, D., Hanson, K., & Shiffman, S. (1996). Smoking topography in tobacco chippers and dependent smokers. *Addictive Behaviors, 21*(*2*), 233-238.

Centers for Disease Control and Prevention (CDC). (2011). Vital Signs: Current cigarette smoking among adults aged ≥ 17 years – United States, 2005-2010. *Morbidity and Mortality Weekly Report, 60,* 1207- 1212. Retrieved from http://www.cdc.gov/mmwr

Cohen, J. (1988). *Statistical power analysis for the behavioral sciences* (2nd ed.). New Jersey: Lawrence Erlbaum Associates, Publishers.

Cohen, S., Kamarck, T., & Mermelstein, R. (1983). A global measure of perceived stress. *Journal of Health and Social Behavior, 24(4),* 385-396.

Cox, L. S., Tiffany, S. T., & Christien, A. G. (2001). Evaluation of the brief questionnaire of smoking urges (QSU-brief) in laboratory and clinical settings. *Nicotine & Tobacco Research, 3,* 7-17.

DiClemente, C.C., Prochaska, J.O., Fairhurst, S.K., Velicer, W.F., Velasquez, M.M., & Rossi, J.S. (1991). The process of smoking cessation: an analysis of precontemplation, contemplation, and preparation stages of change. *Journal of Consulting and Clinical Psychology, 59(2),* 295-304.

Donny, E.C., Denlinger, R.L, Tidey, J. W., Koopmeiners, J. S., Benowitz, N. L.,Vandrey, R. G., … Hatsukami, D. K. (2015). Randomized trial of reduced-nicotine standards for cigarettes. *New England Journal of Medicine* 373(*Suppl* 14): 1340-1349.

Ernst, M., Heishman, S. J., Spurgeon, L., & London, E. D. (2001). Smoking history and nicotine effects on cognitive performance. *Neuropsychopharmacology, 25,* 313-319.

Hatsukami, D., Kotlyar, M., Hertsgaard, L. A., Zhang, Y., Carmella, S. G., Jensen, J. A., ... Hecht, S. S. (2010). Reduced nicotine content cigarettes: Effects on toxicant exposure, dependence and cessation. *Addiction, 105,* 343-55.

Heishman, S. J. (1999, September). Behavioral and cognitive effects of smoking: Relationship to nicotine addiction. *Nicotine & Tobacco Research, 1*(*Suppl* 2), S143–S147.

Heishman, S.J., Kleykamp, B.A., & Singleton, E.G. (2010). Meta-analysis of the acute effects of nicotine and smoking on human performance. *Psychopharmacology, 210*, 453–469.

Hughes, E. G., & Brennan, B. G. (1996). Does cigarette smoking impair natural or assisted fecundity? *Fertility and Sterility, 66,* 679-689.

Hughes, J. R., & Hatsukami, D. K. (1986). Signs and symptoms of tobacco withdrawal. *Archives of General Psychiatry, 43,* 289-294.

Kandel, D. B., Griesler, P. C., & Schaffran, C. (2009). Educational attainment and smoking among women: Risk factors and consequences for offspring. *Drug and Alcohol Dependence, 104(Suppl* 1), S24-S33.

Kleykamp, B. A., Jennings, J. M., Blank, M. D., & Eissenberg, T. (2005). The Effects of Nicotine on Attention and Working Memory in Never-Smokers. *Psychology of Addictive Behaviors, 19(4),* 433–438.

Logan, G. D., Cowan, W. B., & Davis, K. A. (1984). On the ability to inhibit simple and choice reaction time responses: A model and a method. *Journal of Experimental Psychology: Human Perception and Performance, 10(2),* 276–291.

MacKillop, J., Murphy, J. G., Ray, L. A., Eisenberg, D. T., Lisman, S. A., Lum, J. K., & Wilson, D. S. (2008). Further validation of a cigarette purchase task for assessing the relative reinforcing efficacy of nicotine in college smokers. *Experimental and Clinical Psychopharmacology, 16,* 57-65.

Myers, C. S., Taylor, R. C., Moolchan, E. T., & Heishman, S. J. (2008). Dose-related enhancement of mood and cognition in smokers administered nicotine nasal spray. *Neuropsychopharmacology, 33(3),* 588-598.

Nestor, L., McCabe, E., Jones, J., Clancy, L., & Garavan, H. (2011). Differences in "bottom-up" and "top-down" neural activity in current and former cigarette smokers: Evidence for neural substrates which may promote nicotine abstinence through increased cognitive control. *Neuroimage, 56,* 2258-2275.

Norman, S. B., Hami Cissell, S., Means-Christensen, A. J., & Stein, M. B. (2006). Development and validation of an overall anxiety severity and impairment scale (OASIS). *Depression and Anxiety, 23(4),* 245-249.

Piper, M. E., McCarthy, D. E., Bolt, D. M., Smith, S. S., Lerman, C., Benowitz, N., ... Baker, T. B. (2008). Assessing dimensions of nicotine dependence: An evaluation of the Nicotine Dependence Syndrome Scale (NDSS) and the Wisconsin Inventory of Smoking Dependence Motives (WISDM). *Nicotine & Tobacco Research, 10,* 1009-1020.

Substance Abuse and Mental Health Services Association (SAMHSA). (2011). *Results from the 2010 National Survey on Drug Use and Health: Summary of National Findings.* NSDUH Series

H-41, HHS Publication No. (SMA) 11-4685. Rockville, MD: Substance Abuse and Mental Health Services Administration.

Robinson, J. C., & Forbes, W.F. (1975) The Role of Carbon Monoxide in Cigarette Smoking. *Archives of Environmental Health. 30(9),* 425-434.

Rycroft, N., Hutton, S. B., & Rusted, J. M. (2006). The antisaccade task as an index of sustained goal activation in working memory: modulation by nicotine. *Psychopharmacology, 188(4),* 521-529.

Sheehan, D. V., Lecrubier, Y., Sheehan, K. H., Amorim, P., Janavs, J., Weiller, E., ... Dunbar, G. C. (1997). The validity of the Mini International Neuropsychiatric Interview (MINI) according to the SCID-P and its reliability. *European Psychiatry, 12,* 232-241.

Sirota, A. D., Rohsenow, D. J., Dolan, S. L., Martin, R. A., & Kahler, C. W. (2013). Intolerance for discomfort among smokers: Comparison of smoking-specific and non-specific measures to smoking history and patterns. *Addictive Behaviors, 38(3),* 1782-1787.

Strasser, A. A., Lerman, C., Sanborn, P. M., Pickworth, W. B., & Feldman, E. A. (2007). New lower nicotine cigarettes can produce compensatory smoking and increased carbon monoxide exposure. *Drug and Alcohol Dependence, 86,* 294-300.

Stroop, J. R. (1935). Studies of interference in serial verbal reactions. *Journal of Experimental Psychology, 18(6)*:643–662.

Sun, L., Tan, L., Yang, F., Luo, Y., Li, X., Deng, H. W., & Dvornyk, V. (2012). Meta-analysis suggests that smoking is associated with an increased risk of early natural menopause. *Menopause, 19,* 126-132.

Tengs, T.O., Ahmad, S., Savage, J.M., Moore, R., Gage, E. (2005). The AMA proposal to mandate nicotine reduction in cigarettes: a simulation of the population health impacts. *Preventive Medicine, 40,* 170-80.

Tiffany, S. T., & Drobes, D. J. (1991). The *development* and initial validation of a questionnaire on smoking urges. *British Journal of Addiction, 86,* 1467-1476.

U.S. Department of Health and Human Services (US DHHS). (2004). The Health Consequences of Smoking: A Report of the Surgeon General. Atlanta: U.S. Department of Health and Human Services, Centers for Disease Control and Prevention, National Center for Chronic Disease Prevention and Health Promotion, Office on Smoking and Health.

Watson, D., Clark, L. A., and Tellegen, A. (1988). Development and validation of brief measures of positive and negative affect: the PANAS scales. *Journal of Personality and Social Psychology, 54(6),* 1063.

Westman, E., Levin, E., & Rose, J. (1992). Smoking while wearing the nicotine patch: Is

smoking satisfying or harmful? *Clinical Research, 40,* 871A.

WHO. (1997). *Tobacco or health: A global status report.* Geneva, Switzerland: WHO Press.

WHO. (2010). *Gender, women, and the tobacco epidemic.* Geneva, Switzerland: WHO Press

WHO International Agency for Research on Cancer. (2004). *Tobacco smoke and involuntary smoking* [Monograph]. Lyon, France: WHO Press.

Statistical Analysis Plan

1. Trial Objectives

The primary objective of this study is to evaluate under double-blind conditions the effects of extended exposure to cigarettes varying in nicotine content in with affective disorders, opioid use disorder, and socioeconomically disadvantaged women of reproductive age. After a baseline period in which daily smoking rate and other baseline characteristics are assessed, participants will be randomly assigned to one of three cigarette conditions for the 12-week experimental period.

2. Trial Design

This is a randomized, multi-center, double-blind, parallel-groups design examining smokers using cigarettes containing 0.4 mg/g, 2.4 mg/g or 15.8 mg/g of nicotine. The cigarettes used throughout this trial are as follows:

Table 1. Description of cigarettes used in this trial.

Condition	TPMF Code	Type*	Specifications Nicotine Yield	Specifications Tar Yield	Specification Range for Nicotine Yield	Specifications Nicotine Content (mg/g)
1	NRC600	NNC	0.8 ± 0.15	9 ± 1.5	0.65 - 0.95	15.30 ± 0.18
1	NRC601	NNC-Men	0.8 ± 0.15	9 ± 1.5	0.65 - 0.95	16.03 ± 0.47
2	NRC300	VLNC	0.12 ± 0.03	9 ± 1.5	0.09 - 0.15	2.27 ± 0.08
2	NRC301	VLNC-Men	0.12 ± 0.03	9 ± 1.5	0.09 - 0.15	2.40 ± 0.03
3	NRC102	VLNC	0.03 ± 0.01	9 ± 1.5	0.02 - 0.04	0.37 ± 0.01
3	NRC103	VLNC-Men	0.03 ± 0.01	9 ± 1.5	0.02 - 0.04	0.39 ± 0.00

*Legend:	
VLNC	Very Low Nicotine Content
VLNC-Men	Very Low Nicotine-Menthol Content
NNC	Normal Nicotine Content
NNC-	Normal Nicotine-Menthol

Men	Content

The 15.8 mg/g dose most closely mirrors the dose level of conventional cigarettes, and will serve as the comparator of interest, unless otherwise specified.

2.1. Condition Assignment/Randomization

The study consisted of two baseline assessments, separated by one to three weeks, 12 weeks of extended exposure to research cigarettes, with assessments completed weekly, an abstinence assessment the day following the Week 12 visit and a final assessment 30 days after completing the study.

Participants were randomly assigned to one of the three nicotine doses, with randomization stratified by site and menthol preference. The nicotine doses were identified by letter code specific to nicotine dose and vulnerable population, and only Administrative Core personnel with no study-participant contact had the link between the statistician's letter code and dose assignments.

Participants were recruited from the University of Vermont (UVM: all three vulnerable populations), Johns Hopkins University (JHU: women of childbearing age and individuals with opioid use disorder) and Brown University (individuals with affective disorder).

The final randomization sequences were generated August 10, 2016, but had to be revised twice because of an unforeseen shortage of certain doses of the research cigarettes. The randomization required two major types of assumptions: 1) the proportion of participants recruited from each site, and 2) the proportion of recruited participants at each site who smoke menthol/non-menthol cigarettes. The recruitment goal for this study is 282 people in each vulnerable population, with the assumption that 207 of these participants will complete the study.

2.1.1. Condition Assignment/Randomization Adjustments

The initial randomization plan required equal numbers of participants to be randomized to each dose condition. After this study began, we were informed that three of the six cigarettes used in this study would be unavailable for a period of time (the 2.4 mg/g non-menthol [NRC 300] and 15.8 mg/g doses in non-menthol and menthol dose [NRC 600 & 601]). At that point, a revised randomization schedule was developed in order to continue with the study. The revised schedule took into consideration the distribution of menthol and non-menthol smokers at each of the study sites, as well as the supply of cigarettes on-hand at each site and projected cigarette availability. For the non-menthol smokers, this required that we alter the schedule to overemphasize the 0.4 mg dose condition, while for the menthol smokers, an equal number of participants were

randomized to each of the two doses that were readily available, with a limited number of participants randomized to receive the dose in short supply. Randomization using the revised schedules began about seven months into recruitment and was implemented for a period of approximately nine months. Participants were entered into the study over a period of 2.7 years.

When the three types of cigarettes again became available, the randomization schedule was revised again in order to distribute the remaining participants to achieve comparable sample sizes at the end of the study. For menthol smokers, fewer participants were randomized to each of the two doses that had been readily available, and a higher number randomized to receive the dose whose supply was previously limited, essentially reversing the ratio of randomization to study doses that had been in effect for the first adjustment. Because we were advised that one of the non-menthol cigarette doses would be available but insufficient for our estimated remaining needs, an alteration in the dose assignment ratios for non-menthol smokers was developed to mitigate, as much as possible, a projected imbalance in sample sizes per group. As with the menthol smokers, fewer non-menthol smokers are randomized to the dose that had originally been most readily available, with randomization to the two remaining doses emphasizing the dose which was now readily available. This newly revised randomization schedule was in effect until randomization to the three experimental groups was approximately equivalent, at which point the original randomization scheme was again put in place.

2.2. Sample Size

Sample size was determined using a power analysis for hypothesis testing of the primary study aim of the extended exposure study, specifically to detect a significant difference between the VLNC conditions and the NNC condition in the primary endpoints, average cigarettes/day (CPD) in Week 12 independent of adherence. Donny et al. (2015) found a reduction of 4.52 CPD and 6.07 CPD among participants smoking 2.4 mg/g and 0.4 mg/g cigarettes, respectively, compared to those smoking NNC. A sample size of 69 completers per condition for each vulnerable population will provide 90% power to detect similar differences in CPD, with a two-sided type I error rate of 0.02. The type I error rate reflects the Bonferroni correction needed to allow testing of all pair-wise comparisons. The recruitment estimate was increased to 282 participants per vulnerable population to accommodate the anticipated 26% loss to follow-up over the course of the trial.

3. Study Populations

For data analyses, the full analysis population included participants completing the randomization process. Participants were analyzed based upon the dose to which they were assigned, regardless of protocol violations and/or compliance to condition assignment. Participants not completing the study were excluded in the analysis of covariance models

based on the data collected at Week 12, while all participants were included in analyses examining changes over time.

4. Trial Endpoints

4.1. Primary Endpoints

The Primary Aim of the extended exposure study is to compare the effects of cigarettes varying in nicotine yield on smoking rate. The primary endpoint is average CPD during Week 12.

- CPD was collected by daily Interactive Voice Response (IVR) to assess cigarette use in the days since the last interview. This yields a continuous record of cigarette use throughout the study. A weekly average was obtained for analysis by averaging the daily CPD reports.

4.2. Secondary Endpoints

Additional measures included breath carbon monoxide (CO) level, smoking topography, urinary cotinine concentration, nicotine dependence (Fagerström Test for Nicotine Dependence, Wisconsin Index of Smoking Dependence Motives) scores, nicotine withdrawal (Minnesota Nicotine Withdrawal Scale, Questionnaire of Smoking Urges), and the Cigarette Purchase Task (CPT).

- Breath CO
    - Collected at baseline and weekly for the duration of the study
- Smoking topography
    - Collected at the second baseline and Weeks 2, 6, and 12
    - Puff frequency
    - Puff volume
    - Inter-puff interval
    - Number of puffs
    - Puff velocity
- Urinary cotinine concentration
    - Collected at the second baseline, Weeks 6 and 12
- Nicotine dependence and withdrawal
    - Collected at screening, the first baseline, even weeks until study completion and the abstinence visit
- CPT
    - Collected at the second baseline and Weeks 2, 6, 12 and the abstinence visit for usual brand cigarette
    - Collected at Weeks 2, 6, and 12 and the abstinence visit for study cigarette

- Breakpoint: the lowest price at which cigarette consumption is 0.
  - Elasticity of demand: the sensitivity of cigarette consumption to price increases.
  - Omax: the maximum daily expenditure for cigarettes.
  - Pmax: the price at which cigarette expenditure is maximized.
  - Intensity: cigarette consumption at the lowest price ($0 cost).
- Measures of depression and anxiety
  - Collected at the second baseline and weekly for the remainder of the study
  - Beck Depression Inventory
  - Overall Anxiety Severity and Impairment Scale
- Adherence to assigned tobacco products
  - Collected through the daily IVR system
- Use of multiple nicotine products.
  - Collected through the daily IVR system
- Biomarkers of exposure to tobacco carcinogens
  - Collected at the second baseline and Weeks 6 and 12
- Study retention
- Quit attempts and spontaneous quitting

  Assessed at weekly visits and at the 30-day post-intervention assessment

4.3. Exploratory Endpoints

- Markers of thrombotic risk and lung function
  - Collected at the second baseline and Weeks 6 and 12
- Cognitive tasks
  - Collected at the second baseline and Weeks 2, 6, 12 and the abstinence visit
- Neuroimaging data
- Preference test

5. Statistical Analysis

5.1. General Approach

The general approach to statistical analysis is based on a general linear mixed model, which allows the inclusion of a random effect for study site, a random participant effect (between-subject error), and a random error (within-subject error), when appropriate. Variance parameters were estimated using restricted maximum likelihood method. In the event of a statistically significant condition effect, post-hoc tests were conducted in order to explore the nature of the significant findings. First, the lowest nicotine content condition was compared to the NNC condition (15.8 mg/g); if significant differences were observed (p <

0.05), all pairwise comparisons were conducted using a Bonferroni multiple comparison adjustment.

This study was conducted in three different vulnerable populations under a similar protocol, with differences between protocols consisting of data collection specific to that vulnerable population. This included information such as use and timing of opioid maintenance therapy for individuals with opioid-use disorder or additional assessments of anxiety and depression for individuals with affective disorders. In order to explore potential differences across individuals with different vulnerabilities, data from all three studies were combined for analysis. A vulnerable population-by-condition or population-by- condition-by-time interaction term was included in all analyses. In the event that these interaction terms were statistically significant, all pairwise comparisons were conducted using a Bonferroni multiple comparison adjustment.

Transformations (including log, square root, etc.) were performed as needed so that ANOVA model assumptions of normality and equal variances hold. Geometric means in original units were calculated as well. Note that not all outcomes are assessed at weekly in-person visits. The time points at which each outcome is assessed is described in Section 6.

5.2. Describing the Study Population

5.2.1. Baseline Characteristics

Baseline characteristics, including demographics and smoking characteristics, were compared among conditions to identify any imbalances after randomization. Discrete variable are summarized by frequencies and percentages and compared using the Chi-squared Test or Fisher's Exact Test, as appropriate. Continuous variables are summarized by the mean and standard deviation, and compared using a one-way analysis of variance (ANOVA). These analyses include a random effect for study site. In the event that statistically significant differences are found, these baseline characteristics are included as fixed effects in all subsequent analyses.

5.2.2. Randomization Effects

While we do not expect participant characteristics or responses to change according to the time of entry into this study, we examined the effect of randomization scheme on baseline characteristics, with the independent variable being the randomization scheme in place at the time of study entry, categorized as original, first adjustment, and second adjustment, including a random effect for study site. In the event that statistically significant differences in any of these variables were found, they were to be included as fixed effects in all subsequent analyses. As menthol status was the only variable that differed because of the randomization scheme, it was included as a covariate in all analyses.

5.3. Primary Endpoint Analysis

5.3.1. Primary Analysis

The primary endpoint was differences among dose conditions in total CPD during Week 12. Data analysis is an analysis of co-variance, with condition as the independent variable, and the baseline value of that variable included as a covariate. In addition, vulnerable population, sex, age, and menthol status were included as fixed effects and study site as a random effect.

5.3.2. Secondary Analysis

As noted above, the CPD outcomes were assessed daily using the IVR system, with results reported as the mean CPD averaged over seven days. A growth curve was used to examine the trajectory of changes over the twelve weeks of the trial. Variance parameters were estimated using the restricted maximum likelihood method, assuming an unstructured covariance matrix. The models contained dose condition, time, vulnerable population, and the three-way interaction of vulnerable population-by-condition-by-time, as well as all appropriate two-way interactions. In addition, the baseline CPD was included as a covariate as well as sex, age, and menthol status. In the event that the three-way interaction was statistically significant, post-hoc tests were used to examine the exact nature of the differences, as outlined above. If the three-way interaction was not statistically significant, it was dropped from the model, and the analysis proceeded examining each of the two-way interactions in turn, with the primary effect of interest being the condition-by-time interaction. If none of the interactions were statistically significant, all were eliminated from the model, and the analysis was repeated, limited to the main effects of condition and time, as well as the covariates noted above.

5.4. Secondary Endpoint Analysis

The majority of secondary endpoints were examined in a manner similar to that described for primary endpoints described above. For outcomes measured weekly or every two weeks, a growth curve was used to examine the trajectory of changes over the twelve weeks of the trial. Variance parameters were estimated using the restricted maximum likelihood method, assuming an unstructured covariance matrix. For outcomes measured less frequently, repeated measures analysis of variance (ANOVA) was used using the restricted maximum likelihood method, with the structure of the covariance matrix assume to be compound symmetric. The choice of this structure was based on the value of the fit statistics, and the same covariance structure was assumed for all repeated measures ANOVA models, unless the model failed to converge. In that case, alternate assumptions were used to obtain a final model.

All models contained dose condition, time, vulnerable population, and the three-way interaction of vulnerable population-by-condition-by-time, as well as all appropriate two-way interactions. The baseline value of that particular variable was also be included as a covariate in the model, when available, as well as sex, age, and menthol status. Interactions were deleted from the model as describe above for the Primary Endpoint Analysis.

Outcome variables for which a transformation would not result in normally distributed data were also examined, including the number of days participants were abstinent throughout the 12-week trial and whether participants were able to maintain abstinence at the abstinence visit following the Week-12 visit, or whether they attempted to quit either throughout the trial or at the 30-day follow-up. Zero-inflated negative binomial regression was used to examine the number of days on which participants reported smoking no study or non-study cigarettes. The baseline CPD was included as a covariate in this analysis, along with vulnerable population, sex, age and menthol status. Logistic regression was used to examine the abstinence and quit attempts, with the models including vulnerable population, sex, age and menthol status as covariates.

5.5. Missing Data

We tested the pattern of missing values using Little's MCAR test, with the results suggesting that data were missing at random. Thus, we employed statistical procedures based on maximum likelihood estimates, which allows the inclusion of all subjects without imputation of missing values. The maximum likelihood approach estimates the parameter values that would maximize the probability of observing the data collected. In the event of missing variables, the likelihood for a given individual is the probability of observing the non-missing variables. Thus, the maximum likelihood approach allows the use of data from participants for the time period for which data is available, but not for time periods for which the data is missing. This procedure uses information from earlier time periods to estimate the effects of later time periods, while also accounting for the uncertainty of the projection in the computation of standard errors and test statistics.

While maximum likelihood estimation is considered superior to imputation methods for the treatment of missing data in clinical trials, additional analyses were conducted for the primary outcomes of total and study CPD at the 12-week time-point using multiple imputation with the Markov Chain Monte Carlo (MCMC) method carried out in PROC MI in SAS. A total of 100 imputed data sets were generated, with the condition effect assessed in each imputed data set. A final single assessment of experimental condition differences was obtained by combining the results across the imputed datasets using PROC MIANALYZE in SAS. The results of these analyses were compared to the primary analysis to evaluate the robustness of our conclusions.

6. Safety

Safety data included the number of adverse events, classified as to category, based on the Medical Dictionary for Regulatory Activities (MedDRA). In addition to the type of event, adverse events were categorized by severity and whether the adverse event was related to the study products/procedures.

Summary frequencies were provided based on the overall number of events as well as separately by dose condition. In addition to the number of events of a particular type recorded, the percent of adverse events were computed with the denominator being the number of participants in the safety population. Thus, the description of events are reported as events/person. The number of adverse events was compared across conditions using zero-inflated negative binomial regression, including a random effect for study site.

7. Interim Analyses

No interim analyses were conducted.

8. Reporting Conventions

P-values less than 0.05 were considered as statistically significant. Statistically significant p-values greater than or equal to 0.001 are reported to three decimals places; those less than 0.001 will be reported as <0.001. Non-significant p-values are reported to two decimal places. The mean, standard deviation, standard error, and other statistics are reported to one decimal place greater than the original value. Quantiles such as median, minimum and maximum will use the same number of decimal places as the original data. Estimated parameters not on the same scale as the raw observations, such as regression coefficients, are reported to two significant digits.

For the most part, statistical analyses were performed using SAS, version 9.4. Data management and cleaning were performed using tools available in REDCap, R and SAS, version 9.4.
