# Supplementary material for: Changes in Cigarette Consumption With Reduced Nicotine Content Cigarettes Among Smokers With Psychiatric Conditions or Socioeconomic Disadvantage: 3 Randomized Clinical Trials
Source: JAMA Netw Open. 2020 Oct 20;3(10):e2019311. doi: 10.1001/jamanetworkopen.2020.19311 (PMC7576411; doi:10.1001/jamanetworkopen.2020.19311)
Supplement: Supplement 2. — eFigure 1. Nonstudy Cigarettes Per Day eFigure 2. Wisconsin Index of Smoking Dependence and Motivation eFigure 3. Effects of Dose and Time on Amplitude and Persistence of Demand From the Cigarette Purchase Task eFigure 4. Questionnaire of Smoking Urges: Factor 1 eFigure 5. Questionnaire of Smoking Urges: Factor 2 eFigure 6. Beck Depression Inventory Score by Dose eTable 1. Demographic and Smoking Characteristics by Completor Status eTable 2. Participant Inclusion Criteria by Study Population eTable 3. Participant Exclusion Criteria by Study Population eTable 4. Minnesota Tobacco Withdrawal Scale Total Score by Dose eTable 5. Smoking Topography and Before and After Smoking Carbon Monoxide Changes eTable 6. Total Number of Adverse Events eTable 7. Number of Participants with Any Adverse Events eTable 8. Count of Serious and Severe Adverse Events eTable 9. Description of Serious and Severe Adverse Events [file jamanetwopen-e2019311-s002.pdf]

Supplemental Online Content

Higgins ST, Tidey JW, Sigmon SC, et al. Changes in cigarette consumption with reduced nicotine content cigarettes among smokers with psychiatric conditions or socioeconomic disadvantage: 3 randomized clinical trials. *JAMA Netw Open*. 2020;3(10):e2019311. doi:10.1001/jamanetworkopen.2020.19311

eFigure 1. Nonstudy Cigarettes Per Day

eFigure 2. Wisconsin Index of Smoking Dependence and Motivation

eFigure 3. Effects of Dose and Time on Amplitude and Persistence of Demand From the Cigarette Purchase Task

eFigure 4. Questionnaire of Smoking Urges: Factor 1

eFigure 5. Questionnaire of Smoking Urges: Factor 2

eFigure 6. Beck Depression Inventory Score by Dose

eTable 1. Demographic and Smoking Characteristics by Completor Status

eTable 2. Participant Inclusion Criteria by Study Population

eTable 3. Participant Exclusion Criteria by Study Population

eTable 4. Minnesota Tobacco Withdrawal Scale Total Score by Dose

eTable 5. Smoking Topography and Before to After Smoking Carbon Monoxide Changes

eTable 6. Total Number of Adverse Events

eTable 7. Number of Participants with Any Adverse Events

eTable 8. Count of Serious and Severe Adverse Events

eTable 9. Description of Serious and Severe Adverse Events

This supplemental material has been provided by the authors to give readers additional information about their work.

© 2020 Higgins ST et al. *JAMA Network Open.*

eFigure 1. Nonstudy Cigarettes Per Day

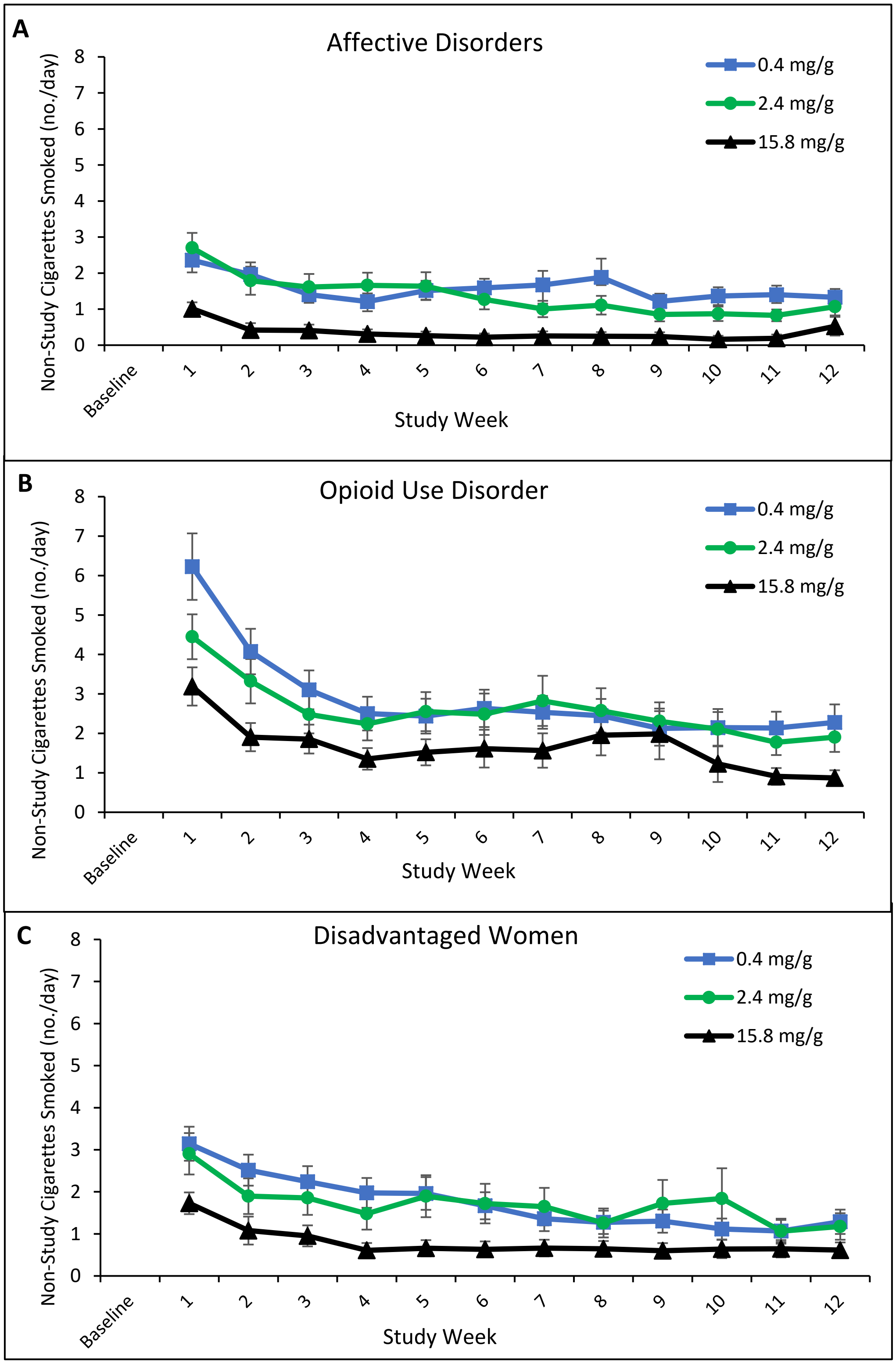

eFigure 1. Panels show non-study cigarettes smoked per day (CPD) for each population. Each panel shows the three nicotine-content-cigarette doses (0.4, 2.4, and 15.8 mg/g) across study weeks for each study population. Data points are arithmetic means collapsed across participants; error bars represent ± SEM. Analysis of this variable required use of a negative reciprocal transformation of the time variable (weeks) in order to allow modeling of a linear relationship of CPD over time.

© 2020 Higgins ST et al. *JAMA Network Open.*

eFigure 2. Wisconsin Index of Smoking Dependence and Motivation

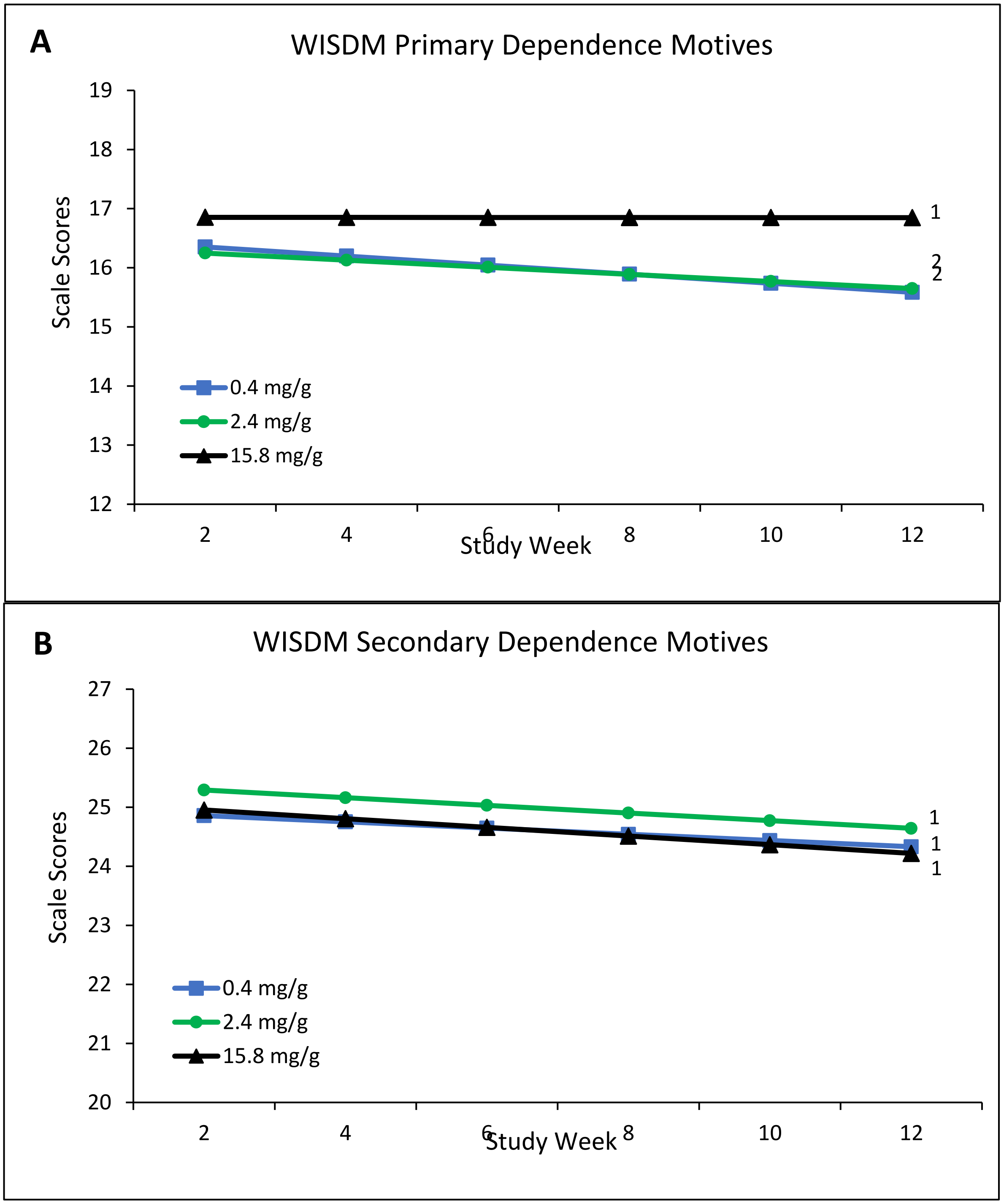

eFigure 2. Panels show linear trendlines, derived from linear regression prediction equations, for the Wisconsin Index for Smoking Dependence (WISDM) Primary and Secondary Dependence Motives subscales across study weeks for each nicotine-content dose condition. Trendlines not sharing a superscript number had significantly different slopes between doses across the 12-week study period.

© 2020 Higgins ST et al. *JAMA Network Open.*

eFigure 3. Effects of Dose and Time on Amplitude and Persistence of Demand from the Cigarette Purchase Task

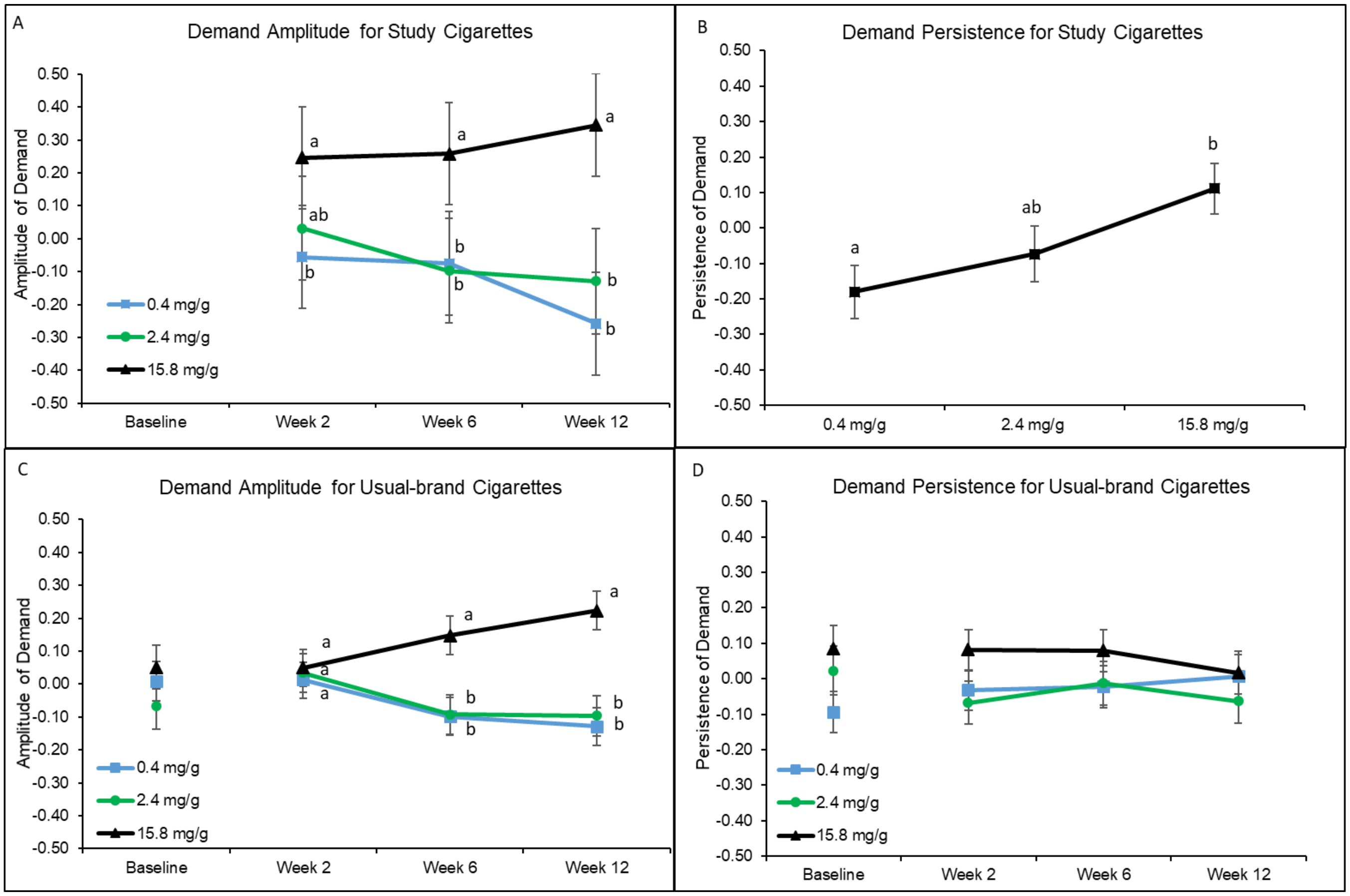

eFigure 3. Upper left panel (A) shows Cigarette Purchase Task (CPT) mean latent-factor scores for Amplitude of consumer demand for study cigarettes at each dose (0.4, 2.4, and 15.8 mg/g) by study CPT-assessment weeks (Weeks 2, 6, and 12). Data points are arithmetic means collapsed across participants and populations; error bars represent ± SEM. Data points not sharing a superscript letter differed significantly by dose in post-hoc testing at each assessment time. Upper right panel (B) shows CPT mean latent-factor scores for Persistence of consumer demand for study cigarettes by dose. Data points are arithmetic means collapsed across participants, time, and populations; error bars represent ± SEM. Data points not sharing a superscript letter differed significantly in post-hoc testing between doses. Lower left panel (C) shows CPT mean latent-factor scores for Amplitude of consumer demand for usual-brand cigarettes at each dose at baseline and by study weeks. There was a significant interaction of dose and time ($P < .001$). Data points not sharing a superscript letter differed significantly by dose in post-hoc testing at that assessment time. Lower right panel (D) shows CPT mean latent-factor scores for Persistence of consumer demand for usual brand cigarettes at each dose at baseline and by study weeks. There were neither significant effects of dose nor interactions of dose by time or population on demand Persistence for usual-brand cigarettes.

© 2020 Higgins ST et al. *JAMA Network Open.*

eFigure 4. Questionnaire of Smoking Urges: Factor 1

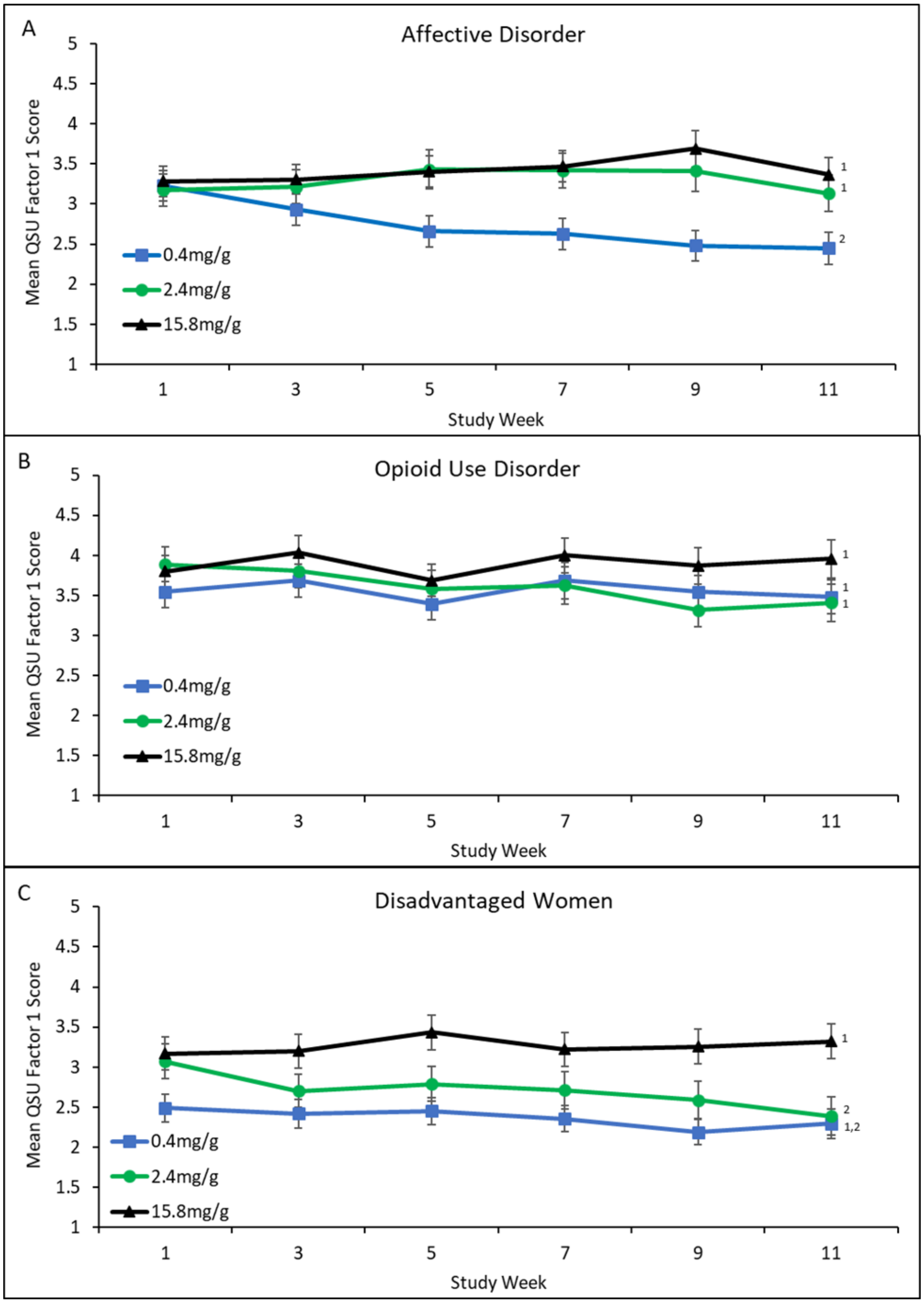

eFigure 4. Panels show mean values for Factor 1 of the Questionnaire of Smoking Urges for each population as a function of study cigarette dose (0.4, 2.4, and 15.8 mg/g) by study weeks. Error bars represent ± SEM. Data points not sharing a superscript number differed significantly by dose in slope of linear trends across the 12-week study period.

© 2020 Higgins ST et al. *JAMA Network Open.*

eFigure 5. Questionnaire of Smoking Urges: Factor 2

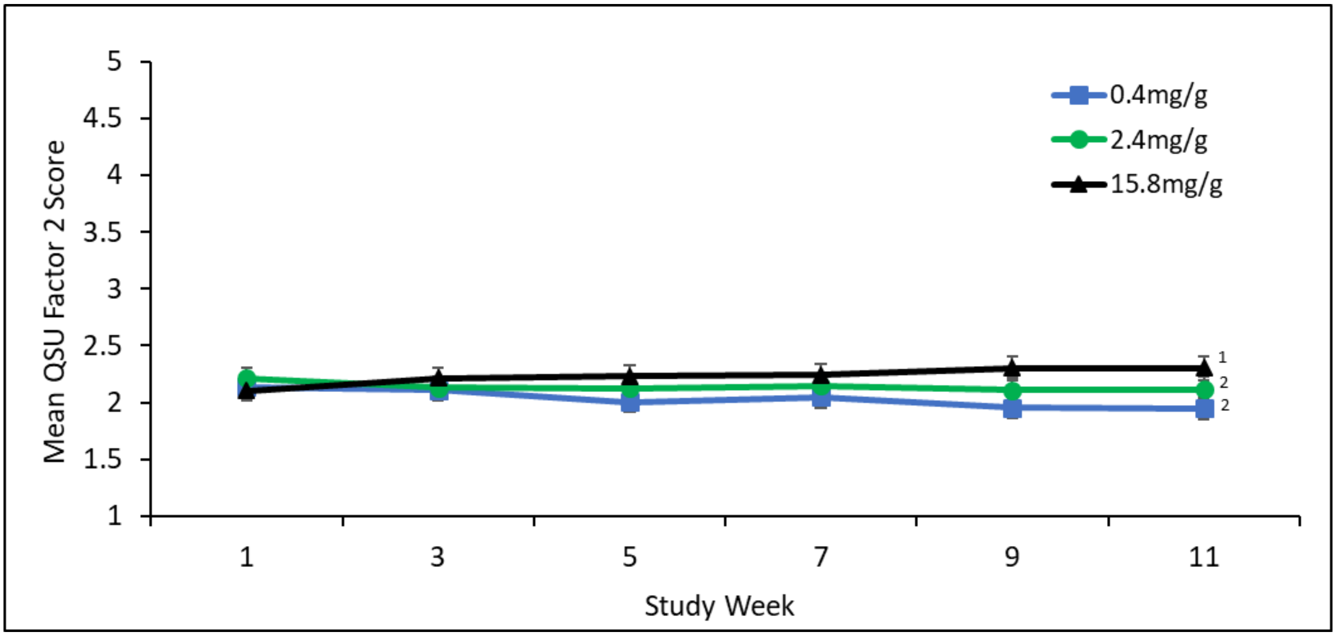

eFigure 5. Panel shows mean values for Factor 2 of the Questionnaire of Smoking Urges for the three doses collapsed across participants and populations; error bars represent ± SEM. Data points not sharing a superscript number differed significantly by dose in slope of linear trends across the 12-week study period.

© 2020 Higgins ST et al. *JAMA Network Open.*

eFigure 6. Beck Depression Inventory Score by Dose

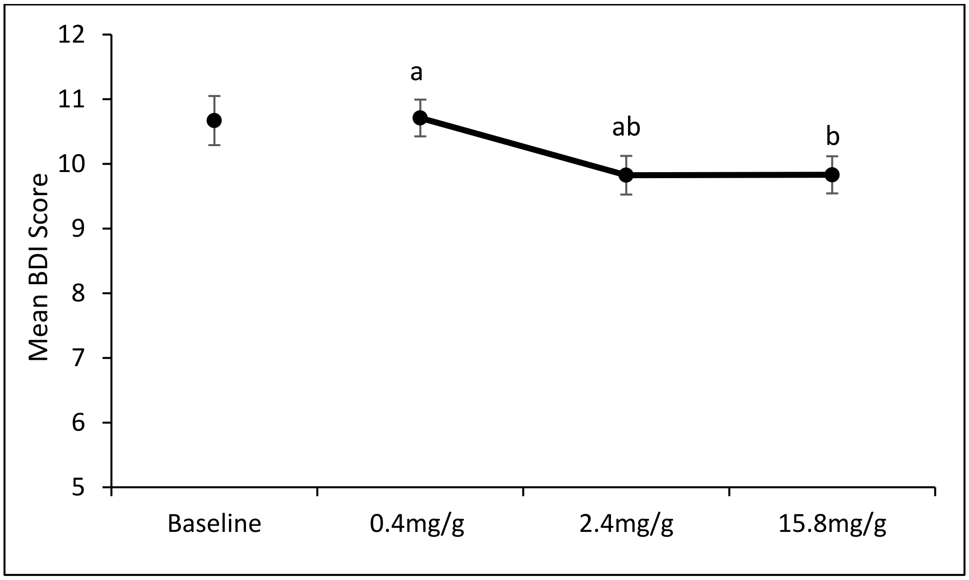

eFigure 6. Panel shows Beck Depression Inventory (BDI) Scores for each of the three nicotine-content-cigarette doses (0.4, 2.4, and 15.8 mg/g). Data points are collapsed across participants, populations, and 12-week study period; error bars represent ± SEM. Data points not sharing a superscript letter differed significantly by dose.

© 2020 Higgins ST et al. *JAMA Network Open.*

eTable 1. Demographic and Smoking Characteristics by Completer Status

	Overall	Completers	Non-completers
	**n = 775**	**n = 642**	**n = 133**
Population			
Disadvantaged Women	257 (33.16)	212 (33.02)	45 (33.83)
Opioid Use Disorder	260 (33.55)	214 (33.33)	46 (34.59)
Affective Disorders	258 (33.29)	216 (33.64)	42 (31.58)
Age (M ± SD)	35.59 ± 11.05	36.30 ± 11.17	32.17 ± 9.81*
Gender (% Female)	551 (71.10)	462 (71.96)	89 (66.92)
Race/Ethnicity			
Non-Latino White	630 (82.14)	515 (80.97)	115 (87.79)
Non-Latino Black	68 (8.87)	59 (9.28)	9 (6.87)
Latino	23 (3.00)	19 (2.99)	4 (3.05)
Non-Latino Other or >1 race	46 (6.00)	43 (6.76)	3 (2.29)
Education			
8th Grade or Less/Some High School	102 (13.16)	88 (13.71)	14 (10.53)
High School Graduate /Equivalent	570 (73.55)	465 (72.43)	105 (78.95)
2-Year Associate Degree	38 (4.90)	34 (5.30)	4 (3.01)
College Graduate/4-Year Degree	65 (8.39)	55 (8.57)	10 (7.52)
Marital Status			
Married	112 (14.45)	102 (15.89)	10 (7.52)**
Never Married	461 (59.48)	374 (58.26)	87 (65.41)**
Divorced or Separated/Widowed	202 (26.06)	166 (25.86)	36 (27.07)**
Primary Smoker of Mentholated Cigarettes	351 (45.29)	287 (44.70)	64 (48.12)
Cigarettes Smoked Per Day (M ± SD)	17.79 ± 9.18	17.83 ± 9.28	17.62 ± 8.68
Urine Cotinine Level, ng/ml (M ± SD)	4929.35 ± 3771.79	4947.49 ± 3751.98	4839.34 ± 3882.35
Nicotine Metabolite Ratio (% with NMR ≥ 0.31)	526 (73.06)	439 (72.20)	87 (77.68)
Breath Carbon Monoxide level (M ± SD)	18.02 ± 9.85	18.20 ± 9.88	17.15 ± 9.67
Age Started Smoking Regularly (M ± SD)	16.14 ± 3.97	16.21 ± 4.03	15.80 ± 3.66
Fagerstrom Test for Nicotine Dependence (M ± SD)	5.56 ± 2.35	5.59 ± 2.34	5.41 ± 2.42
Heaviness of Smoking Index (M ± SD)	3.49 ± 1.54	3.52 ± 1.52	3.34 ± 1.66
Used Other Tobacco Products, Last 30 days	117 (17.67)	100 (18.28)	17 (14.78)

Note: Unless otherwise indicated, data are expressed as number (%). *Indicates significant difference by completer status, Kruskal-Wallis Chi-Square = 16.59, $p < 0.001$. **Indicates Chi-Square = 6.35, p=0.04.

© 2020 Higgins ST et al. *JAMA Network Open.*

eTable 2. Participant Inclusion Criteria by Study Population

Inclusion Criteria	Disadvantaged Women of Childbearing Age	Smokers with Opioid Use Disorder	Smokers with Affective Disorders
Report smoking ≥ 5 cigarettes per day for the past year	X	X	X
Provide an intake breath Carbon Monoxide sample > 8 ppm or NicAlert Strip > 2	X	X	X
No current (past year) serious mental disorder that would interfere with study results or completion	X	X	X
No current substance abuse/dependence other than nicotine	X		X
Sufficiently literate to complete the research-related tasks	X	X	X
Good physical health without serious illness or change in health or medication in the past three months	X	X	X
Not pregnant or nursing and report using barrier, oral, implant, patch, ring, injection, or implant contraceptives OR report being surgically sterile or post-menopausal	X	X	X
No significant use of other tobacco or nicotine products within the past month (> 9 days in the past 30)	X	X	X
Women ages 18-44 years, highest academic degree ≤ high school	X		
Receiving methadone or buprenorphine maintenance treatment for opioid use disorder, maintained on stable dose for at least 30 days, and no evidence of regular illicit use (>30% positive illicit of past month samples)		X	
Affective disorder in past year based on MINI structured interview OR lifetime diagnosis of affective disorder with self-report of current treatment			X

© 2020 Higgins ST et al. *JAMA Network Open.*

eTable 3. Participant Exclusion Criteria by Study Population

Exclusion Criteria	Disadvantaged Women of Childbearing Age	Smokers with Opioid Use Disorder	Smokers with Affective Disorders
Any prior regular use (use as primary cigarette outside of lab) of Spectrum cigarettes	X	X	X
Exclusive use of roll-your-own cigarettes	X	X	X
Planning quit attempt in the next 30 days	X	X	X
Past 30 day quit attempt with > 3 days abstinence	X	X	X
Currently use of anticonvulsant medications	X	X	X
Positive toxicology screen for illicit drug use (cocaine, opiates, oxycodone, methadone, buprenorphine, benzodiazepines, barbiturates, amphetamines, methamphetamines, MDMA, PCP)	X	X	X
Breath alcohol level at intake > 0.01	X	X	X
Self-report of binge drinking (4 drinks for females or 5 drinks for males in a 2-hour period on > 9 days in the past 30)	X	X	X
Systolic blood pressure < 90 or ≥ 160 mmHg	X	X	X
Diastolic blood pressure < 50 or ≥ 100 mmHg	X	X	X
Heart rate < 45 or ≥ 115 bpm	X	X	X
Breath Carbon Monoxide > 80 ppm	X	X	X
Currently seeking treatment for smoking cessation	X	X	X
Use of nicotine replacement, bupropion, or other pharmacotherapies as cessation aids in the past month	X	X	X
Current symptoms of psychosis, dementia or mania	X	X	X
Past month suicidal ideation	X	X	X
Suicide attempt in past 6 months	X	X	X
Participation in another research study in the past 30 days	X	X	X
Co-habitation with any current or former participant provided with Spectrum cigarettes to smoke outside the research lab	X	X	X
Not currently enrolled in a treatment program for opioid dependence and/or not currently stable on their methadone or buprenorphine dose		X	
Unstable psychiatric conditions (defined as psychiatric medication changes in the past 30 days)			X

© 2020 Higgins ST et al. *JAMA Network Open.*

eTable 4. Minnesota Tobacco Withdrawal Scale Total Score by Dose

	**0.4 mg/g**	**2.4 mg/g**	**15.8 mg/g**
Baseline	1.05 (0.05)	1.02 (0.06)	1.11 (0.06)
Week 1	1.31 (0.06)	1.24 (0.06)	1.26 (0.06)
Week 3	1.29 (0.06)	1.23 (0.06)	1.25 (0.06)
Week 5	1.27 (0.06)	1.19 (0.06)	1.25 (0.06)
Week 7	1.24 (0.06)	1.19 (0.06)	1.20 (0.06)
Week 9	1.17 (0.06)	1.14 (0.07)	1.21 (0.06)
Week 11	1.19 (0.06)	1.14 (0.07)	1.17 (0.07)

Note: Values are means (SEM).

© 2020 Higgins ST et al. *JAMA Network Open.*

eTable 5. Smoking Topography and Before to After Smoking Breath Carbon Monoxide Change

	**0.4mg/g**	**2.4mg/g**	**15.8mg/g**
Total Puff Volume			
Week 2	1357.73 (312.13)	1202.15 (313.87)	1290.96 (313.75)
Week 6	1168.44 (310.23)	1230.95 (312.08)	1152.62 (311.48)
Week 12	1175.10 (311.54)	1210.68 (313.75)	1193.43 (312.45)
Carbon Monoxide Change			
Week 2	3.04 (0.51)	3.59 (0.52)	3.21 (0.51)
Week 6	3.35 (0.52)	3.34 (0.53)	3.34 (0.52)
Week 12	4.20 (0.52)	4.23 (0.52)	3.91 (0.52)

Note: Values are mean (SEM). Total Puff Volume is represented in milliliters (mL). Carbon monoxide change represents the difference between measurements pre-smoking and fifteen minutes post-smoking.

© 2020 Higgins ST et al. *JAMA Network Open.*

eTable 6. Total Number of Adverse Events

Adverse Event Description	Overall	0.4 mg/g	2.4 mg/g	15.8 mg/g
Total Number of Events	3177	1155	943	1079
Increased CPD > 100%	63	9	18	36
Increased BDI	385	144	125	116
Increased Oasis/Endorsed "4" on any	437	162	125	150
Blood and lymphatic system disorders	2	0	1	1
Cardiac disorders	36	12	9	15
Ear and labyrinth disorders	9	3	2	4
Endocrine disorders	1	0	0	1
Eye disorders	14	5	7	2
Gastrointestinal disorders	281	118	71	92
General disorders and administration site conditions	234	85	72	77
Hepatobiliary disorders	1	1	0	0
Immune system disorders	9	4	2	3
Infections and infestations	344	121	106	117
Injury, poisoning and procedural complications	71	30	13	28
Investigations	12	7	3	2
Metabolism and nutrition disorders	25	10	7	8
Musculoskeletal and connective tissue disorders	128	41	34	53
Neoplasms benign, malignant and unspecified	5	2	3	0
Nervous system disorders	178	70	52	56
Pregnancy, puerperium and perinatal conditions	1	1	0	0
Psychiatric disorders	348	126	103	119
Renal and urinary disorders	15	4	5	6
Reproductive system and breast disorders	26	8	9	9
Respiratory, thoracic and mediastinal disorders	337	114	111	112
Skin and subcutaneous tissue disorders	30	12	8	10
Social circumstances	3	0	1	2
Surgical and medical procedures	86	32	25	29
Vascular disorders	96	34	31	31

Note: Adverse event category descriptions are from the Common Terminology Criteria of Adverse Events (CTCAE) v5.0 developed by the National Cancer Institute with the exception of "Increased BDI", "Increased CPD > 100%", and "Increased OASIS", which were adverse event categories specific to the current clinical trial and populations. Detailed descriptions of each individual event were recorded and monitored by the PI and licensed medical professional at each site until resolved (see eSupplement 1 for protocol details)

© 2020 Higgins ST et al. *JAMA Network Open.*

eTable 7. Number of Participants with Any Adverse Event

Adverse Event Description	Overall		0.4 mg/g		2.4 mg/g		15.8 mg/g	
	n	%	n	%	n	%	n	%
	670	86.5%	241	84.3%	203	86.4%	226	89.0%
Increased BDI	244	31.5%	91	31.8%	79	33.6%	74	29.1%
Increased CPD > 100%	54	7.0%	8	2.8%	16	6.8%	30	11.8%
Increased OASIS	294	37.9%	105	36.7%	87	37.0%	102	40.2%
Blood and lymphatic system disorders	2	0.3%	0	0.0%	1	0.4%	1	0.4%
Cardiac disorders	30	3.9%	11	3.8%	9	3.8%	10	3.9%
Ear and labyrinth disorders	9	1.2%	3	1.0%	2	0.9%	4	1.6%
Endocrine disorders	1	0.1%	0	0.0%	0	0.0%	1	0.4%
Eye disorders	12	1.5%	5	1.7%	5	2.1%	2	0.8%
Gastrointestinal disorders	190	24.5%	78	27.3%	49	20.9%	63	24.8%
General disorders and administration site conditions	171	22.1%	67	23.4%	46	19.6%	58	22.8%
Hepatobiliary disorders	1	0.1%	1	0.3%	0	0.0%	0	0.0%
Immune system disorders	8	1.0%	4	1.4%	2	0.9%	2	0.8%
Infections and infestations	262	33.8%	92	32.2%	79	33.6%	91	35.8%
Injury, poisoning and procedural complications	54	7.0%	19	6.6%	13	5.5%	22	8.7%
Investigations	11	1.4%	7	2.4%	2	0.9%	2	0.8%
Metabolism and nutrition disorders	25	3.2%	10	3.5%	7	3.0%	8	3.1%
Musculoskeletal and connective tissue disorders	103	13.3%	33	11.5%	27	11.5%	43	16.9%
Neoplasms benign, malignant and unspecified	5	0.6%	2	0.7%	3	1.3%	0	0.0%
Nervous system disorders	136	17.5%	49	17.1%	38	16.2%	49	19.3%
Pregnancy, puerperium and perinatal conditions	1	0.1%	1	0.3%	0	0.0%	0	0.0%
Psychiatric disorders	228	29.4%	84	29.4%	66	28.1%	78	30.7%
Renal and urinary disorders	13	1.7%	4	1.4%	5	2.1%	4	1.6%
Reproductive system and breast disorders	23	3.0%	7	2.4%	7	3.0%	9	3.5%
Respiratory, thoracic and mediastinal disorders	234	30.2%	84	29.4%	72	30.6%	78	30.7%
Skin and subcutaneous tissue disorders	29	3.7%	12	4.2%	8	3.4%	9	3.5%
Social circumstances	3	0.4%	0	0.0%	1	0.4%	2	0.8%
Surgical and medical procedures	65	8.4%	26	9.1%	16	6.8%	23	9.1%
Vascular disorders	67	8.6%	25	8.7%	22	9.4%	20	7.9%

Note: Adverse event category descriptions are from the Common Terminology Criteria of Adverse Events (CTCAE) v5.0 developed by the National Cancer Institute with the exception of "Increased BDI", "Increased CPD > 100%", and "Increased OASIS", which were adverse event categories specific to the current clinical trial and populations. Detailed descriptions of each individual event were recorded and monitored by the PI and licensed medical professional at each site until resolved (see eSupplement 1 for protocol details)

© 2020 Higgins ST et al. *JAMA Network Open.*

eTable 8. Count of Serious and Severe Adverse Events

Description	Overall	0.4 mg/g	2.4 mg/g	15.8 mg/g
Number of serious adverse events[a]	30	13	4	13
Events related, probably related or possibly related	3	0	0	3
Number of severe adverse events	85	32	19	34
Events related, probably related or possibly related	10	3	2	5

Note: [a] Adverse events that are related to a single episode or multiple adverse events of the same type are counted only once**.**

© 2020 Higgins ST et al. *JAMA Network Open.*

eTable 9. Description of Serious and Severe Adverse Events

Seriousness	Severity	Treatment Group	Type of Event
Serious	Death	0.4 mg	Death
Serious	Life threatening	15.8 mg	Hyperglycemia; Type 2 diabetes diagnosis
Serious	Life threatening	15.8 mg	Type 2 myocardial infarction
Serious	Life threatening	15.8 mg	Acute renal failure
Serious	Moderate	0.4 mg	[a]Frontal lobe lesion
Serious	Severe	0.4 mg	Neurosurgery
Serious	Moderate	0.4 mg	Anxiety: PTSD from domestic assault
Serious	Severe	0.4 mg	Kidney infection
Serious	Moderate	0.4 mg	[b]Seizure
Serious	Moderate	0.4 mg	Seizure
Serious	Moderate	0.4 mg	Seizure
Serious	Severe	0.4 mg	Alcohol abuse
Serious	Severe	0.4 mg	Seizure
Serious	Severe	0.4 mg	Stroke
Serious	Severe	0.4 mg	Diarrhea, hospitalized
Serious	Severe	0.4 mg	Vomiting, hospitalized
Serious	Severe	0.4 mg	Bloody stool, hospitalized
Serious	Severe	0.4 mg	Stomach pain, hospitalized
Serious	Moderate	0.4 mg	Increased anxiety
Serious	Moderate	0.4 mg	Alcohol abuse, relapse, hospitalized
Serious	Moderate	0.4 mg	Head injury, hospitalized
Serious	Severe	0.4 mg	Broken right foot
Serious	Moderate	0.4 mg	Fractured right ankle
Serious	Moderate	0.4 mg	Fractured right ribs
Serious	Moderate	0.4 mg	Chipped left elbow
Serious	Moderate	0.4 mg	Sprained left knee
Serious	Severe	0.4 mg	Laceration of tendon of left thumb
Serious	Severe	0.4 mg	Laceration of artery of left thumb
Serious	Severe	2.4 mg	Surgery for skin graft from thigh to graft onto a long-existing wound on shin
Serious	Severe	2.4 mg	Repeat surgery for skin for a long-existing wound on shin
Serious	Severe	2.4 mg	Skin infection/cellulitis
Serious	Severe	2.4 mg	Chest pain - cardiac
Serious	Severe	2.4 mg	Depression, self-admitted to the hospital
Serious	Severe	15.8 mg	Increased depression based on Beck Depression Inventory score
Serious	Severe	15.8 mg	Hospitalization for infection and flexor tenosynovitis secondary to a dog bite, allowing

© 2020 Higgins ST et al. *JAMA Network Open.*

			a pre-existing skin infection to enter the participant's blood stream.
Serious	Moderate	15.8 mg	Blurred vision
Serious	Severe	15.8 mg	Hypertension - primary care visit and diagnosis.
Serious	Severe	15.8 mg	Emergency room visit for increased pain from migraines, fibromyalgia, back and neck joint pain, degenerative disk disease.
Serious	Severe	15.8 mg	Psychosis
Serious	Severe	15.8 mg	Tenosynovitis of hand
Serious	Severe	15.8 mg	Cellulitis of hand
Serious	Severe	15.8 mg	Hepatitis B infection
Serious	Severe	15.8 mg	Hepatitis C infection
Serious	Severe	15.8 mg	Emergency room visit for chest pain.
Not Serious	Severe	0.4 mg	Worsening pancreatitis, hospitalized
Not Serious	Severe	0.4 mg	Worsening pancreatitis, ER visit for pain
Not Serious	Severe	0.4 mg	Migraine headache
Not Serious	Severe	0.4 mg	Increased hip pain, ER visit
Not Serious	Severe	0.4 mg	Hypertension
Not Serious	Severe	0.4 mg	Hypertension
Not Serious	Severe	0.4 mg	Hypertension
Not Serious	Severe	0.4 mg	Kidney stones
Not Serious	Severe	0.4 mg	Head injury - ER visit.
Not Serious	Severe	0.4 mg	Suicidal ideation
Not Serious	Severe	0.4 mg	Hypertension
Not Serious	Severe	0.4 mg	Hypertension
Not Serious	Severe	0.4 mg	Hypertension
Not Serious	Severe	0.4 mg	Hypertension
Not Serious	Severe	0.4 mg	Hypertension
Not Serious	Severe	0.4 mg	Tooth infection
Not Serious	Severe	0.4 mg	Hypertension
Not Serious	Severe	0.4 mg	Tonsillar abscess
Not Serious	Severe	0.4 mg	ER Visit for flu-like symptoms
Not Serious	Severe	0.4 mg	Hypertension
Not Serious	Severe	0.4 mg	Hypotension
Not Serious	Severe	0.4 mg	Hypertension
Not Serious	Severe	0.4 mg	Hypertension
Not Serious	Severe	0.4 mg	Stomach virus
Not Serious	Severe	0.4 mg	Vomiting
Not Serious	Severe	0.4 mg	Gum infection
Not Serious	Severe	0.4 mg	Vomiting
Not Serious	Severe	0.4 mg	Hypertension
Not Serious	Severe	0.4 mg	Hypertension
Not Serious	Severe	0.4 mg	Syncope
Not Serious	Severe	0.4 mg	Hypertension

© 2020 Higgins ST et al. *JAMA Network Open.*

Not Serious	Severe	0.4 mg	Increased anxiety based on OASIS score
Not Serious	Severe	0.4 mg	Increased anxiety based on OASIS score
Not Serious	Severe	0.4 mg	Increased depression based on BDI score
Not Serious	Severe	0.4 mg	Stomach pain/cramps
Not Serious	Severe	0.4 mg	Skin infection/cellulitis
Not Serious	Severe	0.4 mg	Increased depression based on BDI score
Not Serious	Severe	0.4 mg	Increased anxiety based on OASIS score
Not Serious	Severe	0.4 mg	Increased CPD more than 100% of baseline
Not Serious	Severe	2.4 mg	Kidney stones
Not Serious	Severe	2.4 mg	Laceration of thumb, lost part of thumb.
Not Serious	Severe	2.4 mg	Muscle weakness in lower limb.
Not Serious	Severe	2.4 mg	Difficulty focusing vision
Not Serious	Severe	2.4 mg	Broken arm
Not Serious	Severe	2.4 mg	Hypertension
Not Serious	Severe	2.4 mg	Hypertension
Not Serious	Severe	2.4 mg	Hypertension
Not Serious	Severe	2.4 mg	Hypertension
Not Serious	Severe	2.4 mg	Hypertension
Not Serious	Severe	2.4 mg	Hypertension
Not Serious	Severe	2.4 mg	Depression
Not Serious	Severe	2.4 mg	Hypertension
Not Serious	Severe	2.4 mg	Hypertension
Not Serious	Severe	2.4 mg	Hypertension
Not Serious	Severe	2.4 mg	Anxiety/recurring panic attacks
Not Serious	Severe	2.4 mg	Panic attack
Not Serious	Severe	2.4 mg	Hypertension
Not Serious	Severe	2.4 mg	Hypertension
Not Serious	Severe	2.4 mg	Hypertension
Not Serious	Severe	2.4 mg	Increased anxiety based on OASIS score
Not Serious	Severe	2.4 mg	Increased depression based on BDI score
Not Serious	Severe	2.4 mg	Increased pain in left arm
Not Serious	Severe	2.4 mg	Increased anxiety
Not Serious	Severe	15.8 mg	Torn ligament in knee
Not Serious	Severe	15.8 mg	Apnea after taking medication
Not Serious	Severe	15.8 mg	Kidney stones
Not Serious	Severe	15.8 mg	Pain (muscle/back/stomach spasms)
Not Serious	Severe	15.8 mg	Syncope
Not Serious	Severe	15.8 mg	Endometriosis
Not Serious	Severe	15.8 mg	Laparoscopy
Not Serious	Severe	15.8 mg	Hypertension
Not Serious	Severe	15.8 mg	Hypertension
Not Serious	Severe	15.8 mg	Hypertension

© 2020 Higgins ST et al. *JAMA Network Open.*

Not Serious	Severe	15.8 mg	Tooth infection
Not Serious	Severe	15.8 mg	Surgery to remove infected bone in upper jaw.
Not Serious	Severe	15.8 mg	ER visit for vomiting
Not Serious	Severe	15.8 mg	Hypertension
Not Serious	Severe	15.8 mg	Hypertension
Not Serious	Severe	15.8 mg	Hypertension
Not Serious	Severe	15.8 mg	Hypertension
Not Serious	Severe	15.8 mg	Tooth fillings
Not Serious	Severe	15.8 mg	Tooth fillings
Not Serious	Severe	15.8 mg	Hypertension
Not Serious	Severe	15.8 mg	Depression
Not Serious	Severe	15.8 mg	Upper respiratory infection
Not Serious	Severe	15.8 mg	Hypertension
Not Serious	Severe	15.8 mg	Hypertension
Not Serious	Severe	15.8 mg	Hypertension
Not Serious	Severe	15.8 mg	ER visit for hypertension
Not Serious	Severe	15.8 mg	PCP visit and diagnosis of hypertension
Not Serious	Severe	15.8 mg	Hypertension
Not Serious	Severe	15.8 mg	Hypertension
Not Serious	Severe	15.8 mg	Hypertension
Not Serious	Severe	15.8 mg	Worsening pain (associated with migraines, fibromyalgia, deg. disk disease, back/neck pain)
Not Serious	Severe	15.8 mg	Hypertension
Not Serious	Severe	15.8 mg	Back pain
Not Serious	Severe	15.8 mg	Manic episode
Not Serious	Severe	15.8 mg	Increased anxiety based on OASIS score
Not Serious	Severe	15.8 mg	Increased depression based on BDI score
Not Serious	Severe	15.8 mg	Increased depression based on BDI score
Not Serious	Severe	15.8 mg	Facial cellulitis
Not Serious	Severe	15.8 mg	Tooth infection
Not Serious	Severe	15.8 mg	Constipation
Not Serious	Severe	15.8 mg	Broken ribs
Not Serious	Severe	15.8 mg	Increased anxiety based on OASIS score

Note: [a] Entries shaded in dark gray indicate adverse events related to a single episode. [b] Entries shaded in light gray indicate adverse events of the same type but occurring separated in time.

© 2020 Higgins ST et al. *JAMA Network Open.*
