# Supplementary material for: Changes in Cigarette Consumption With Reduced Nicotine Content Cigarettes Among Smokers With Psychiatric Conditions or Socioeconomic Disadvantage: 3 Randomized Clinical Trials
Source: JAMA Netw Open. 2020 Oct 20;3(10):e2019311. doi: 10.1001/jamanetworkopen.2020.19311 (PMC7576411; doi:10.1001/jamanetworkopen.2020.19311)
Supplement: Supplement 3. — Data Sharing Statement [file jamanetwopen-e2019311-s003.pdf]

Data Sharing Statement

Higgins. Changes in Cigarette Consumption With Reduced Nicotine-Content Cigarettes Among Smokers With Psychiatric Conditions or Socioeconomic Disadvantage. *JAMA Netw Open*. Published October 20, 2020. 10.1001/jamanetworkopen.2020.19311

Data
Data available: Yes
Data types: Deidentified participant data
How to access data: First author (Stephen T. Higgins): stephen.higgins@uvm.edu
When available: beginning date 01-01-2023

Supporting Documents
Document types: Other (please specify)
Additional Information: Supplemental Figures and Tables.
How to access documents: Supplemental Figures and Tables.
When available: beginning date: 01-01-2023

Additional Information
Who can access the data: anyone requesting the data
Types of analyses: specified purpose
Mechanisms of data availability: without investigator support **Any additional restrictions:** Data will be available from the National Institute of Drug Abuse's datasharing site (https://datashare.nida.nih.gov/index.php/)
